# Virtual reality in the assessment, understanding, and treatment of mental health disorders

**DOI:** 10.1017/S003329171700040X

**Published:** 2017-03-22

**Authors:** D. Freeman, S. Reeve, A. Robinson, A. Ehlers, D. Clark, B. Spanlang, M. Slater

**Affiliations:** 1 Department of Psychiatry, University of Oxford, Oxford, UK; 2 Oxford Health NHS Foundation Trust, Oxford, UK; 3 Department of Experimental Psychology, University of Oxford, Oxford, UK; 4 Event Lab, Department of Clinical Psychology and Psychobiology, University of Barcelona, Barcelona, Spain; 5 Institució Catalana de Recerca i Estudis Avançats (ICREA), Barcelona, Spain

**Keywords:** Assessment, mental health, theory, treatment, virtual reality (VR)

## Abstract

Mental health problems are inseparable from the environment. With virtual reality (VR), computer-generated interactive environments, individuals can repeatedly experience their problematic situations and be taught, via evidence-based psychological treatments, how to overcome difficulties. VR is moving out of specialist laboratories. Our central aim was to describe the potential of VR in mental health, including a consideration of the first 20 years of applications. A systematic review of empirical studies was conducted. In all, 285 studies were identified, with 86 concerning assessment, 45 theory development, and 154 treatment. The main disorders researched were anxiety (*n* = 192), schizophrenia (*n* = 44), substance-related disorders (*n* = 22) and eating disorders (*n* = 18). There are pioneering early studies, but the methodological quality of studies was generally low. The gaps in meaningful applications to mental health are extensive. The most established finding is that VR exposure-based treatments can reduce anxiety disorders, but there are numerous research and treatment avenues of promise. VR was found to be a much-misused term, often applied to non-interactive and non-immersive technologies. We conclude that VR has the potential to transform the assessment, understanding and treatment of mental health problems. The treatment possibilities will only be realized if – with the user experience at the heart of design – the best immersive VR technology is combined with targeted translational interventions. The capability of VR to simulate reality could greatly increase access to psychological therapies, while treatment outcomes could be enhanced by the technology's ability to create new realities. VR may merit the level of attention given to neuroimaging.

## Introduction

A technological revolution in mental health care is approaching. At the forefront may be virtual reality (VR), a powerful tool for individuals to make new learning for the benefit of their psychological well-being. Immersive VR creates interactive computer-generated worlds, which substitute real-world sensory perceptions with digitally generated ones, producing the sensation of actually being in life-sized new environments. VR allows such tight control over the stimuli presented that therapeutic strategies can be precisely implemented; VR can produce situations that can be therapeutically helpful if used in the right way but near impossible to recreate in real life; VR allows repeated, immediately available and greater treatment input; and VR can reduce inconsistency of treatment delivery. With high-quality VR devices reaching the consumer market for the first time, the future is suddenly imminent. The affordability makes it feasible for the technology to break out of the laboratory and enter the home – and forward-thinking mental health clinics too.

### VR

The basic elements of VR – a computer generating an image, a display system presenting the sensory information, and a tracker feeding back the user's position and orientation in order to update the image – have existed for 50 years. The hardware recognizable today emerged in the 1980s but has been largely confined to specialist laboratories (Slater & Sanchez-Vives, 2016). Systems vary greatly. For example, the Cave Automatic Virtual Environment (CAVE) projects computer images onto the walls of a room and the participant wears tracked shutter glasses to view the scene three-dimensionally (Cruz-Neira *et al*. 1993). Sometimes described as VR are much lower-specification systems that use displays on computer monitors or large projector screens, but the limited levels of immersion and interaction make it questionable whether these are truly VR. The current excitement relates to the new generation of head-mounted display (HMD) and associated equipment that have emerged as affordable consumer products due to the investment of global companies. Smartphones, laptops or desktop computers can run the software. VR is moving out of specialist laboratories. The transformation in what the hardware and software can now realize compared with even a few years ago is great.

An HMD displays images, one for each eye, forming an overall stereo scene. Each image is computed and rendered separately with correct perspective from the position of each eye with respect to a mathematical description of a three-dimensional (3D) virtual scene. The HMD is typically tracked, with continuous capturing of the position and orientation of the participant's head and therefore head-based gaze direction. As participants turn or move their head to look around, the computer updates at a very high frame rate – typically 60 frames per second – the images displayed. Therefore participants see a surrounding 3D stereo scene that can change dynamically. Although much VR programming has omitted it, one particular object in the scene can have special status – the virtual body of the participant. At its simplest, this visual substitution of the person's real body can be aligned to the head tracking. But if the participant wears a motion-tracking capture suit with an HMD, then the data from this, continuously streamed to the computer, will effectively substitute the body of the participant by a life-sized virtual body that fully moves in correspondence with their own movements, leading to the illusion of body ownership (Slater *et al.* 2010; Spanlang *et al.* 2014).

Perception through natural movement is the key element of an immersive VR system. Immersion reflects the system's technical capabilities; the subjective experience delivered is termed ‘presence’, which is the illusion of being in the place rendered by VR. Presence comprises two concepts: place illusion (PI) and plausibility illusion (Psi) (Slater, 2009). PI is the sense of being in the virtual place. A necessary condition for PI is that the VR is perceived through natural sensorimotor contingencies, based on the active vision paradigm (Noë, 2004). The idea of this paradigm is that we perceive through using our whole body, via a set of implicit rules involving head turning, leaning, reaching, looking around and so on. The illusion of ‘being there’ is generated to the extent that the VR system affords perception through such contingencies. If what we see matches our movements then the brain's conclusion is that these are our surroundings. The Psi is the sense that the events experienced in VR are happening (e.g. that there are people walking about, that a ball is flying through the air), even though, of course, individuals consciously know that these are not real. Psi requires that the virtual environment responds to actions of the participants, generates spontaneous actions towards them, and is ecologically valid when real-life events are depicted. For example, when the environment includes virtual human characters, these avatars should respond to the presence and actions of the participants (e.g. by gaze and maintaining appropriate interpersonal distances). When both PI and Psi operate, participants will be likely to behave realistically in VR.

### VR and mental health

VR has extraordinary potential to help people overcome mental health problems if high levels of presence are achieved for situations that trouble them. Difficulties interacting in the world are at the heart of mental health issues [e.g. becoming highly anxious near spiders in arachnophobia, having intense flashbacks with reminders of past trauma in post-traumatic stress disorder (PTSD), fearing attack from people in persecutory delusions, resisting the urge to take another drink in alcohol abuse disorders]. Therefore recovery concerns thinking, reacting and behaving differently in these situations. The most successful interventions are those that enable people to make such changes in real-world situations. With VR, individuals can enter simulations of the difficult situations and be coached in the appropriate responses, based upon the best theoretical understanding of the specific disorder. The simulations can be graded in difficulty and repeatedly experienced until the right learning is made. Problematic situations difficult to find in real life can be realized at the flick of a switch. And the great advantage of VR is that individuals know that a computer environment is not real but their minds and bodies behave as if it is real; hence, people will much more easily face difficult situations in VR than in real life and be able to try out new therapeutic strategies. The learning can then transfer to the real world. For some disorders it may be possible to eradicate the need for any therapist input, while for other disorders the time required of skilled therapists could be greatly reduced. Thus VR could help improve access to the most effective psychological treatments. It may become the method of choice for psychological treatment: out with the couch, on with the headset.

There are also many other potential uses of VR in mental health. We originally set out seven purposes (Freeman, 2008): symptom assessment, identification of symptom markers or correlates, establishment of factors predictive of disorders, tests of putative causal factors, investigation of the differential prediction of symptoms, determination of toxic elements in the environment, and the development of treatment. For instance, standard mental health diagnosis chiefly comprises retrospective recall using clinician interview and validated questionnaires. Inevitably, human beings tend to be very subjective in their views. Memory, moreover, is notoriously fallible. In the clinic of the future, it is possible that problems could also be assessed live in VR. The technology could also help make substantial inroads into understanding the causes of mental health disorders, for example, pinpointing the environmental characteristics that raise the risk of adverse psychological reactions in the context of individual differences.

The aim of this paper is to highlight for clinicians and researchers in mental health the potential of VR technology. This includes a review of what has been learned empirically from the first generation of studies about the use of VR in assessing, understanding and treating the main adult mental health disorders. We wished to identify established findings, obvious areas of neglect and new directions of interest. These are the studies that have been conducted in specialist laboratories over the past 20 years, before the current transformation in availability and capabilities of the technology. It is the ambition of these pioneering studies that we aim to capture, as new hardware and software are dramatically altering what can be created in VR and the ease of use.

## Method

The literature on VR in mental health was searched up to the end of 2016. The inclusion criteria were: a specific focus on the assessment, theory, or treatment of adult mental health disorders; published in a peer-reviewed journal; was an empirical study (including case studies with data); used a form of immersive VR (HMD, CAVE, large projection screen, screen with 3D glasses); and in the English language. The exclusion criteria were: non-immersive VR method [e.g. personal computer (PC) screen only, websites such as Second Life]; qualitative data or review; unable to obtain the paper; use of VR but no specific focus on a mental health symptom or condition. We did not look at health psychology, cognitive disorders, personality disorders, childhood-onset disorders, or at the effects of VR games that were not designed as interventions.

PubMed was used for the searches, which were conducted separately by major disorder types. The general search terms were: [([Virtual reality OR Immersive virtual reality] AND [Assessment OR treatment OR research OR study OR experiment OR understanding]) AND (disorder-specific terms inserted here)] AND English (language). For anxiety disorders, the search terms added were: (Anx* OR obsessive-compulsive OR post-traumatic OR panic OR social phobia OR social anxiety OR phob*OR GAD OR OCD OR PTSD OR SAD). For depression, the search terms added were: (depression OR depress*). For psychosis the search terms added were: (Delus*OR Hallucinat* OR Psychosis OR Psychotic OR Schizophren* OR Schizotyp* OR Bipolar OR Mania OR Manic). For substance disorders, the search terms added were: (substance disorder OR substance abuse OR substance OR abuse OR cannabis OR tobacco OR alcohol OR amphetamine OR hallucinogens OR heroin). For eating disorders, the search terms added were: AND (anorexia nervosa OR bulimia nervosa OR eating disorders OR binge eating). For sleep disorders, the added terms were: (insomnia OR sleep OR nightmares OR circadian). For sexual disorders, the added search terms were: (sexual OR orgasm OR desire OR erectile OR ejaculation OR dyspareunia). Titles and abstracts were read, and, if appropriate, the whole paper, in order to determine whether the inclusion and exclusion criteria were met.

## Results

A total of 1096 studies were identified from the literature searches, of which 285 met the review inclusion criteria. Summaries of the searches by each of the disorder types are displayed in the online Supplementary Figs S1–S7. Descriptions of the individual VR studies are available in the online Supplementary Tables S1–S7. The most common reason for an empirical study to be excluded from review was the use of non-immersive technology (e.g. participants simply viewed a standard computer screen for presentation) or empirical data were not reported.

### Anxiety disorders

Overwhelmingly, VR studies have concerned the treatment of anxiety disorders (*n* = 127 intervention reports). Even the assessment studies (*n* = 46) have mainly been conducted for the purpose of validating VR environments for treatment. The use of VR to investigate the causes of anxiety has been more rarely conducted (*n* = 19). The focus of the treatment studies has typically been on specific phobias (e.g. Rothbaum *et al.* 2000; Emmelkamp *et al.* 2002; Garcia-Palacios *et al.* 2002; Botella *et al.* 2004) or social anxiety (e.g. Anderson *et al.* 2013; Bouchard *et al.* 2016) or post-traumatic stress disorder (e.g. Difede *et al.* 2007; Rizzo *et al.* 2009), with many fewer investigations for obsessive– compulsive disorder (OCD), which is surprising given that treatment often requires change in fears about external stimuli, and generalized anxiety disorder, which is less surprising given the internal focus of the disorder. The principal intervention technique has been exposure, with a therapist present to guide the person in most of the intervention studies. The treatment studies have undoubtedly been pioneering in recognizing the potential for the technology in this treatment area especially (e.g. Hodges *et al.* 1995; Rothbaum *et al.* 1996; Botella *et al.* 1998). Case study reports or small randomized controlled trials have dominated the field. The quality of studies has too often been low (Meyerbröker & Emmelkamp, 2010; McCann *et al.* 2014). There are too few convincing randomized controlled trials, although this is beginning to change (e.g. Anderson *et al.* 2013; Bouchard *et al.* 2016; Reger *et al.* 2016), too few experimental studies of potential treatment mediators (e.g. Shiban *et al.* 2016), and comparisons between different techniques have typically been underpowered. Overall, however, VR treatments seem to perform comparably in efficacy to face-to-face equivalent interventions. With the caveat concerning the quality of the studies, the treatment efficacy has been shown in meta-analyses to be large (e.g. Opriş *et al.* 2012), with evidence that the beneficial effects transfer to the real world (Morina *et al.* 2015). When long-term follow-ups have been included, treatment effects for these short-term therapies have strikingly been shown to persist over a number of years (e.g. Rothbaum *et al.* 2002; Wiederhold & Wiederhold, 2003). There are indications that drop-out rates may be lower with VR treatments but that may simply reflect a problem of quality control with face-to-face therapy delivery. The range of VR-type methods used has been wide, varying from large projection screens to the computer-assisted research environment (CAREN) system, in which the person has a walking platform surrounded by a 360° display, to CAVEs, flight simulators, and to HMDs. Not all reports make clear which type of technology was used. The greater the sense of presence in VR achieved then the more likely anxiety will occur (Ling *et al*. 2014). The importance of sound in achieving presence in VR should not be overlooked (e.g. Taffou *et al*. 2013). Detailed studies of how best to present stimuli in VR are warranted but in our opinion have been far too rare (e.g. Shiban *et al*. 2015).

### Depression

Surprisingly, we identified only two studies that clearly used immersive VR in relation to depression. These feasibility studies tested out single treatment techniques in small case series with no control conditions (Shah *et al*. 2015; Falconer *et al*. 2016), with levels of depression found to decrease with time. There have also been two studies of (non-immersive) VR-type tasks assessing spatial navigation memory in patients with depression (e.g. Gould *et al*. 2007).

### Psychosis

There have been 44 VR studies about schizophrenia and related problems, with 23 concerning theory development, 15 concerning assessment, and six testing treatment. The types of VR studies here were probably the most heterogeneous compared with the other mental health conditions, reflecting the complexity of the clinical problem and the different perspectives taken towards diagnosing and understanding psychosis. The studies have predominately used VR to assess psychotic experiences in order to understand the causes. VR has been of particular use in assessing paranoia because the presentation of neutral social situations enables unfounded, rather than genuine, hostility to be detected. It is clear that VR can safely assess psychotic experiences in patients with schizophrenia and related diagnoses. Our group pioneered the work on VR in relation to paranoia. We have used VR to: assess paranoia (e.g. Freeman *et al*. 2003); understand the individual characteristics predictive of paranoia (e.g. Freeman *et al*. 2008); manipulate psychological factors in order to determine the causes of paranoia (e.g. Freeman *et al*. 2014); and, most recently, treat persecutory delusions in the context of schizophrenia (Freeman *et al*. 2016). A rare use of VR to create a situation that cannot be achieved in real life is provided by our manipulation of a person's height in order to affect self-esteem and hence paranoia (Freeman *et al*. 2014). The small treatment study with 30 patients with persecutory delusions showed that VR cognitive therapy is potentially much more efficacious than VR exposure therapy both in terms of reducing delusions and lessening distress in real-world situations. The controlled effect size (*d* = 1.3) for VR cognitive therapy was large, which is notable given that it was compared with another credible treatment approach. VR has also been used to study environmental factors that make an impact on paranoia, by altering variables such as population density and ethnicity (e.g. Valmaggia *et al*. 2015; Veling *et al*. 2016). There is also a strand of VR work assessing cognitive and social functioning in schizophrenia (e.g. Sorkin *et al*. 2006) and consequent intervention (e.g. Rus-Calafell *et al*. 2014). Treatment studies are generally very few in number and small in size but the results are very encouraging. No studies related to mania were identified.

### Substance disorders

VR has the potential to present individuals with simulations of the cues that lead to the cravings that drive subsequent problematic behaviours such as drug misuse, alcohol abuse or excessive gambling. There have been 22 VR studies on substance disorders, with 15 concerning assessment, five treatment, and two theory development. The overwhelming majority of the studies have simply shown that appropriate VR environments can trigger cravings. Misuse of a range of substances has been studied, including alcohol (e.g. Lee *et al*. 2008) and cocaine (e.g. Saladin *et al*. 2006). However, the majority of the work has concerned smoking (e.g. Bordnick *et al*. 2005) and it is evident that VR environments can produce strong cravings for cigarettes (Pericot-Valverde *et al*. 2016). The elicitation of cravings means that VR has the potential to be successfully used in treatment, though this has not yet been rigorously demonstrated. Uncontrolled studies do indicate that VR might be able to help reduce cravings for cigarette smoking (e.g. Pericot-Valverde *et al*. 2014). Even crushing virtual cigarettes has been found to be helpful when added to standard treatment (Girard *et al*. 2009). For smoking cessation, a randomized controlled trial testing cognitive–behavioural therapy (CBT) with VR cue exposure is underway (Giovancarli *et al*. 2016).

### Eating disorders

There are a number of obvious mechanistic targets for VR in the treatment of eating disorders: reducing food cravings, improving body image, and enhancing emotion regulation skills. A total of 18 empirical studies were identified, 10 concerning treatment, seven assessment, and one theory development. Despite an early use of VR for eating disorders (e.g. Riva, 1998), it has been recognized that the field has very few methodologically strong studies (Riva, 2011; Ferrer-García & Gutiérrez-Maldonado, 2012). Suitable VR environments can bring on food cravings (e.g. Ferrer-García *et al*. 2015), with responses to VR food comparable with real food (Gorini *et al*. 2010), and there has even been an initial test of high-calorie food presented using augmented reality (Pallavicini *et al*. 2016). The preliminary trial evidence is that VR techniques added to standard CBT help to improve body image (Riva *et al*. 2003; Cesa *et al*. 2013; Marco *et al*. 2013). In an intriguing VR experimental study, Keizer *et al.* (2016) helped patients with anorexia nervosa to experience ownership of a healthy-body mass index (BMI) body, which led afterwards, for at least 2 h, to a reduction in body size overestimation. New research on understanding the body ownership illusion in VR is likely to enhance eating disorder treatments (Normand *et al*. 2011; Maselli & Slater, 2013).

### Additional disorders

VR could have potential uses in the understanding and treatment of sexual disorders concerning desire, arousal and orgasm. This work has not been carried out. The literature search revealed four reports describing a series of uncontrolled studies in which a form of psychodynamic therapy for erectile dysfunction or premature ejaculation included a VR element (e.g. Optale *et al*. 2003). Another notable area is sleep disorder, which is very common in the general population, but VR has not been used to study causes or treatments. Three studies have used a VR paradigm (road crossing) to assess the adverse effects of sleep disorders for the daytime safety of children (e.g. Avis *et al*. 2014).

## Discussion

We conclude from the early studies that VR environments can elicit psychiatric symptoms, manipulation of VR can inform the understanding of disorders, and simpler psychological treatments can be successfully administered in VR. This is highly encouraging for the future application of VR to mental health. However, our inspection of the older literature warns that the technology of VR is not an answer in and of itself: the content delivered will matter for outcomes (e.g. Freeman *et al*. 2016; Reger *et al*. 2016). Across a breadth of disorders there are instances of real innovations in the interaction between the technology and insights into mental health problems. This has largely been unheralded, perhaps because the methodological quality has been limited and the potential for wider dissemination hitherto constrained. The studies have typically been small, negative results are less likely to have been reported, and, in most places, the literature has been distinctly piecemeal. Progress has been understandably slow because hardware and software have been expensive and expertise limited. This is about to change (Wiederhold, 2016).

The gaps in the literature are astonishingly large. This technology has simply not been applied enough to mental health. Psychiatric symptoms can be assessed in VR, but robust tests of reliability and validity have been very few; compared with retrospective self-report, VR has the potential to prove a ‘gold standard’ assessment method for many mental health problems but this has not remotely been tested. VR has been used to develop the understanding of too few disorders, although even when used as an investigative tool it has principally been used to assess symptoms rather than provide firmer causal conclusions via manipulation tests (Cook & Campbell, 1979). Treatment trials have been small in size, rarely pre-registered, and seldom conducted to the standards now expected in clinical research. Of the range of treatment techniques available it is the simpler ones, such as exposure, that have been used. A therapist has nearly always still been engaged in the VR interventions. Numerous other important treatment techniques remain to be implemented in VR, especially for more complex disorders. There is an intriguing programme of research to be conducted concerning the degree to which therapies can be delivered without a therapist present for each type of presenting problem, and whether avatars can compensate for the important human presence fundamental to traditional psychological interventions. Many common disorders, for example, depression, have barely received any VR research attention. VR also has obvious, but untested, use in psychiatric settings such as hospital wards or forensic units where contact with the outside world is highly restricted.

We believe that there are three overarching treatment questions that need to be addressed: (1) What is the best way to immerse individuals in VR so that learning most readily transfers to the real world, balancing the need to use affordable equipment? (2) Can key theory-driven psychological treatment techniques (beyond simple exposure) be successfully delivered in VR? (3) Do engaging, personalized, theory-driven treatments implemented in affordable VR, with limited use of clinicians, produce large real-world benefits for patients? This work will need to be carried out with the user experience put at the centre of design. Given its use in gaming, VR could be made a highly appealing treatment approach for patients. There is also the issue of how related technologies, notably augmented reality and wearable devices, could dovetail with the new approaches.

Our review offers, perhaps, a glimpse of the future of mental health care. It is, however, still relatively early days with VR for mental health: scenarios are limited, as is the degree of social interaction, for example conversation, that is possible. Specialist programming expertise is still required to create suitable environments that lead to presence. Simulator sickness may occur in poorly realized scenarios and systems. Multi-sensory presentation of stimuli is most likely to induce presence, but generalized touch feedback, that is tactile stimulation on any part of the body contingent on collision with a virtual object, is not feasible at present. The potential therapeutic power of body ownership manipulation remains confined to specialist laboratories. But the technology is developing fast: these are likely to be short-term concerns. Psychological research and clinical practice have made huge strides in recent years too (Layard & Clark, 2015). We now have a much clearer picture of which therapeutic techniques are most effective, but suitably trained therapists are in short supply, and quality control remains a concern. VR and related technologies could help in solving this problem, making the best therapy available to many more people. Yet the power of VR is such that it promises much more than an improved delivery method for psychological therapies. VR allows us to try things that are not easily practical in the real world. That means it could potentially generate the kind of results that even a course of standard treatment could not produce. ‘Revolutionary’ is an overused word; for VR and mental health care, it may actually be justified over the coming years.

## Supplementary material

For supplementary material accompanying this paper visit http://dx.doi.org/10.1017/S003329171700040X. click here to view supplementary material
