# Supplementary material for: Virtual reality in the assessment, understanding, and treatment of mental health disorders
Source: Psychol Med. 2017 Mar 22;47(14):2393–400. doi: 10.1017/S003329171700040X (PMC5964457; doi:10.1017/S003329171700040X)
Supplement: Supplementary file 1 [file S003329171700040Xsup001.zip › S003329171700040Xsup001.doc]

**Anxiety disorders**

**Supplementary Fig. S1.** PRISMA 2009 Flow Diagram

**Screening**

**Included**

**Eligibility**

**Identification**

Records identified through database searching
(n = 549)

Additional records identified through other sources
(n = 40) from citations )

Records screened
(n = 589)

Records excluded
(n = 302)

Full-text articles assessed for eligibility
(n = 287 )

Full-text articles excluded, with reasons
(n = 95 )

Not immersive VR = 12

No data = 38

Not on anxiety = 23

Not peer reviewed journal =12

Duplicates: 7

Not English language = 3

Studies included in qualitative synthesis
(n = 192 )

**Supplementary Table S1.** *Anxiety disorder studies*

| **Authors** | **Disorder** | **Category** | **N** | **Participant characteristics** | **Virtual Reality type** | **Aim/Hypothesis** | **Conclusions** |
| --- | --- | --- | --- | --- | --- | --- | --- |
| **1. Ahs, F. et al. (2015)** | Anxiety | Theory | 43 | Right-handed non-clinical adults. | MRI compatible 3D goggles. | Investigated the idea that anxiety disorders are maintained by extinction memory deficits that results in fear renewal. Used fMRI to further understand neural mechanisms of context-dependent fear renewal. | Results suggested the effect of the hippocampus on right amygdala activity during fear renewal was mediated by dmPFC, whereas the effect of the hippocampus on right amygdala activity during extinction recall was partially mediated by the vmPFC. Demonstrates dissociable contextual influences of the hippocampus on prefrontal pathways that are responsible for and determine the level of reactivation of fear associations |
| **2. Alsina-Jurnet, I. et al. (2007)** | Anxiety | Assessment | 21 | University students aged 18-38 with high (n =11) or low (n =10) anxiety test scores. | PC screen with I-visor personal display and head tracker. | Aimed to validate the effectiveness of virtual environments (student's home, metro, corridor, exam lecture hall) designed to produce emotional responses in those high levels of anxiety, ultimately to be implemented in treatment. | Higher levels of subjective and state anxiety, and depression were reported in the high-test-anxiety group, suggesting that the VR environment was able to activate the emotional structure of the high-test group and induce emotional responses similar to those seen in real life. |
| **3. Alsina-Jurnet, I. et al. (2010)** | Anxiety | Assessment | 210 | University students aged 18-45. | PC screen with I-visor personal display and head tracker. | Investigated the influence of test anxiety, spatial intelligence, verbal intelligence, personality and computer experience on sense of presence. | Test anxiety environments elicited emotional responses in both groups and these were similar to those experienced in real world environments. Those within high test anxiety group with higher scores on spatial intelligence or introversion experienced greater presence in emotionally significant VR environment. |
| **4. Alsina-Jurnet, I. et al. (2011)** | Anxiety | Assessment | 210 | University students aged 18-45. | PC screen with I-visor personal display and head tracker. | Considered relationship between in-session anxiety and sense of presence in VR, as sense of presence is a key mechanism that enables the experience of anxiety in virtual environments. | Presence was not found to be related to anxiety in a non-stressful environment. Stronger relationship between presence in clinical environments and anxiety in high-test anxiety group |
| **5. Anderson, P. et al. (2013)** | Social anxiety | Treatment | 97 | Met criteria for social anxiety disorder | HMD - VR environments with varying numbers of audience members, participant had to give speech. | RCT comparing VR exposure therapy with in vivo exposure for SAD with a primary fear of public speaking. Participants randomly assigned to VR exposure therapy (VRE), exposure group therapy (EGT) or wait list, with 8 sessions in therapy groups. Reactions and number of audience members manipulated. | Those in treatment groups significantly improved on all but one measure of social anxiety, maintained at 12 month follow up. Suggests VRE is as equally effective as EGT. |
| **6. Anderson, P. et al. (2005)** | Social Phobia | Treatment | 10 | Met criteria for social phobia or panic disorder with agoraphobia. 7 met criteria for another anxiety disorder. | HMD - VR environments with varying numbers of audience members, participant had to give speech. | Aimed to test use of VR exposure as a CBT treatment for public-speaking anxiety. 4 sessions of anxiety management training, 4 sessions of VRE. Reactions and number of audience members manipulated. | Self-reported anxiety measures decreased post-treatment and were maintained at 3 month follow up, but treatment did not change likelihood of a participant completing a speech. |
| **7. Anderson, P. et al. (2003)** | Social Phobia | Treatment | 2 | Met diagnostic criteria for social phobia with prominent public speaking fears. | HMD with head tracking - VR audience. | Explored use of VR as a medium for exposure therapy; 1 participant underwent weekly psychotherapy, 1 underwent a 3-day intensive course of therapy. Reactions of audience manipulated. | Self-reported anxiety measures decreased post-treatment. Follow up data from one participant demonstrated continued improvement after treatment. |
| **8. Aymerich-Franch, L. et al. (2014)** | Anxiety | Assessment | a) PILOT - 252 b) Study 1 - 82 c) Study 2 - 105 | a) Volunteers aged 18-74.  b) University students aged 18-32 c) University students aged 18-39 | HMD with head and wrist tracking - VR audience. | Pilot and 2 lab experiments:  Pilot - participants selected an avatar to use in a VR public speaking task.  Study 1 - speech in front of VR audience as an avatar representative of self or an avatar whose face was not own.  Study 2 - Further investigated anxiety differences with self-representative or dissimilar avatar. | Higher social anxiety was associated with stronger preference for embodying an avatar dissimilar to self during a VR speech. However, in study 1, whilst there was a trend for lower anxiety with an assigned (not representative) face, overall, assigning participants a dissimilar face did not significantly reduce their anxiety when giving a speech in VR. Study 2 did find that assigning a dissimilar avatar reduced anxiety relative to self avatar for one anxiety measure. |
| **9. Banos, R. et al. (2011)** | PTSD | Treatment | 39 | Diagnosis of PTSD, Pathological grief or Adjustment disorder. | 2 PCs, 1 large projection screen, 2 projectors. | Use of VR to explore negative experiences of those with PTSD/PG/AD. Compared standard CBT program with VR CBT 'EMMA's World'. | Results suggest VR CBT was as similarly effective as standard CBT, with better improvements on depression, relaxation intensity and social area interference variables in VR condition. |
| **10. Banos, R. et al. (2002)** | Phobia - Flying | Treatment | 4 | Met criteria for specific phobia, situational type (FP). | HMD with head tracking - 3 VR environments concerning travelling on plane (planning, airport, on board). | Explore use of VR exposure therapy for flying phobia. Unlike other programs, also focused on anticipatory anxiety. | VR environment was able to induce anxiety, prolonged exposure resulted in decreased anxiety measure scores (fear, avoidance, beliefs, expectations etc.). All participants were later able to make a real flight with minimal anxiety. |
| **11. Beck, J. et al. (2007)** | PTSD | Treatment | 6 | Experienced motor vehicle accident involving actual or threatened death and that evoked an emotional response. | 10x8ft projection screen, stereoscopic glasses, motion base outfitted with steering wheel, gas pedal, brake pedal. Driving scenarios. | Explore the use of VRET for the treatment of PTSD following a motor accident. 10 sessions. | Meaningful and reliable reductions in post trauma symptoms following treatment in all participants. High levels of presence and satisfaction with VR system treatment. |
| **12. Botella, C. et al. '05** | Phobia - Cockroaches | Treatment | 1 | Met criteria for phobia to small animals, specifically fear of cockroaches. | AR using HMD. Cockroaches manipulated in number and size. | Case study - Use of AR for cockroach phobia. One session treatment. | Decrease in fear and avoidance when faced with virtual cockroaches, able to interact with and kill real cockroach following treatment. Improvements maintained at 2 month follow up. |
| **13. Botella, C. et al. '98** | Phobia - Claustrophobia | Treatment | 1 | Strong fear, anxiety and inability to undergo an important scan. Historical fear of closed places. | HMD with head tracking - VR room environments. | Case study - Explore the efficacy of VR exposure for the treatment of claustrophobia. 8 sessions. | Decreased anxiety scores on self-report measures following treatment. |
| **14. Botella, C. et al. '99** | Phobia - Claustrophobia | Treatment | 1 | Met criteria for 2 specific phobias - situational type (claustrophobia) and natural environment type (storms). Also met criteria for panic disorder with agoraphobia. | HMD with head tracking - VR room environments. | Case study - Explore the efficacy of VR exposure for a patient with 2 specific phobias (claustrophobia and storms) . 8 sessions. | Decreased fear and avoidance to claustrophobic situations as well as generalisation of improvements to other specific phobic and agoraphobic situations not directly treated - maintained at 3 month follow up. |
| **15. Botella, C. et al. '04** | Phobia - Flying | Treatment | 9 | Met criteria for specific phobia, situational type (FP). 2 also suffered from panic disorder, 2 presented with panic disorder and agoraphobia. | HMD with head tracking - 3 VR environments concerning travelling on plane (planning, airport, on board). | Explored the short and long term efficacy of VRET in the treatment in flying phobia. | VR environment was able to induce fear and avoidance responses, with prolonged exposure resulting in a decrease of self-report measures of flying phobia including fear, avoidance and belief in catastrophic thoughts. All participants were able to fly following treatment. |
| **16. Botella, C. et al. '00** | Phobia - Claustrophobia | Treatment | 4 | Fear of closed spaces; 1 = met criteria for specific phobia, situational type (claustrophobia), 3 = met criteria for PD with agoraphobia. | HMD with head tracking - VR house and elevator. | Aimed to determine efficacy of VR exposure in treatment of claustrophobic fear. Hypothesised that due to the similarity between claustrophobia and PD with agoraphobia, similar treatment effects would be seen. | Decrease in all clinical measures following treatment, suggesting it was successful, with changes maintained at follow up. |
| **17. Botella, C. et al. '10** | Phobia - Cockroaches | Treatment | 6 | Females meeting criteria for specific phobia, animal type, specifically cockroach. | AR using HMD. Cockroaches manipulated in number and size. | Explored the short term and long term efficacy of AR used in the treatment of cockroach phobia. | AR system was able to induce anxiety in all participants, with prolonged exposure resulting in reduction in fear level, avoidance and belief in negative thoughts related to main target behaviour. Improvements maintained at 3, 6 and 12 month follow ups. |
| **18. Botella, C. et al. '10** | PTSD | Treatment | 10 | Met criteria for PTSD. 1 participant co-morbid with dysthymia, 1 co-morbid with OCD. Half taking medication. Varying experienced traumas. | 2 PCs, 1 large projection screen, 2 projectors. | Investigated efficacy of 'EMMA's World' VR adaptive display combined with CBT for the treatment of diverse trauma PTSD victims. Randomly assigned to either CBT + 'EMMA's World' or standard CBT. | CBT + 'EMMA's World' was as equally effective as standard CBT in resulting in a reduction in PTSD symptoms. |
| **19. Botella, C. et al. '16** | Phobia - Small Animal | Treatment | 63 32 = AR system, 31 = in vivo exposure | Met criteria for specific phobia (animal subtype) to cockroaches or spiders, at least 1 year duration | AR using HMD or VR goggles. Spiders and cockroaches manipulated in number and size. | Explored the efficacy and acceptance of 2 different exposure treatments for specific phobias, comparing AR exposure and in vivo exposure in an RCT. | Both treatments resulted in significant improvements in primary and secondary outcome measures (fear, avoidance, anxiety, belief, severity, performance etc.), and were maintained at 3 and 6 month follow up. Significant differences between groups concerning avoidance post-treatment, but this difference was not observed at follow ups. Positive feedback from participants - considered AR exposure to be less aversive. |
| **20. Bouchard, S. et al (2006)** | Phobia - Arachnophobia | Treatment | 11 | Met criteria for arachnophobia and unable to go through all 10 steps of the BAT. | HMD with head tracking and joystick. Initial neutral VR environment, with gradual exposure to spider environment. | Explored the effectiveness of use of VR in the treatment of arachnophobia using modified 3D game ('Half-Life'). | Significant improvements post-treatment on self-report and Behavioural measures of spider phobia - many able to touch spider with pencil. Significantly higher self-efficacy following treatment. |
| **21. Bouchard, S. et al (2008)** | Phobia - Snakes | Treatment | 31 15 = group 1, 16 = group 2 | Adults suffering from phobia of snakes | HMD with head tracking and joystick. Egyptian desert, told which condition were entering. | Explored the direction of the causal relationship between anxiety and presence. Randomized, between-subjects design consisting of 2 conditions and 3 counterbalanced immersions; baseline, threatening/anxiety-inducing environment (contained snakes), non-threatening environment. | Induction of anxiety in VR resulted in stronger feelings of presence during VR immersion, suggesting that state anxiety has a direct impact on subjective feeling of presence. However, large changes in anxiety were associated with smaller changes in presence, and scores of participants' presence questionnaires demonstrated decreased presence with increased anxiety - a contradictory finding. |
| **22. Bouchard et al (2016)** | Social anxiety | Treatment | 59 | Diagnosis of social anxiety disorder. | HMD | Randomised controlled trial: CBT with in vivo exposure compared against CBT with VR exposure against waiting list control. | CBT using VR was more effective than standard CBT. |
| **23. Breton-Lopez, J. et al. (2010)** | Phobia - Cockroaches | Assessment | 6 | Females meeting criteria for specific phobia, animal type, specifically cockroach. | AR using HMD. Cockroaches manipulated in number, size and movement. | Sought to validate whether stimuli used in AR-Insect phobia system would be capable of inducing anxiety in participants with diagnosed cockroach phobia. | System was able to induce anxiety in all participants, with some elements more central to this - "giving movement" was found to be one of the most important factors in eliciting anxiety. |
| **24. Bruce, M. et al. (2009)** | Phobia - Claustrophobia | Assessment | 16 | Non-clinical adults with no reported levels of claustrophobia. | HMD with head tracking and Xbox controller. Multiple VR room environments of differing size and features. | Investigated the efficacy of an immersive VRET prototype system for the treatment of claustrophobia. Participants used system when presented via PC screen and through HMD - randomized order. | System induced a sense of presence and levels of discomfort (anxiety and arousal) in non-clinical participants, suggesting it may be a useful tool for the treatment of claustrophobia. Users of HMD reported higher ecological validity and maintained higher levels of engagement, but also experienced slightly higher levels of negative effects. |
| **25. Burton, M. et al. (2013)** | Anxiety - SAD | Assessment | 65 | Diagnosis of SAD, with public speaking as their most feared situation. | HMD - VR environments with varying numbers of audience members, participant had to give speech. | Secondary report from RCT - participants received either VRET or exposure group therapy. Examined the relationship between mindfulness and fear of negative evaluation over duration of nonmindfulness-based CBT, predicting higher mindfulness would be associated with more positive treatment outcomes. | Mindfulness was found to be related to fear, but was not a moderator of symptom reduction. Suggests SCT alone does not influence the mindfulness levels of those with SAD. |
| **26. Cardenas, G. et al. (2009)** | Phobia - Flying | Assessment | 5 | Females meeting criteria for specific phobia, situational type (FP). | HMD with head tracking - 3 VR environments concerning travelling on plane (planning, airport, on board). | Cross-cultural validation in the Mexican population of the system developed by Botella et al. - 'Fear of Flying'. Assessment instruments were adjusted following judge evaluation of the system's clarity and cultural pertinence. | Results support the efficacy and cross-cultural validity of the system, with reduction in all phobia-related measures following treatment. |
| **27. Carlin, A. et al. (1997)** | Phobia - Arachnophobia | Treatment | 1 | Female, phobic of spiders for past 20 years. | HMD, hand sensor, 3D mouse for movement. | Case study - Investigate the efficacy of VR and mixed reality (touching real objects simultaneously seen in VR) for the treatment of arachnophobia. | Reductions in anxiety and avoidance of real spiders following treatment, suggesting it is an effective treatment. |
| **28. Choi, Y. et al. (2001)** | Phobia - Acrophobia | Treatment | 1 | Male, suffering from height phobia for past 40 years. | HMD with head tracking and driving simulator. VR scene of 4-sided open lift in a steel tower. | Explore the short-term efficacy of VR therapy for acrophobia. 6 sessions of VR, followed by in vivo exposure. | Improvements in all phobic symptoms, physical symptoms and cognitive changes following treatment. Patient able to experience real world height with little distress, and 6 month post treatment reported no fear of heights. |
| **29. Choi, Y. et al. (2005)** | Panic Disorder with agoraphobia | Treatment | 40 | Diagnosis of PDA. | *Presumed as above.* | Evaluate the efficacy of short term (4 session) experimental cognitive therapy (ExCT) compared with panic control program (PCP - 12 sessions) in the treatment of panic disorder with agoraphobia (PDA). Participants randomly assigned to experimental groups. | Significant improvements across all outcome measures in both groups following treatment. At 6 month follow up, significant differences in medication discontinuation, with more patients in PCP group discontinuing. Suggests long term effectiveness of PCP may be superior to ExCT. |
| **30. Clemente, M. et al. (2014)** | Phobia - Small Animal | Assessment | 11 | Right-handed females meeting criteria for specific phobia, animal type, specifically cockroach and spider phobia. | MRI-compatible 3D goggles. VR room environment: 'clean', 'dirty' or 'phobic' (containing small animals) conditions. | Investigated the neural correlates of small animal phobia using a VR environment. Participants explore various VR environments and have to count number of keys, whilst undergoing fMRI. | Enhanced visual attention to phobic stimuli was associated with activation in left occipital inferior lobe and superior frontal gyrus. |
| **31. Coelho, C et al. (2008)** | Phobia - Acrophobia | Assessment | 8 | Adults (aged 23-58) meeting criteria for acrophobia, reporting fear of heights for 10-55 years | HMD with head tracking - VR balcony with view. | Used data from Coelho, C. et al (2006) study. Evaluated the efficacy of different types of movement in VR treatment environment. Conducted in both real and VR environments. | Increased anxiety with increased height and when participant was required to move laterally at a fixed height - more so than when viewing fear-invoking scene with no movement. Suggests a direct link between type of movement at a height and triggering of acrophobia. |
| **32. Coelho, C et al. (2006)** | Phobia - Acrophobia | Treatment | 10 | Adults aged (18-66) meeting criteria for acrophobia, reporting fear of heights for 10-55 years | HMD with head tracking - VR balcony with view. | Explored the use of VR in the treatment of acrophobia, with 1-year follow up. | Significant improvements in all phobia outcome measures (both self-report and Behavioural) following treatment. Improvements maintained at 1-year follow up, excluding the Acrophobia Questionnaire - suggests generalisation of improvements to novel situations did not occur. |
| **33. Coelho, C et al. (2008)** | Phobia - Acrophobia | Treatment | 15 | Adults aged (18-66) meeting criteria for acrophobia, reporting fear of heights for 18-55 years | HMD with head tracking - VR balcony with view. | Investigated VR and real life exposure for the treatment of acrophobia. | Significant improvements in anxiety, avoidance and behaviour measurements in both groups following treatments in both real and VR settings. Session times much shorter in VR group, suggesting that it may be more effective, as similar improvements were obtained in reduced time compared to real life exposure treatment. |
| **34. Cornwell, B. et al. (2011)** | Anxiety - SAD | Theory | a) 16 b) 16 | a) Individuals with generalised SAD b) Non-clinical controls | 3D visor - VR environment of audience exhibiting positive, neutral or hostile behaviours. | Investigated the components of public speaking that trigger fear responses in vulnerable participants. Participants gave speech to VR audience, with startle probes delivered every 17-23s 2 minutes after entering. | Greater levels of distress and state anxiety in SAD participant group. When focus of VR audience's attention, SAD group displayed potentiated startle reactivity, which suggests a strong phasic fear response. |
| **35. Cornwell, B. et al. (2005)** | Anxiety - SAD | Assessment | 45 | Non-clinical adults with range of trait anxiety levels. | 3D visor - VR environment of audience exhibiting positive, neutral or hostile behaviours. | Explored relationship between anxiety and differences in startle reactivity in socially threatening VR situations. | Startle before entering VR and during speech anticipation whilst immured in VR positively correlated with trait social anxiety. Indicates a relationship between startle reactivity and fear of negative evaluation. Anticipation whilst in VR elicited strongest physiological responses. |
| **36. Côté, S. & Bouchard, S. (2009)** | Phobia - Arachnophobia | Theory | 28 | Adults suffering from arachnophobia. | HMD with head tracking - 2 VR apartments with multiple rooms; 3 levels of difficulty (differing numbers, movement patterns and size of spiders) | Explored the cognitive mechanisms that are associated with therapeutic change following VR exposure treatment. | Changes in general outcome and cardiac response were best predicted by perceived self-efficacy and dysfunctional beliefs, change in behavioural avoidance was also predicted by changes in dysfunctional beliefs. |
| **37. Côté, S. & Bouchard, S. (2005)** | Phobia - Arachnophobia | Treatment | 28 | Adults suffering from arachnophobia. | HMD with head tracking - 2 VR apartments with multiple rooms; 3 levels of difficulty (differing numbers, movement patterns and size of spiders) | Investigated the impact of VR exposure in phobic individuals on cardiac response and autonomic processing of threatening stimuli. | Improvements in all outcome measures (questionnaire data, behaviour avoidance test, Stroop task) following treatment, suggesting VR exposure was a successful treatment. Psychophysiological data indicated decreased anxiety following treatment. |
| **38. Cristea, I. et al. (2014)** | Anxiety - SAD | Theory | 99 | Undergraduate adult volunteers scoring over 30 on the Liebowitz Social Anxiety Scale. | HMD - VR audience. | Investigated how far autonomic parameters are affected by emotion regulation strategies used by socially anxious individuals in anticipation of giving an impromptu speech to a VR audience. | All emotion regulation strategies used resulted in decreases in parasympathetic activity and increases in heart rate that remained in the recovery phase - suggests a rebound effect of social performance, as autonomic parameters indicated a feeling of stress. Acceptance was found to result in increases in parasympathetic activity more than negative functional reappraisal in high social anxiety participants. |
| **39.Czerniak, E. et al. (2016)** | Phobia - Flying | Treatment | 3 | Males suffering from fear of flying, panic attacks and other specific phobias | CAREN system - 3m diameter motion platform within dome-shaped construction, 8 projectors providing 360° display on interior surface. Participant seated on moving platform. VR scenery of takeoff, landing and air turbulence. | Evaluated the use of a large motion-based VR system for the treatment of flying phobia. | Improvements on SUD scores throughout sessions, but inconsistent trends in flight anxiety situation (FAS) and flight anxiety modality (FAM) participant scores. 2 participants were able to participant in actual flights following treatment. |
| **40. de Quervain, D. et al. (2011)** | Phobia - Acrophobia | Treatment | 40 | Met criteria for specific phobia, environmental type. | HMD with head tracking - participant stood on wooden board. VR environment of different platforms connected by bridges and elevators. | Investigated whether administration of glucocorticoid hormones would enhance the effectiveness of exposure therapy. Cortisol or placebo administered 1hr prior to entering VR treatment session. Randomised, double-blind, placebo-controlled study. | Cortisol group showed significantly greater reductions in phobic fear following treatment, maintained at 1 month follow up. Suggests cortisol can enhance VRET. |
| **41. Depperman, S. et al. (2016)** | Phobia - Arachnophobia | Theory | a) 41 b) 42 | a) Met criteria for specific spider phobia except that their day-to-day functioning was not impaired b) Non-clinical controls | HMD with head tracking - VR spider environment | Investigated the impact of intermittent theta burst stimulation (iTBS) on emotion regulation in fear-relevant situations. Applied sham-controlled activating protocol over left PFC followed by a VR challenge. Also investigated brain activations associated with being confronted with fear-inducing stimuli - fNIRS measurements taken before iTBS and after VR challenge whilst participants completed an emotional-word stroop paradigm. | Arachnophobia group displayed diminished activation in the left inferior frontal gyrus, particularly elicited by emotionally-relevant words, and also increased co-activation between the left IFG and the contra-lateral hemisphere. No significant differences in cortical activations between groups following iTBS/VR. No additional augmentation effect as a result of verum iTBS application. |
| **42. Dibbets, P. et al. (2015)** | PTSD | Assessment | 39 | Non-clinical adults volunteers. | HMD - VR environment depicting distressing scene between a man and a woman. | Aimed to develop a VR analogue to be used for PTSD, specifically the assessment of risk factors and development of PTSD-symptoms. Compared novel system to traditional trauma film paradigm (TFP). | Both conditions elicited a negative mood and induction-related intrusions in participants. Greater immersion reported in VR. |
| **43. Diemer, J. et al. (2013)** | Phobia - Arachnophobia | Treatment | 58 | Met criteria for specific phobia. | HMD with head tracking and portable game pad - 4 VR scenes: laboratory (neutral), 3 spider scenes. | Investigated the putative acute anxiolytic effects of the antipsychotic quetiapine. Used VR spider challenge with phobic participants to evaluate. Randomised, double blind, placebo-controlled study. | VR was able to induce anxiety in participants. Quetiapine group reported significant reductions in anxiety on scales measuring somatic anxiety, no significant results on other outcome measures. Significant reduction in skin conductance in experimental group also observed, with possible mediating role of COMT val158met genotype. Suggests quetiapine may be useful for treatment of somatic anxiety symptoms. |
| **44. Diemer, J. et al. (2016)** | Phobia - Acrophobia | Assessment | a) 40 b) 40 | a) Met criteria of specific phobia (heights) b) Matched non-clinical controls | HMD with head tracking - VR multistorey building. | Investigated arousal during VR exposure height challenge, assessing subjective fear ratings and physiological fear reactions. | Patient group demonstrated significant increases in subjective fear level and physiological fear measures. Controls who reported no subjective fear did display increased physiological fear responses. No cortisol response in either group. |
| **45. Difede, J. et al. (2007)** | PTSD | Treatment | 21 | Met criteria for PTSD, exposed to World Trade Centre attacks. | VR helmet with head tracking. VR scene of lower Manhattan with simulation of 9/11 attacks on WTC. | Evaluated the use of VR enhanced exposure therapy for the treatment of PTSD in those exposed to 9/11 attacks on the WTC. Flexible number of sessions. | 9/10 participants with severe PTSD showed clinically meaningful and statistically significant improvements following VR treatment. Treatment effective for participants with differing experiences of WTC attacks. |
| **46. Difede, J. et al. (2006)** | PTSD | Treatment | 17 | Met criteria for PTSD, exposed to World Trade Centre attacks. | VR helmet with head tracking. VR scene of lower Manhattan with simulation of 9/11 attacks on WTC. | Evaluated the use of VR enhanced exposure therapy for the treatment of PTSD in those exposed to 9/11 attacks on the WTC. 14 sessions. | Substantial decreases in PTSD symptoms in VR group, with 5/8 VR group participants no longer meeting criteria for PTSD following treatment. VR environment able to engage those with differing experiences of WTC attacks, overall suggesting VR may be a useful treatment tool. |
| **47. Difede, J. et al. (2014)** | PTSD | Treatment | 25 | Met criteria for PTSD, exposed to World Trade Centre attacks. | VR helmet with head tracking. VR scene of lower Manhattan with simulation of 9/11 attacks on WTC. | Investigated whether the administration of d-cycloserine (DCS) enhanced the efficacy of VRET for the treatment of PTSD. | DCS group demonstrated statistically and clinically significant greater improvement in PTSD symptoms following treatment. Greater remission rates in DCS group compared to placebo - suggests beneficial addition to VRET for PTSD. |
| **48. Difede, J. et al. (2002)** | PTSD | Treatment | 1 | Female diagnosed with PTSD and co-morbid major depression, exposed to World Trade Centre attacks. | VR helmet with head tracking. VR scene of lower Manhattan with simulation of 9/11 attacks on WTC. | Case study - Evaluated the use of VR enhanced exposure therapy for the treatment of PTSD in a female exposed to 9/11 attacks on the WTC. 6 sessions. | Reduction in acute PTSD symptoms following treatment. |
| **49. Donahue, C. et al. (2009)** | Anxiety - SAD | Treatment | 20 | Diagnosis of SAD with clinically significant public speaking fears. | HMD - VR audience, presented with one of two speech tasks. | Investigated the impact of single dose administration of quetiapine on symptoms of SAD. Ramdomised, double-blind, placebo-controlled study. | No difference in response in quetiapine group compared to control, suggesting it was not effective in alleviating SAD symptoms. |
| **50. Emmelkamp, P. et al. (2002)** | Phobia - Acrophobia | Treatment | 33 | Met criteria for acrophobia, unable to complete BAT. | HMD with head tracking - 3 VR environments (multistorey mall with escalators and balustrades, fire escape, roof garden). | Evaluate effectiveness of VR exposure compared with in vivo exposure in the treatment of acrophobia. 3 sessions. | Both groups were equally effective in decreasing anxiety and avoidance, maintained at 6 month follow up. |
| **51. Emmelkamp, P. et al. (2001)** | Phobia - Acrophobia | Treatment | 10 | Suffering from acrophobia. | HMD with head tracking - 2 VR environments (diving tower with swimming pool, tower building with glazed elevator). | Evaluate effectiveness of VR exposure compared with in vivo exposure in the treatment of acrophobia. 2 sessions of VR followed by 2 sessions of in vivo exposure. | Based on acrophobia questionnaire data, VR exposure was at least as effective as in vivo exposure, and based on attitude towards heights questionnaire data was more effective. |
| **52. Ferrand, M. et al- (2015)** | Phobia - Flying | Treatment | 145 | Non-clinical adults. | Flight simulator | Explored the effectiveness of a program developed to treat fear of flying (particular focus on flight-related anxiety) - 1 day intervention training using flight simulator and CBT, participants then took real flight. | Decrease in flight-related anxiety following treatment. |
| **53. Freedman, S. et al. (2010)** | PTSD | Treatment | 1 | Male suffering from PTSD following a terrorist bulldozer attack. | HMD - VR environment of Israel with buses. | Case study - Evaluated the use of VR in augmented imaginal exposure for the treatment of PTSD in a male exposed to a terrorist attack in Jerusalem. 3/10 sessions using VR - prolonged exposure protocol. | Large reduction in PTSD symptoms following treatment, maintained at 6-month follow up - participant no longer met criteria for PTSD following treatment. |
| **54. Freeman, D. et al. (2008)** | Anxiety - SAD | Theory | 200 | Non-clinical adult volunteers | HMD with head tracking - VR underground train journey (neutral environment). | Aimed to identify particular factors that distinguish the occurrence of social anxiety paranoid thoughts. | Social anxiety and persecutory ideation were shown to share many of the same predictive factors. Perceptual anomalies was found to be more prone in those with paranoid reactions (suggesting it is a distinct predictor), but less so in those with social anxiety. |
| **55. Freeman, D. et al. (2014)** | PTSD | Assessment | 106 | Experienced a distressing assault in the previous month. | HMD with head tracking - VR underground train journey (neutral environment). | PTSD reactions in VR used to predict later symptom severity. | VR assessment for PTSD predicted severity of PTSD over subsequent months. |
| **56. Gamito, P. et al. (2010)** | PTSD | Treatment | 10 | Elderly war veterans diagnosed with PTSD. | HMD | Evaluated VRET as a treatment for PTSD in war veterans, for the reduction of symptoms. Compared with exposure imagination (EI) and waiting list (WL) conditions. | Similar improvements in PTSD symptom seen in both VRET and EI conditions. VRET showed statistically significant reduction in anxiety and depression levels, largely maintained at 12-month follow up. |
| **57. Garcia-Palacios, A. et al. (2002)** | Phobia - Arachnophobia | Treatment | 23 | Met criteria for specific phobia, animal type - spiders. | HMD - VR spider. | Explored the use of VR exposure in the treatment of arachnophobia - tactile augmentation. | VR exposure group showed significant symptom improvement (reduction in fear and avoidance) following treatment, suggesting it is an effective treatment. |
| **58. Geradi, M. et al. (2008)** | PTSD | Treatment | 1 | Iraq war veteran with ongoing intrusive recollections of military trauma that interfered with daily life. | HMD with head tracking - VR Iraq environment. | Case study - Explored the use of VRE for the treatment of PTSD in war veteran - 4 sessions over 4 weeks. | Reduction in self-reported PTSD symptoms. Still met criteria for PTSD post-treatment, but reported experiencing improvements in functioning. |
| **59. Glotzbach-Schoon, E. et al. (2013)** | Anxiety | Theory | 49 | Student volunteers with either high anxiety (score upper 20% percentile in STAI Trait questionnaire) low anxiety (score in lower 20% percentile). | HMD with head tracking - VR room environments. | Investigated the effect of trait anxiety on contextual threat learning. Used VR as part of a contextual fear learning protocol; one VR room was paired with an unpredictable electrical stimuli (CXT+), and the other VR room paired with no electrical stimuli (CXT-). | Those in high-anxiety group displayed faster acquisition of startle potentiation in CXT+ versus CXT- conditions compared to low-anxiety group - suggests enhanced contextual fear learning. Propose this as a risk factor for anxiety disorders that feature sustained anxiety. |
| **60. Gorini, A. et al. (2010)** | Anxiety - GAD | Treatment | 21 | Diagnosis of GAD. | HMD with head tracking - VR tropical island environment. | Integrated cognitive therapy and applied relaxation using biofeedback enhanced VR - controlled exposure and relaxation, in order to investigate treatment effects. Mobile phone also used to enable virtual experience in an outpatient setting. Participants randomly assigned to VR and mobile with biofeedback group (VRMB), VR and mobile without biofeedback group (VRM) or waiting list group (WL). | Improvements in anxiety symptoms in both VRMB and VRM groups, however only the VRMB group showed a significant reduction in anxiety scores following treatment, as well a great tendency for HR and GSR to decrease between pre and post treatment (more so than in VRM group). Suggests use of mobile device to deliver treatment may be useful and that using physiological data to modify specific features of a VR environment had a clinical benefit. |
| **61. Harris, S. et al. (2002)** | Social Phobia - Public speaking anxiety | Treatment | 14 | Student volunteers interested in/attending/had previously attended a university public speaking class with a PRCS score of less than 16. | HMD with head tracking - VR audience. Manipulation of audience size. | Investigate the use of VR therapy in the treatment of public speaking anxiety. Four weekly 15 minute exposure treatment sessions, have to give speech to VR audience of varying size. | VR therapy group showed reduction in anxiety and avoidance of public speaking following treatment, greater than in waiting list group. |
| **62. Hartanto, D. et al. (2012)** | Social Phobia | Assessment | 24 | Non-clinical adult volunteers. | 3.5x2.5m projection screen, sat 2m away with a microphone - VR audience. Presentation followed by question and answer session within VR. | Examined timing mechanisms (dialogue dependent, speech dependent, context independent) in scripted avatar-patient dialogues - aiming to develop a home VRET system that uses speech technology for those with social phobia. Throughout VR, participants asked to give SUD score. | Subjective experience of VR environment appeared to be affected by automatic timing of SUD scoring. Dialogue dependent timing mechanisms was superior, followed by speech dependent and context independent timing mechanisms. |
| **63. Hartanto, D. et al. (2014)** | Anxiety - SAD | Assessment | a) 16 b) 24 | Non-clinical adult volunteers | HMD with head tracking. a) 3 VR scenarios - neutral, blind date, job interview. b) VR job interview, systematic variation of ration of positive and negative feedback. | Explored the use of VR therapy for anxiety using 2 stimuli control studies: a) a social dialogue situation, and b) a situation involving dialogue feedback responses between human and virtual character. | a) Significant increase in participants' anxiety level as VR scene changed from neutral to blind date or job interview.  b) Manipulation of dialogue feedback had a significant effect on anxiety level, attitudes, dialogue experiences, speech behaviour, emotions and how virtual character was perceived - suggests possible use in future research as an effective anxiety stressor. |
| **64. Herrmann et al (2016)** | Acrophobia | Treatment | 39 | Individuals with fear of heights | CAVE | Aim was to test whether targeting the ventral medial prefrontal cortex with transcranial magnetic stimulation (rTMS)accelerated extinction learning. | The rTMS enhanced the VR treatment effect. |
| **65. Hirsch, J. et al. (2012)** | Phobia - Flying | Treatment | 1 | 69 year old male with history of flying phobia (spanning 50 years). Refractory to traditional methods of treatment, significant impact on everyday life. | Unclear | Case study - investigated use of VR exposure and hypnosis for the treatment of flying phobia. CBT also incorporated. | Successful reduction of phobia-related anxiety. |
| **66. Hodges, L. et al. (1995)** | Phobia - Acrophobia | Treatment | 17 | Students identified as possible acrophobic. | HMD - 3 VR environments; elevator, series of balconies, series of bridges. | Pilot study using VR graded exposure as a means of treating acrophobia - particular focus on sense of presence and efficacy. | Strong sense of presence in VR environments. Decreases in anxiety, avoidance and negative attitudes towards heights following treatment. |
| **67. Hoffman, H. et al. (2003)** | Phobia - Arachnophobia | Treatment | 36 | Student volunteers scoring 1 SD< class mean in Fear of Spiders Questionnaire given to their class. | HMD with head and wrist tracking - VR spider. | Explored the use of VR in the treatment of arachnophobia using exposure therapy, and whether illusion of physically touching virtual spiders would increase treatment effectiveness. Participants randomly assigned to receive a) no treatment, b) VR with no tactile cues or c) VR with physically 'touchable' spider. | Both VR and VR with tactile augmentation groups showed significantly reduced fear of spiders following three 1-hour sessions. Clinically phobic participants showed marginal statistically and clinically significant reductions in fear following sessions. Those in tactile augmentation were able to approach real spider to a closer distance that VR alone group, and reported stronger sense of realism. |
| **68. Huang, M. et al. (2000)** | Phobia - Acrophobia | Theory | 9 | Adult volunteers meeting criteria for specific phobia. | CAVE - VR building with elevator. | Compared the use of in vivo and VR exposure in the treatment of acrophobia, focusing on how far vivid visualisation was related to the experience of phobia. | Vividness of visualisation was shown to increase fear and arousal scores across participants. |
| **69. James, L. et al. (2003)** | Anxiety - SAD | Assessment | 10 | Non-clinical adult volunteers | Projection, head and hand tracking. 2 VR environments: wine bar, underground train journey. | Explored extent to which social anxiety symptoms can be induced in a VR environment. | Social anxiety induced in VR environments, higher in wine bar experience which was more socially charged than the neutral underground train. |
| **70. Jang, D. et al. (2000)** | Panic Disorder with agoraphobia | Assessment | 7 | Diagnosis of panic disorder with agoraphobia. | HMD with head tracking - VR environments of feared scenes. | Evaluated the effectiveness of VR therapy in the treatment of participants with panic disorder, using subjective and objective tools. | VR environment elicited physical and emotional symptoms of distress, but no further physiological changed seen with continued VR immersion. Unable to evaluate effectiveness of VR as participants were unable to become fully immersed in the VR environment. |
| **71. Jang et al (2002)** | Fear of heights | Treatment | 1 | Patient with acrophobia | HMD | A case study report. | Fear of heights reduced. |
| **72. Johnson, S. et al. (2014)** | Anxiety - SAD | Treatment | 74 | Diagnosis of SAD. | HMD - VR environments with varying numbers of audience members, participant had to give speech. | Secondary analysis of Anderson et al. (2013). Examined whether stereotype confirmation concerns were related to dropout from CBT in individual and group therapy formats. Participants had been randomly assigned to VR exposure therapy (VRE), exposure group therapy (EGT), with 8 sessions in therapy groups. Reactions and number of audience members manipulated. | Stereotype confirmation concerns were shown to be related to dropout rate. Suggests this is due to concerns about negative evaluation of individual's social group and own self as a representative of that group may increase fear of social situations, hence resulting in dropout. |
| **73. Josman, N. et al. (2008)** | PTSD | Assessment | 30 | Non-clinical adult volunteers | HMD with head tracking - VR 'BusWorld' environment. | Explored the use of 'Bus World' - a VRET program designed to facilitate CBT for those experiencing PTSD following a terrorist bus bombing. Experienced four levels of described environment. | As level of VR exposure increased, levels of distress significantly increased - suggest BusWorld is a suitable and effective program for providing graded exposure. |
| **74. Juan, M et al. (2009)** | Phobia - Acrophobia | Assessment | 25 | Non-clinical adult volunteers. | HMD and CAVE - VR environment of floor falling away and walls rising up. | Compared levels of presence and anxiety in non-phobic users in an acrophobia environment - participants counterbalanced and assigned to receive either CAVE or HMD VR conditions first (all participants took part in both). | Both systems induced anxiety in participants, but both higher in the CAVE condition. A high sense of presence was reported in the CAVE condition, but not in HMD. Significant correlation between level of anxiety and level of presence was found. |
| **75. Juan, M. & Pérez, D. (2010)** | Phobia - Acrophobia | Assessment | 20 | Non-clinical adult volunteers. | HMD (additional markers for AR) - VR/AR environment of floor falling away and walls rising up. | Compared levels of presence and anxiety in non-phobic users in an acrophobia environment - participants counterbalanced and assigned to either AR or VR conditions first (all participants took part in both). | No differences observed in anxiety and presence levels in VR versus AR condition - suggests AR may be as effective as VR in treating acrophobia. Significant difference in anxiety level experienced prior to immersion in VR/AR compared to during. Low correlation between level of anxiety and level of presence. |
| **76. Kahan, M. et al. (2000)** | Phobia - Flying | Treatment | 31 | Had completed treatment for fear of flying. Met criteria for specific phobia - fear of crash (CR), claustrophobia (CL), fear of heights (HTS, or panic disorder with agoraphobia (AG). | V6 headset, computer, microphone, receiver - VR flight scenarios. | Explored use of VR assisted CBT as a treatment for fear of flying. Participants gradually exposed to VR flight scenarios. | 21 patients flew following treatment (main outcome measure), suggesting treatment was successful. Follow up revealed that patients continued to fly but with anxiety. |
| **77. Kampmann, I. et al. (2016)** | Anxiety - SAD | Treatment | 60 | Met criteria for SAD. | HMD - VR human characters (varied dialogue style, numbers of, gender, conversational topic's personal relevance, avatar's gestures). | Explored the efficacy of VR exposure therapy involving verbal interaction with virtual humans which aims to target heterogeneous social fears in individuals with SAD. Participants randomly assigned to receive either VRET, in vivo exposure or waitlist. | Improvements seen in social anxiety symptoms, speech duration, perceived stress, and avoidant personality disorder related beliefs in both VRET and in vivo groups following treatment. Improvements in fear of negative evaluation speech performance, general anxiety, depression, and quality of life only seen in in vivo treatment group. Follow up assessments suggested in vivo exposure was more effective, with more improvements maintained. |
| **78. Kim, K. et al. (2008)** | OCD | Assessment | a) 33 b) 30 | a) Diagnosis of OCD b) Non-clinical controls (matched by age, gender, IQ and VR experience) | HMD with head tracking and joystick - VR environment of average Korean home (designed to induce checking impulses). | Explored the efficacy of a VR anxiety-provoking tool, comparing participants with OCD to healthy controls. | Significantly higher anxiety levels in VR environment seen in OCD group compared with non-clinical controls. Degree of anxiety of participants in OCD group correlated with their symptom and immersive tendency scores - overall suggesting VR is useful for provoking anxiety in OCD patients. |
| **79. Kleim, B. et al. (2014)** | Phobia - Arachnophobia | Treatment | 40 | Met criteria for specific phobia. | HMD with head tracking - VR environment of spiders. | Investigated the beneficial effect of sleep on memory consolidation following VRET for the treatment of phobic anxiety. Participants underwent one session of VRET, then either slept for 90 minutes or stayed awake immediately after. | Self-reported fear and catastrophic spider-related cognitions during the approach of a real-world spider following treatment were lower in sleep group (1 week post-treatment). Greater reductions were related to greater stage 2 sleep. Suggests sleep led to greater therapeutic success. |
| **80. Klinger, E. et al. (2005)** | Social Phobia | Treatment | 36 | Met criteria for social phobia, suffering with condition for 2-25 years. | 4 VR environments corresponding to particular case of social anxiety: public audience, apartment, street, retail environment with elevator. | Explored the efficacy of VRET in the treatment of social phobia. Compared against CBT-only control condition. 12 sessions. | Both groups showed reduced social anxiety symptoms following treatment, as well as improved social and global functioning. Other than 'assertiveness' (CBT superior), there was no effect size difference between groups in outcome measures. |
| **81. Krijn, M. et al. (2007)** | Phobia - Acrophobia | Treatment | 26 | Met criteria for specific phobia. | HMD with head tracking - 3 VR environments: fire escape, roof garden, building site (increasing number of floors) | Investigated how far coping self-statements would enhance the effectiveness of VRET in the treatment of acrophobia. Participants randomly assigned to two sessions of VRET followed by two sessions of VRET with coping self-statements, or reverse. | VRET was effective in reducing height anxiety and avoidance behaviours, as well as improving attitudes towards heights with or without coping self-statements. Most treatment gains were not maintained at 6 month follow up. |
| **82. Krijn, M. et al. (2004)** | Phobia - Acrophobia | Treatment | 37 | Met criteria for specific phobia, naturalistic type. | HMD and CAVE - VR environment of floor falling away and walls rising up. 4 VR gradual exposure scenarios: shopping mall, fire escape, roof garden (increasing number of floors). | Manipulated level of presence during VRET using either HMD or CAVE in order to investigate role of immersion and presence in treatment outcomes. 3 weekly 1.5hr sessions over course of 4 weeks. | Both VRET conditions were found to be effective in reducing anxiety and avoidance behaviours, and in improving attitudes towards heights, compared to control group - results maintained at 6 month follow up. No significant differences between effectiveness of HMD and CAVE systems. |
| **83. Krijn, M. et al. (2007)** | Phobia - Flying | Treatment | 86 | Met criteria for specific phobia, situational type (FP). | HMD with head tracking - VR environments of airport and an aircraft. | Compared the effectiveness of bibliotherapy (BIB), VRET and CBT in the treatment of fear of flying. Also explored the effect of following up VRET with CBT. | VRET and CBT was found to be more effective than BIB (though only small to moderate effect size for VRET and moderate for CBT). Additional follow-up group CBT had less of an effect on VRET participants than those in the CBT group. |
| **84. La Paglia, F. et al. (2014)** | OCD | Assessment | a) 30 b) 30 | a) Diagnosis of OCD b) Non-clinical controls (matched for age and gender) | Unclear | Evaluated the reliability and validity of the 'Neuro-Virtual Reality' program used for the neuropsychological assessment of individuals with OCD. | Program was able to detect cognitive difficulties typical of CBT suggesting it is a valid means of evaluating executive functioning in patients with OCD. |
| **85. La Paglia, F. et al. (2012)** | OCD | Assessment | a) 10 b) 10 | a) Diagnosis of OCD b) Non-clinical controls (matched for age and gender) | Unclear | Evaluated the executive functioning of patients with OCD using VR multiple errands task. | OCD performed worse than the controls, with VR task performance correlating with other neuropsychological measures of performance. Suggests it is an ecologically valid means of evaluating executive functioning in OCD. |
| **86. Laforest, M. et al. (2016)** | OCD | Treatment | 3 | Diagnosis of OCD with primary subtype of contamination. | CAVE - VR environment of 'neutral' training scene and 'contaminated' experimental scene of public bathroom. | Investigated the use of VR exposure CBT in the treatment of OCD. | OCD symptoms of all patients improved following treatment, with global improvement seen in 2 participants. Gains maintained up to 4 months following treatment. |
| **87. Levy et al (2016)** | Fear of heights | Treatment | 6 | Individuals with acrophobia | HMD | Six sessions of VR exposure therapy, three in presence of therapist. | Patients were able to do the exposure on their own. |
| **88. Levy, F. et al. (2016)** | Phobia - Falling | Treatment | 16 | Elderly individuals with diagnosis of specific phobia: disproportionate fear provoked by walking. | 2x1.5m screen with movement tracking - VR video games of washing foam-covered window and kung fu. | Explored the use of VR associated with serious games in the treatment of fear of falling. 12 weekly sessions. | Reductions in fear of falling in VR group following treatment, suggesting it is an effective treatment option. |
| **89. Malbos, E. et al. (2008)** | Phobia - Claustrophobia | Treatment | 6 | Met criteria for claustrophobia. 3 with co-morbid phobias (acrophobia, driving phobia or agoraphobia) | HMD with head tracking - nine VR environments suitable for claustrophobia. | Explored the use of multiple-components therapy using VR in the treatment of claustrophobia. First three sessions of treatment involved different therapeutic components, with VR exposure in following five sessions. | Fear of enclosed space was significantly reduced and quality of life improved in all participants following treatment. Gains maintained at 6 month follow up. Co-morbid patients also experienced improvements in other situations - eg. Height or crowded spaces, demonstrating a more global effect. |
| **90. Malbos, E. et al. (2013)** | Panic Disorder with agoraphobia | Treatment | 18 | Met criteria for panic disorder with agoraphobia. | HMD with head tracking, steering wheel - VR environments of anxiety-inducing places for individuals with agoraphobia. | Evaluated the independent effectiveness of VRET in the treatment of agoraphobia. Participants either underwent VRET alone or VRET with CBT. | Both groups showed reduced anxiety and overall improvement following treatment, with treatment outcome being positively predicted by presence. No additional benefits observed in the VR + CBT group, suggesting isolated effects of VRET is sufficient for positive treatment outcome. Improvements maintained at 3 month follow up. |
| **91. Maltby, N. et al. (2002)** | Phobia - Flying | Treatment | 45 | Met criteria for specific phobia, situational type; agoraphobia; or panic disorder with agoraphobia, where flying is the primary feared stimulus. Refused to fly during a pretreatment screening test. | HMD with joystick. VR graded exposure to flying (airport, airplane, runway etc.) | Investigated the effects of VRET compared to attention-placebo treatment in patients with fear of flying. | Significant improvements and reduced flight anxiety in both groups following treatment - 65% VRET participants and 57% of GT able complete test flight afterwards. Better outcome of VRET participants on 4 of 5 self-report anxiety measures. Group differences no longer evident at 6 month follow up. |
| **92. McLay, R. et al. (2012)** | PTSD | Treatment | 20 | Active duty soldiers/marines with diagnosis of chronic PTSD related to combat operations in Iraq or Afghanistan. | Unclear | Developed and evaluated a VRET method for active duty service members experiencing PTSD. | Significant reduction in PTSD symptoms, anxiety and depression levels post-treatment, with 75% participants no longer meeting criteria for PTSD diagnosis. Improvements maintained at 3 month follow up. |
| **93. McLay, R. et al. (2011)** | PTSD | Treatment | 20 | Active duty service members with diagnosis of PTSD related to service in Iraq or Afghanistan. | HMD with joystick - VR simulation of Iraq or Afghanistan. | Investigated the use of VR graded exposure therapy as compared to treatment as usual (TAU) for active duty members with PTSD. | Significant improvements in PTSD symptoms of 7/10 VR group participants, with significant improvements in only 1 TAU group participant. |
| **94. McLay, R. et al. (2014)** | PTSD | Treatment | 15 | Active duty service members with diagnosis of PTSD related to service in Iraq or Afghanistan. | VR goggles - VR simulation of Iraq or Afghanistan. | Investigated the changes in self-reported measures of PTSD, depression and anxiety as well as neuropsychological functioning before and after VR graded exposure therapy. Used participants from two previous trials. | Treatment resulted in significant reductions in PTSD and anxiety severity, alongside significant improvements on the emotional stroop test. Improvements in depression and other neuropsychological function measures not observed - suggests narrowly focused benefits of VR therapy. |
| **95. Meyerbroeker et al (2013)** | Panic disorder with agoraphobia | Treatment | 55 | Patients with panic disorder with  agoraphobia | CAVE or HMD | Randomly allocated to four sessions of CBT followed by either 6 sessions of VRET or 6 sessions of exposure in vivo or to a waiting list control condition | The active treatments were generally comparable (for three out of four outcome measures). |
| **96. Meyerbroeker, K. et al. (2012)** | Phobia - Flying | Treatment | 45 | Met criteria for specific phobia, situational type - fear of flying. | VR glasses with head tracking - VR aircraft environment. | Examined whether administration of yohimbine hydrochloride (YOH), a noradrenaline agonist enhanced exposure treatment by facilitating fear extinction. Received either 10mg of YOH or placebo capsule followed by 4 VRET sessions - capsules administered 1hr prior to each session. | Improvement in flight anxiety in all participants following treatment. No exposure-enhancing effects of YOH observed. |
| **97. Miyahira et al (2010)** | PTSD | Treatment | 1 | Soldier with PTSD | HMD | A report of a case study. | There was an improvement in PTSD scores. |
| **98. Miyahira et al (2012)** | PTSD | Treatment | 42 | Soldiers with PTSD | HMD | Randomised to VR exposure treatment or control condition. | No account was made for missing data. There were some benefits for the VR treatment. |
| **99. Michaliszyn, D. et al. (2010)** | Phobia - Arachnophobia | Treatment | 43 | Met criteria for specific phobia, animal type - spiders, with minimum 1 year duration of phobia. | HMD with head tracking - VR environment of spiders (varying shapes and sizes). | Explored the efficacy of VRET compared with in vivo exposure in the treatment of spider phobia. Eight 1.5hr sessions. | Both groups displayed clinically and statistically significant improvements following treatment. Group differences on only 1/5 outcome measures of fear - greater improvement in in vivo group in SBQ-F beliefs subscale. |
| **100. Moore, K. et al. (2002)** | Panic Disorder with agoraphobia | Assessment | 9 | Non-clinical adult volunteers. | HMD with head tracking, joystick - 6 VR environments of social settings. | Explored how nonphobic individuals respond to VR environments created for the treatment of panic disorder and agoraphobia, with aim of establishing a baseline for physiological responses. | Participants became sympathetically aroused when exposed to nonthreatening VR environments - arousal levels tended normalized towards baseline. Useful for exploring differences in immersion and responses between phobic and non-clinical patients. |
| **101. Morina, N. et al. (2014)** | Anxiety | Assessment | 43 | Non-clinical adult volunteers | HMD or on-screen projection display (1.9x1.45m) - VR social situations. | Investigated how far different VR displays elicited different levels of presence and anxiety whilst participants interacted with virtual humans. | VR environment elicited moderate levels of presence and anxiety in all participants. HMD elicited significantly higher levels of presence, but no differences in anxiety reported. |
| **102. Morina, N. et al. (2015)** | Anxiety | Treatment | 43 | Non-clinical adult volunteers. Either with high levels of social anxiety (n = 16 at follow-up) or low level (n = 18 at follow up). | HMD or on-screen projection display (1.9x1.45m) - VR social situations with semi-scripted dialogues. | Explored the integration of interaction between participants and virtual humans in VRET used for anxiety disorders. 2 VRET sessions - free speech dialogues with virtual humans whilst being monitored by a therapist. | Participants with high levels of social anxiety reported significantly lower levels of social anxiety at 3-month follow up post-treatment. No significant changes reported in low anxiety group. Both groups reported higher self efficacy following treatment. |
| **103. Mosig, C. et al. (2014)** | Phobia - Arachnophobia | Theory | a) 25 b) 18 | a) Spider-fearful b) Non-fearful | HMD - VR conditioning paradigm; various contexts, with mild electrical stimulation applied in some. | Compared phobic and non-phobic individuals in order to explore changes in fear acquisition and extinction during conditioning paradigm using VR. | Spider-fearful individuals displayed enhanced aversive discrimination learning for de novo stimuli (shown in level of electrodermal responses), alongside more negative evaluation of the CS+ as compared to the CS− throughout conditioning procedure (acquisition, extinction, and fear retrieval phases). No specific difference in extinction learning found between groups. |
| **104. Mühlberger, A. et al. (2001)** | Phobia - Flying | Treatment | 30 | Met criteria for simple phobia - flying, strong motivation to reduce fear. | HMD with head tracking, motion base - VR airplane. | Examined the effect of repeated VR flight exposure in the treatment of flying phobia. Participants all received a VR test flight, followed by either 4 VR exposure sessions of a long relaxation training session - all completed final VR test flight following treatment. | Fear of flying reduced in both groups - Greater fear reduction as a result of repeated VR exposure as compared to relaxation group. |
| **105. Mühlberger, A. et al. (2007)** | Phobia - tunnel fear | Assessment | a) 15 b) 15 | a) Highly tunnel-fearful individuals (TAF score of 1.5<) b) Non-clinical controls (age and gender matched) | Curved 7m projection screen - VR road environments; open, tunnel, gallery. | Assessed the psychophysiological reactions of fearful and non-fearful individuals in VR tunnel simulation. | Elevated fear responses in tunnel-fearful group- HR and fear ratings able to differentiate fearful and control participants with respective accuracies of 88% and 93%. Suggest VR is suitable and valuable tool for assessment of fear reactions. |
| **106. Mühlberger, A. et al. (2006)** | Phobia - Flying | Treatment | 30 | Met criteria for specific phobia - flying | HMD with head tracking, motion base - VR airplane. | Further evaluation of Mühlberger et al. (2003) - investigation into the efficacy of brief 1-session VRET in treatment of fear of flying. Evaluated treatment effects on flight taken post-treatment (either with or without a therapist) and long-term efficacy. | Significant fear of flying reductions following treatment, maintained at 12-month follow up. 87% of accompanied, and 67% of alone travellers completed graduation flight - not a significant difference. Completion of graduation flight was strong predictor of long term efficacy. |
| **107. Mühlberger, A. et al. (2010)** | Phobia - Flying | Treatment | 47 | Met criteria for simple phobia - flying. | HMD with head tracking, motion base - VR airplane. | Examined the efficacy of brief 1-session VRET in treatment of fear of flying - aimed to establish whether VR exposure is the key active treatment component. Participants randomly assigned and received either cognitive treatment alone (COG), VR exposure accompanied with motion simulation (VR-mot) or unaccompanied VR exposure (VR-no-mot). | Reduced fear of flying observed only in VR exposure groups, maintained at 6-month follow up. Similar treatment outcomes in both VR groups, suggesting motion simulation does not enhance treatment effects. Visual and acoustic stimuli were found to be main active components of VRET. |
| **108. Mühlberger et al (2012)** | Fear of heights | Theory | 56 | Volunteers | HMD | Appraisals and depth were manipulated. | Both beliefs and depth cues affect height fears. |
| **109. Ngai, I. et al. (2015)** | Anxiety - SAD | Treatment | 63 | Met criteria for social phobia, with fear of public speaking as the predominant social fear. | HMD - VR environments with varying numbers of audience members. Manipulated according to the participants’ fear hierarchy | Secondary analysis of Anderson et al. 2005) - Investigated the trajectory of the working alliance in 2 types of CBT for the treatment of SAD – VRET) and exposure group therapy (EGT). Participants randomized to a therapy and received 8 treatment sessions. | Increases in working alliance scores were steeper at start of therapy, slowing towards the end. No differences in working alliance between groups. |
| **110. Norrholm, S. et al. (2016)** | PTSD | Assessment | 50 | Met criteria for PTSD with index trauma(s) related to combat experience in Iraq and/or Afghanistan. | HMD - VR scenes of war environment; gun turret, Humvee cabin, Iraqi city. | Examined how far initial psychophysical and stress-hormone responses were predictive of VR PTSD treatment outcome. Administered D-cycloserine (DCS), alprazolam (ALP), or placebo (PBO) to participants in order to observe effects. Startle probes delivered during VR scenes. | In DCS group higher startle responses predicted greater changes in symptom severity over time (up to 6-month follow up). In ALP group higher baseline cortisol reactivity was associated with lower treatment response, however baseline cortisol levels was associated with the opposite (greater treatment response). Suggests that baseline cue-activated measures are useful and sensitive as predictors of treatment response. |
| **111. Notzon, S. et al. (2015)** | Phobia - Arachnophobia | Treatment | a) 41 *21 = iTBS, 20 = sham* b) 42 *19 = iTBS, 23 = sham* | a) Met criteria for specific spider phobia  b) Non-clinical controls | HMD with head tracking - VR neutral and spider environments. | Explored the effects on anxiety of intermittent theta burst stimulation (iTBS) - a form of TMS - applied to PFC prior to VR experience. | Phobia group reacted with more anxiety compared to healthy controls. iTBS did not influence reactions, but did modulate heart rate variability (HRV) - increase in sympathetic influence on HRV seen in active iTBS group only. |
| **112. North & Rives (2003)** | Fear of flying | Treatment | 1 | Person with acrophobia | HMD and ‘rumble-chair’. | A case study report. | There were indications the fear of flying had lessened. |
| **113. Pallavicini, F. et al. (2013)** | Anxiety | Assessment | 39 | Non-clinical adult volunteers | HMD with joystick - VR simulation of academic oral examination in classroom. | Explored how far the technological breakdowns of VR may be risk factors for its effectiveness. Participants were exposed to an academic examination using text (TX), audio (AU), video (VD) and VR (which included technological breakdowns) - counterbalanced orders. | All conditions resulted in increased anxiety and decreased relaxation scores - all able to induce negative emotional response. VR was shown to be less effective in eliciting stress responses than other conditions. Similar levels of presence across conditions. Suggests VR requires a high sense of presence in order to achieve superior emotional induction abilities. |
| **114. Pan, X. et al. (2012)** | Anxiety - SAD | Assessment | a) 18 b) 18 | a) Adult volunteers with high confidence score on SPAI.  b) Adult volunteers with low confidence score on SPAI. | CAVE - VR party with 5 virtual characters: 4 sitting at a distance and 1 lone woman who initiates a conversation. | Investigated differences in how socially anxious and socially confident men interacted with a virtual woman. | Initial approach of virtual woman increased stress in both groups, diminishing once conversation entered mundane phase. Anxiety scores remained the same for confident participants pre- and post-testing, but significantly decreased amongst the anxious group. |
| **115. Peñate Castro, W. et al. (2014)** | Agoraphobia | Treatment | 50 | Met criteria for agoraphobia (with or without panic disorder). | 2.5x2m screen, 3D glasses - 7 VR environments reflecting possibly phobic stimuli for those with agoraphobia. | Compared the efficacy of VR exposure combined with CBT (VRET) to traditional CBT alone and medication only in the treatment of long-term agoraphobia. 11 sessions. | All groups showed significant improvements, maintained at 6-month follow up. The VRET group showed clinical improvement in most variables measured at follow-up. The CBT group showed the highest dropout rates. |
| **116.Peñate, W. et al. (2008)** | Agoraphobia | Treatment | 28 | Met criteria for agoraphobia (with or without panic disorder). | 2.5x2m screen, 3D glasses - 7 VR environments reflecting possibly phobic stimuli for those with agoraphobia. | Compared the efficacy of VR exposure combined with CBT (VRET) to traditional CBT alone in the treatment of agoraphobia. 11 sessions. | Significant improvements in agoraphobia symptoms seen in both groups post-treatment. Largely maintained at 3-month follow up. Analysis revealed similar or better improvements in VRET group. |
| **117. Peperkorn, H. et al. (2013)** | Phobia - Arachnophobia | Theory | a) 48 b) 48 | a) Met criteria for specific phobia - spiders. b) Non-clinical controls (age and gender matched). | HMD - VR spider. | Investigated the effect of specific perceptual cues and conceptual fear-related information on fear responses. Manipulated available cues and information in VR environment. | Phobic patients reported higher levels of in conditions with a fear-relevant perceptual cue, rather than when only information was presented. Skin conductance was significantly higher in combined condition, compared with cue or information only. Suggests perceptual cues are essential for phobic fear reactions, producing intense reactions when combined with fear-relevant information. |
| **118. Peperkorn, H. et al. (2016)** | Phobia - Arachnophobia | Assessment | a) 32 b) 32 | a) Females meeting criteria for specific phobia - spiders b) Matched non-clinical controls. | HMD - Received fear-relevant visual input (VR spider) with information about unseen neutral animal (snake), or neutral control animal input (VR snake) with information about fear-relevant animal (spider). | Explored the influence of presentation of the participant's hand (embodiment) during VR exposure for spider phobia. Along with VR input, all participants presented with real acrylic glass container (matched in VR, containing animal), instructed to touch it - visibility varied randomly. | Visibility of hand was shown to increase presence in VR, independent of fear trigger. Fear trigger influenced the impact of the virtual hand - when fear was perceptually triggered, the VR hand increased fear, but no effect when fear was triggered by information. |
| **119. Pertaub, D. et al. (2002)** | Phobia - Acrophobia | Assessment | 40 | Non-clinical adult volunteers. | HMD with head tracking or computer screen - VR seminar room environment with eight male avatars. | Explored anxiety responses of participants when giving presentation to a VR audience, the behaviour of which was manipulated (neutral, positive or negative participant condition groups). Also explored impact of level of immersion by comparing HMD and computer screen presentation. | In neutral and positive VR audience conditions, post-treatment confidence in public speaking positively correlated with pre-test levels, but was not a predictor in negative VR condition. Negative VR audience elicited greatest anxiety response regardless of pre-test public speaking confidence level. Greater immersion in HMD condition, particularly for female participants. |
| **120. Piercey, C. et al. (2012)** | Phobia - Arachnophobia | Treatment | 6 | Volunteers with significant fear of spiders (score of 80 or above on FSQ). | Computer screen with red/blue anaglyph glasses - VR spider environments. | Investigated the efficacy self-help VR software using anaglyph glasses in the treatment of spider phobia. No therapist present during software - 4 sessions, focus on exposure therapy and muscle relaxation. | Fear responses and self-reported fear ratings reduced following treatment, suggesting it is a possible alternative to more expensive VR systems. |
| **121. Powers, M. et al. (2013)** | Anxiety - SAD | Assessment | 26 | Undergraduate volunteers. | HMD with head tracking - VR avatar in same position as in vivo. | Investigated whether conversing with VR was able to induce feelings of social anxiety, thus having potential applications for social anxiety treatment in the future. All participants experienced conversation in VR and in vivo. | VR conversations resulted in elevated fear levels, with participants rating fear higher in VR than in vivo. In vivo condition was rated as more realistic. |
| **122. Price, M. & Anderson, P. (2007)** | Phobia - Flying | Assessment | 36 | Met criteria for either specific phobia, situational type (n = 31) or panic disorder with agoraphobia (n = 5) with flying the predominantly feared stimulus. | HMD - VR aircraft scenarios. | Investigated the influence of presence in VRET for flying phobia - focusing on in-session anxiety, mediation of pre-test anxiety during exposure, relation to phobic elements in VR environment and treatment outcome. 8 sessions across 6 weeks. | Feeling of presence strongly correlated with in-session anxiety. Participants with greater phobic anxiety often experienced more presence. Presence also found to strongly mediate relationship between pre-session and in-session anxiety levels, but no relationship to treatment outcome - suggests presence may be necessary in VRET, but not sufficient. |
| **123. Price, M. & Anderson, P. (2012)** | Anxiety - SAD | Treatment | 67 | Diagnosis of SAD (n = 33 met criteria for generalised subtype). | HMD with head tracking - VR audience. | Explored how far outcome expectancy may be a predictor of treatment response for public-speaking fears. Investigated by randomising participants to either VRET or group-based CBT (EGT) program and measuring expectancy - some underwent 8 week waiting list period before being assigned. | In both groups, early outcome expectancy was found to be related to post-treatment variance in public speaking fears - no difference in effect between groups. |
| **124. Price, M. & Anderson, P. (2011)** | Anxiety - SAD | Treatment | 91 | Diagnosis of SAD (n = 46 met criteria for generalised subtype). 22 with co-morbid diagnoses. | HMD with head tracking - VR audience. | Explored how far post event processing (PEP) decreased as a result of treatment and whether it limits treatment response. Investigated by randomising participants to either VRET or group-based CBT (EGT) program. | Following treatment, PEP reduced. In participants with higher levels of PEP, social anxiety symptoms improved at a slower rate than those with lower PEP levels - suggests PEP attenuates treatment response. |
| **125. Price, M. et al. (2008)** | Phobia - Flying | Treatment | 72 | Met criteria for either specific phobia, situational type (n = 53), panic disorder with agoraphobia (n = 8) with primary fear or flying, or agoraphobia without panic disorder (n = 1). | HMD - VR aircraft scenarios. | Explored how far expectancy about treatment outcome relates to actual treatment outcome. Investigated using participants being treated for fear of flying using VRET or in vivo exposure. | Treatment outcome expectancy largely positively correlated with treatment outcome, but was not maintained at 12-month follow up. |
| **126. Price, M. et al. (2015)** | PTSD | Treatment | 116 | Met criteria for PTSD due to military trauma. | HMD - VR Humvee caravan, cues related to specific memory of given participant. | Explored how far expectancy about treatment outcome relates to actual treatment outcome. Investigated using participants being treated for PTSD using VRET - unlike other studies, clinician-rated measures and biological indices used alongside self-report measures.  *Experimental medications (D-Cycloserine 50mg or alprazolam 0.25mg, compared to placebo) administered 30 minutes prior to each exposure session.* | Outcome expectancy found to be related to self-reported magnitude of change during treatment. Negative relationship between outcome expectancy and symptoms (self- and clinician-reported). No relationship to biological measures of treatment response. |
| **127. Price, M. et al. (2011)** | Social Phobia | Assessment | 41 | Met criteria for social phobia | HMD with head tracking - VR audience. | Explored the relationship between three theorised components of presence, ratings of fear and treatment outcome - VRET for treatment of social phobia. | Presence was found to relate to in-session peak fear ratings, but treatment response was only predicted by involvement subscale scores. |
| **128. Ready et al (2010)** | PTSD | Treatment | 11 | Veterans with PTSD | HMD | VR exposure therapy compared to present-centred therapy | This study was underpowered but showed potential benefits of the VR treatment. |
| **129. Ready et al (2006)** | PTSD | Treatment | 21 | Veterans with PTSD | HMD | This was an open trial. | In this uncontrolled work, PTSD decreased. |
| **130. Reger & Gahm (2008)** | PTSD | Treatment | 1 | Soldier with PTSD | HMD | A case study, testing six 90minute sessions. VR exposure lasted about 35 minutes on average. | The patient had reductions in PTSD. |
| **131. Reger et al (2009)** | PTSD | Assessment | 93 | Soldiers without PTSD | HMD | The soldiers rated the realism of a virtual Iraq | Realism was rated well. |
| **132. Reger et al (2011)** | PTSD | Treatment | 21 | Soldiers with PTSD | HMD | Open case series | There were reductions in PTSD. |
| 133. Reger et al (2016) | PTSD | Treatment | 162 | Soldiers with PTSD | HMD | Randomized to 10-sessions of prolonged imaginal exposure, or VR prolonged exposure, or a minimal attention waitlist. | Both treatment groups had high drop-out rates. Prolonged exposure was better than VR prolonged exposure, which was better than the control condition. |
| **134. Renaud et al (2002)** | Spider phobia | Theory | 24 | Half of the participants had a fear of spiders | HMD | Examined tracking of a virtual spider and a neutral stimulus. | Anxiety was associated with a disruption in tracking. |
| **135. Ressler et al (2004)** | Acrophobia | Treatment | 28 | Patients with acrophobia | HMD | Tested whether D-cycloserine (DCS) improved extinction. | DCS improved outcomes. |
| **136. Rinck et al (2015)** | Spider phobia | Theory | 21 in study 1  42 in study 2 | Study 1: unselected students  Study 2: students screened high or low for fear of spiders | HMD | The studies tested time course of avoidance learning in relation to spiders. | Avoidance learning occurred and was related to level of fear of spiders. Shows potential to use VR to learn more about spider fears. |
| **137. Rizzo et al (2009)** | PTSD | Treatment | 20 | Veterans with PTSD | HMD | Case series of a ten session VR treatment | In this uncontrolled trial there were reductions in PTSD. |
| **138. Robillard et al (2011)** | Social anxiety | Treatment | 45 | Social anxiety diagnosis | HMD | Randomisation to 16 sessions CBT or CBT with VR or wait list. | Both active treatments superior to wait list. |
| **139. Robillard, G et al (2013)** | Specific phobia | Assessment | 26 | Half of the participants has a specific phobia | HMD | Tested the use of cheaper VR programming. | The software produced anxiety as predicted. |
| **140. Rothbaum et al (2002)** | PTSD | Treatment | 10 | Veterans with PTSD | HMD | An open trial. | PTSD symptoms reduced. |
| **141. Rothbaum et al (1999)** | PTSD | Treatment | 1 | Veteran with PTSD | HMD | A case study. | There was a reduction in PTSD. |
| **142. Rothbaum et al (2000)** | Fear of flying | Treatment | 49 | Patients with acrophobia | HMD | Random allocation to VR exposure therapy vs standard exposure vs waitlist. | The active treatments were of benefit in reducing acrophobia. |
| **143. Rothbaum et al (1996)** | Fear of flying | Treatment | 1 | Patient with acrophobia | HMD | A case report. | There was a reduction in fear of flying. |
| **144. Rothabum et al (2006)** | Fear of flying | Treatment | 83 | Patients with acrophobia | HMD | Random allocation to VR exposure or exposure therapy or waiting list. | The active treatments were beneficial. |
| **145. Rothbaum et al (2002)** | Acrophobia | Treatment | 49 | Patients with acrophobia | HMD | 12 month follow-up data from Rothbaum et al (2000) | VR treatment effects were maintained. |
| **146. Rothbaum et al (2014)** | PTSD | Treatment | 156 | Veterans with PTSD | HMD | Tested the effectiveness of a 6 session virtual reality exposure treatment augmented with d-cycloserine or alprazolam, compared with placebo. | D-cycloserine did not improve outcome, alprazolam worsened exposure outcome. |
| **147. Roy et al (2010)** | PTSD | Treatment | 8 | Soldiers with PTSD | Unclear | fMRI scans before and after randomisation to VR exposure therapy or prolonged exposure. Study is on-going according to the paper. | The scanning is finding changes in those treated. But the study is on-going. |
| **148. Roy et al (2003)** | Social phobia | Treatment | 10 | Patients with social phobia | Unclear | VR vs group CBT vs waiting list | The VR treatment looked like it was working in this preliminary report of the trial. |
| **149. Rus-Calafell et al (2013)** | Fear of flying | Treatment | 15 | Patients with fear of flying | HMD | Randomised to imaginal exposure or VR exposure therapy. | Both treatments showed efficacy. |
| **150. Safir et al (2012)** | Public speaking anxiety | Treatment | 88 | Public speaking anxiety | HMD | One year follow up on trial (Wallach et al, 2009) randomised to CBT or VRCBT or wait list. | Both active treatments showed benefits. |
| **151. Wallach et al (2009)** | Public speaking | Treatment | 88 | Public speaking anxiety | HMD | Trial design as above. | As above. The analysis did not seem to be intention to treat. |
| **152. Seinfeld et al (2016)** | Fear of heights | Treatment | 40 | Students | CAVE | Quasi-randomisation to a VR height with or without relaxing music. | Music was associated with lower fear in VR, but it was not tested whether this transferred to the real world (i.e. whether feeling less anxious in VR helps in treatment). |
| **153. Shiban et al (2016)** | Spider fears | Treatment | 32 | Spider phobia diagnosis | HMD | Half of the patients had a fear reactivation procedure before VR exposure. | There was no return of fear in either group. The study was small to detect effects. |
| **154. Shiban et al (2016)** | Spider phobia | Theory | 61 | 41 people with spider phobia, 20 non-clinical controls | HMD | Looked at spider size estimation before and after treatment. | Size estimation bias was reduced in treatment. |
| **155. Shiban et al (2016)** | Claustrophobia | Theory | 96 | 48 claustrophobic patients; 48 non-clinical controls | HMD | Three different VR exposure conditions used. | Visual cues were more important in eliciting fear than conceptual information. |
| **156. Shiban et al (2015)** | Spider phobia | Treatment | 58 | Spider-phobic patients | HMD | Random allocation to: multiple stimuli/single context, multiple contexts/multiple stimuli, multiple stimuli/single context or single stimuli/single context. | Multiple stimuli rather than contexts led to greater treatment improvement. This is an interesting study but small numbers in each condition. |
| **157. Shiban et al (2015)** | Social anxiety | Theory | 40 | Non-clinical volunteers | HMD | Tested translating a social fear conditioning model. | Showed a potential experimental paradigm for fear learning. |
| **158. Simeono et al (2005)** | Height fears | Assessment | 24 | Non-clinical volunteers. | Surround screen VR | Compared real and virtual heights | The VR system induced fear. |
| **159. Slater et al (1999)** | Public speaking anxiety | Assessment | 10 | Non-clinical volunteers | HMD | The researchers examined reactions by factors such as audience reaction and immersion. | VR can be plausibly used to test the treatment of public speaking fears. |
| **160. St-Jacques et al (2009)** | Spider phobia | Treatment | 31 | 8 to 15 year olds with arachnophobia | HMD | Randomised to in vivo exposure with or without VR exposures | In this small study, both treatments were effective. |
| **161. Suied et al (2013)** | Fear of dogs | Assessment | 10 | People with cynophobia | Projection screens and stereoscopic viewing glasses | An initial evaluation of an auditory-visual VR environment. | VR could be used to treat fear of dogs and sounds may be an important element for presence. |
| **162. Taffou et al (2012)** | Fear of dogs | Assessment | 11 | People with fear of dogs | Projection screens and stereoscopic viewing glasses | An initial evaluation of an auditory-visual VR environment. | Fears were provoked by the VR set-up. |
| **163. Taffou et al (2013)** | Fear of dogs | Assessment | 21 | 11 with fear of dogs, 10 with no fear of dogs. | Projection screens and stereoscopic viewing glasses | In VR encountered sounds or images of dogs or both. | Most fear was reported in response to sound and images. |
| **164. Tart et al (2013)** | Acrophobia | Treatment | 29 | Patients with acrophobia. | HMD | VR exposure was conducted with d-cycloserine or placebo. | DCS did not improve outcomes. |
| **165. Tortella-Feliu et al (2011)** | Fear of flying | Treatment | 60 | Flying phobia. | HMD | Randomised to: virtual reality exposure therapy, computer-aided exposure with a therapist’s assistance throughout exposure sessions, and self-administered computer-aided exposure . | All three forms of treatment showed efficacy. Therapist input can be minimised. |
| **166. Triscari et al (2015)** | Fear of flying | Treatment | 65 | People with flight phobia | Unclear | Randomised to: cognitive behavioral therapy (CBT) integrated with systematic desensitization, CBT combined with eye movement desensitization and reprocessing therapy, and CBT combined with virtual reality exposure therapy. | All treatments were beneficial |
| **167. Tworus et al (2010)** | PTSD | Treatment | 1 | A soldier with PTSD | Unclear | A case study. | The person’s PTSD reduced. |
| **168. Urech et al (2015)** | Social anxiety | Treatment | 15 | Students reporting social anxiety | HMD | The VR was designed to shift attention away from threat stimuli – a form of attention bias training. | This uncontrolled feasibility study showed some potential benefits. |
| **169. Vermetten et al (2013)** | PTSD | Treatment | 2 | Two veterans with PTSD | CAREN facility | This was a case series test. | There were reductions in PTSD. |
| **170. Viaud-Delmon et al (2000)** | Anxiety | Theory | 24 | High or low trait anxiety | HMD | Experimental test of response to conflicting visual-vestibular stimulation. | Highlights potential visual dependence in anxiety. |
| **171. Viaud-Delmon et al (2006)** | Agoraphobia | Assessment | 19 | 10 patients with agoraphobia, 9 non-clinical controls | HMD | Testing visual and auditory presentations in VR. | Presence was higher in auditory-visual VR |
| **172. Villa Martin et al (2007)** | Panic disorder with agoraphobia | Treatment | 1 | Patient with panic disorder and agoraphobia | HMD | A case study | There were reductions in symptoms. |
| **173. Vincelli et al (2003)** | Panic disorder with agoraphobia | Treatment | 12 | Panic disorder with agoraphobia | HMD | CBT with VR, CBT, or wait list design. | The active treatments showed benefits. |
| **174. Wald & Taylor (2000)** | Driving phobia | Treatment | 1 | Specific phobia concerning driving | HMD | A case study test. | The person showed improvements. |
| **175. Wald & Taylor (2003)** | Driving phobia | Treatment | 6 | Driving phobia | HMD | A case series. | Improvements were apparent. |
| **176. Wallach & Bar-Zvi (2007).** | Flight phobia | Treatment | 4 | Flight phobia | Unclear | A case series | The patients showed benefits |
| **177. Wallach et al (2009)** | Public speaking anxiety | Treatment | 88 | Public speaking phobia | HMD | Random allocation to VR CBT or CBT or wait list. | Active treatments were beneficial. |
| **178. Wallach et al (2011)** | Public speaking anxiety | Treatment | 20 | Public speaking anxiety | HMD | Random allocation to VR exposure therapy or cognitive therapy | Both treatments were beneficial. |
| **179. Walshe et al (2003)** | Driving phobia | Treatment | 14 | Specific phobia or PTSD related to driving | HMD | VR compared to a computer driving game for immersion, and if shown (n=7) then had a CBT treatment. | Indicates that both forms of car driving presentation may have use. |
| **180. Webb et al (2015)** | PTSD | Assessment | 58 | Veterans with PTSD or without PTSD | HMD | Physiological reactions measured to VR stimuli. | Preliminary study indicating that VR could be used in diagnosis. |
| **181. Whitney et al (2005)** | Fear of heights | Treatment | 1 | Fear of heights and agoraphobia | HMD | Case study of VR exposure and physical vestibular therapy | The patient showed improvement. |
| **182. Wiederhold et al (2002)** | Fear of flying | Treatment | 30 | Fear of flying | HMD | VR exposure therapy, VRET with physiological feedback, or imaginal ET. | The VR treatments were beneficial. |
| **183. Wiederhold et al (2002)** | Fear of flying | Assessment | 58 | 36 people with acrophobia, and 22 non-clinical controls | HMD | Examined physiological responses across groups and VR treatment. | Physiological data from VR may inform understanding and treatment |
| **184. Wiederhold & Wiederhold (2003)** | Fear of flying | Treatment | 30 | Fear of flying | HMD | This was a three year follow up on those reported in Wiederhold et al (2002) | VR treatment gains were maintained. |
| **185. Wieser et al (2010)** | Social anxiety | Theory | 39 | 20 low and 19 high anxiety women | HMD | Aimed to examined the relationship between gaze direction, sex, interpersonal distance, and social anxiety using VR social interactions. | VR can be used to study avoidance behaviours in social anxiety. |
| **186. Wilhelm et al (2005)** | Fear of heights | Theory | 20 | 11 high height anxious, 9 low height anxious students | HMD | Assessed physiological reactions to VR heights to distinguish whether behavioural activation or inhibition systems activated. | The researchers conclude that the behavioural inhibition system is activated in exposure. |
| **187. Won et al (2016)** | Social anxiety | Assessment | 57 | Students | HMD | Head movements in relation to anxiety were tested. | There were differences in head movements by levels of anxiety. |
| **188. Wood et al (2008)** | PTSD | Treatment | 6 | Navy personnel with PTSD | HMD | A case series | There were reductions in PTSD. |
| **189. Wood et al (2007)** | PTSD | Treatment | 1 | A person in the navy with PTSD. | HMD | A case report | There were PTSD reductions. |
| **190. Wood et al (2009)** | PTSD | Treatment | 12 | Combat-related PTSD | HMD | A case series. | There were reductions in PTSD. There were also large cost savings. |
| **191. Wood et al (2009)** | PTSD | Treatment | 1 | Combat-related PTSD | HMD | A case study. | There were PTSD reductions. |
| **192. Wrzesien et al (2013)** | Small-animal (cockroach and spider) phobias | Treatment | 26 | High anxiety to these animals but not diagnosable phobias | Augmented reality. | A case series | Fears about the animals decreased in this uncontrolled study. |

**REFERENCES**

Åhs, F., Kragel, P. A., Zielinski, D. J., Brady, R., & LaBar, K. S. (2015). Medial prefrontal pathways for the contextual regulation of extinguished fear in humans. *NeuroImage*, *122*, 262–71. http://doi.org/10.1016/j.neuroimage.2015.07.051

Alsina-Jurnet, I., Carvallo-Beciu, C., & Gutiérez-Maldonado, J. (2007). Validity of virtual reality as a method of exposure in the treatment of test anxiety. *Behavior Research Methods*, *39*(4), 844–51. Retrieved from http://www.ncbi.nlm.nih.gov/pubmed/18183899

Alsina-Jurnet, I., & Gutiérrez-Maldonado, J. (2010). Influence of personality and individual abilities on the sense of presence experienced in anxiety triggering virtual environments. *International Journal of Human-Computer Studies*, *68*(10), 788–801. http://doi.org/10.1016/j.ijhcs.2010.07.001

Alsina-Jurnet, I., Gutiérrez-Maldonado, J., & Rangel-Gómez, M.-V. (2011). The role of presence in the level of anxiety experienced in clinical virtual environments. *Computers in Human Behavior*, *27*, 504–512. http://doi.org/10.1016/j.chb.2010.09.018

Anderson, P. L., Price, M., Edwards, S. M., Obasaju, M. A., Schmertz, S. K., Zimand, E., & Calamaras, M. R. (2013). Virtual reality exposure therapy for social anxiety disorder: a randomized controlled trial. *Journal of Consulting and Clinical Psychology*, *81*(5), 751–60. http://doi.org/10.1037/a0033559

Anderson, P. L., Zimand, E., Hodges, L. F., & Rothbaum, B. O. (2005). Cognitive behavioral therapy for public-speaking anxiety using virtual reality for exposure. *Depression and Anxiety*, *22*(3), 156–8. http://doi.org/10.1002/da.20090

Anderson, P., Rothbaum, B. O., & Hodges, L. F. (2003). Virtual reality exposure in the treatment of social anxiety. *Cognitive and Behavioral Practice*, *10*(3), 240–247. http://doi.org/10.1016/S1077-7229(03)80036-6

Aymerich-Franch, L., Kizilcec, R. F., & Bailenson, J. N. (2014). The Relationship between Virtual Self Similarity and Social Anxiety. *Frontiers in Human Neuroscience*, *8*, 944. http://doi.org/10.3389/fnhum.2014.00944

Baños, R. M., Guillen, V., Quero, S., García-Palacios, A., Alcaniz, M., & Botella, C. (2011). A virtual reality system for the treatment of stress-related disorders: A preliminary analysis of efficacy compared to a standard cognitive behavioral program. *International Journal of Human-Computer Studies*, *69*(9), 602–613. http://doi.org/10.1016/j.ijhcs.2011.06.002

Baños, R. M., Botella, C., Perpiñá, C., Alcañiz, M., Lozano, J. A., Osma, J., & Gallardo, M. (2002). Virtual reality treatment of flying phobia. *IEEE Transactions on Information Technology in Biomedicine : A Publication of the IEEE Engineering in Medicine and Biology Society*, *6*(3), 206–12. Retrieved from http://www.ncbi.nlm.nih.gov/pubmed/12381036

Beck, J. G., Palyo, S. A., Winer, E. H., Schwagler, B. E., & Ang, E. J. (2007). Virtual Reality Exposure Therapy for PTSD symptoms after a road accident: an uncontrolled case series. *Behavior Therapy*, *38*(1), 39–48. http://doi.org/10.1016/j.beth.2006.04.001

Botella, C. M., Juan, M. C., Baños, R. M., Alcañiz, M., Guillén, V., & Rey, B. (2005). Mixing realities? An application of augmented reality for the treatment of cockroach phobia. *Cyberpsychology & Behavior : The Impact of the Internet, Multimedia and Virtual Reality on Behavior and Society*, *8*(2), 162–71. http://doi.org/10.1089/cpb.2005.8.162

Botella, C., Baños, R. M., Perpiñá, C., Villa, H., Alcañiz, M., & Rey, A. (1998). Virtual reality treatment of claustrophobia: a case report. *Behaviour Research and Therapy*, *36*(2), 239–46. Retrieved from http://www.ncbi.nlm.nih.gov/pubmed/9613029

Botella, C., Villa, H., Baños, R., Perpiñá, C., & García-Palacios, A. (1999). The treatment of claustrophobia with virtual reality: changes in other phobic behaviors not specifically treated. *Cyberpsychology & Behavior : The Impact of the Internet, Multimedia and Virtual Reality on Behavior and Society*, *2*(2), 135–41. http://doi.org/10.1089/cpb.1999.2.135

Botella, C., Osma, J., Garcia-Palacios, A., Quero, S., & Baños, R. M. (2004). Treatment of flying phobia using virtual reality: data from a 1-year follow-up using a multiple baseline design. *Clinical Psychology & Psychotherapy*, *11*(5), 311–323. http://doi.org/10.1002/cpp.404

Botella, C., Baños, R. M., Villa, H., Perpiñá, C., & García-Palacios, A. (2000). Virtual reality in the treatment of claustrophobic fear: A controlled, multiple-baseline design. *Behavior Therapy*, *31*(3), 583–595. http://doi.org/10.1016/S0005-7894(00)80032-5

Botella, C., Bretón-López, J., Quero, S., Baños, R., & García-Palacios, A. (2010). Treating cockroach phobia with augmented reality. *Behavior Therapy*, *41*(3), 401–13. http://doi.org/10.1016/j.beth.2009.07.002

Botella, C., García-Palacios, A., Guillen, V., Baños, R. M., Quero, S., & Alcaniz, M. (2010). An adaptive display for the treatment of diverse trauma PTSD victims. *Cyberpsychology, Behavior and Social Networking*, *13*(1), 67–71. Retrieved from http://www.ncbi.nlm.nih.gov/pubmed/20528295

Botella, C., Pérez-Ara, M. Á., Bretón-López, J., Quero, S., García-Palacios, A., & Baños, R. M. (2016). In Vivo versus Augmented Reality Exposure in the Treatment of Small Animal Phobia: A Randomized Controlled Trial. *PloS One*, *11*(2), e0148237. http://doi.org/10.1371/journal.pone.0148237

Bouchard, S., Côté, S., St-Jacques, J., Robillard, G. E., & Renaud, P. (2006). Effectiveness of virtual reality exposure in the treatment of arachnophobia using 3D games. *Technology and Health Care*, *14*, 19–27.

Bouchard S, Dumoulin S, Robillard G, Guitard T, Klinger É, Forget H, Loranger C, Roucaut FX. (2016) Virtual reality compared with in vivo exposure in the treatment of social anxiety disorder: a three-arm randomised controlled trial. Br J Psychiatry, Dec 2016, bjp.bp.116.184234

Bouchard, S., St-Jacques, J., Robillard, G., & Renaud, P. (2008). Anxiety Increases the Feeling of Presence in Virtual Reality. *Presence: Teleoperators and Virtual Environments*, *17*(4), 376–391. http://doi.org/10.1162/pres.17.4.376

Bretón-López, J., Quero, S., Botella, C., García-Palacios, A., Baños, R. M., & Alcañiz, M. (2010). An augmented reality system validation for the treatment of cockroach phobia. *Cyberpsychology, Behavior and Social Networking*, *13*(6), 705–10. http://doi.org/10.1089/cyber.2009.0170

Bruce, M., & Regenbrecht, H. (2009). A virtual reality claustrophobia therapy system - implementation and test. In *2009 IEEE Virtual Reality Conference* (pp. 179–182). IEEE. http://doi.org/10.1109/VR.2009.4811020

Burton, M., Schmertz, S. K., Price, M., Masuda, A., & Anderson, P. L. (2013). The relation between mindfulness and fear of negative evaluation over the course of cognitive behavioral therapy for social anxiety disorder. *Journal of Clinical Psychology*, *69*(3), 222–8. http://doi.org/10.1002/jclp.21929

Cárdenas, G., Botella, C., Quero, S., Moreyra, L., De La Rosa, A., & Muñoz, S. (2009). A Cross-Cultural Validation of VR Treatment System for Flying Phobia in the Mexican Population. *Studies in Health Technology and Informatics*, *144*, 141–4. Retrieved from http://www.ncbi.nlm.nih.gov/pubmed/19592751

Carlin, A. S., Hoffman, H. G., & Weghorst, S. (1997). Virtual reality and tactile augmentation in the treatment of spider phobia: a case report. *Behaviour Research and Therapy*, *35*(2), 153–8. Retrieved from http://www.ncbi.nlm.nih.gov/pubmed/9046678

Choi, Y. H., Jang, D. P., Ku, J. H., Shin, M. B., & Kim, S. I. (2001). Short-term treatment of acrophobia with virtual reality therapy (VRT): a case report. *Cyberpsychology & Behavior : The Impact of the Internet, Multimedia and Virtual Reality on Behavior and Society*, *4*(3), 349–54. http://doi.org/10.1089/109493101300210240

Choi, Y.-H., Vincelli, F., Riva, G., Wiederhold, B. K., Lee, J.-H., & Park, K.-H. (2005). Effects of group experiential cognitive therapy for the treatment of panic disorder with agoraphobia. *Cyberpsychology & Behavior : The Impact of the Internet, Multimedia and Virtual Reality on Behavior and Society*, *8*(4), 387–93. http://doi.org/10.1089/cpb.2005.8.387

Clemente, M., Rey, B., Rodriguez-Pujadas, A., Breton-Lopez, J., Barros-Loscertales, A., Baños, R. M., … Avila, C. (2014). A functional magnetic resonance imaging assessment of small animals’ phobia using virtual reality as a stimulus. *JMIR Serious Games*, *2*(1), e6. http://doi.org/10.2196/games.2836

Coelho, C. M., Santos, J. A., Silva, C., Wallis, G., Tichon, J., & Hine, T. J. (2008). The role of self-motion in acrophobia treatment. *Cyberpsychology & Behavior : The Impact of the Internet, Multimedia and Virtual Reality on Behavior and Society*, *11*(6), 723–5. http://doi.org/10.1089/cpb.2008.0023

Coelho, C. M., Santos, J. A., Silvério, J., & Silva, C. F. (2006). Virtual reality and acrophobia: one-year follow-up and case study. *Cyberpsychology & Behavior : The Impact of the Internet, Multimedia and Virtual Reality on Behavior and Society*, *9*(3), 336–41. http://doi.org/10.1089/cpb.2006.9.336

Coelho, C. M., Silva, C. F., Santos, J. A., Tichon, J., & Wallis, G. (2008). Contrasting the Effectiveness and Efficiency of Virtual Reality and Real Environments in the Treatment of Acrophobia. *PsychNology Journal*, *6*(2), 203–216.

Cornwell, B. R., Heller, R., Biggs, A., Pine, D. S., & Grillon, C. (2011). Becoming the center of attention in social anxiety disorder: startle reactivity to a virtual audience during speech anticipation. *The Journal of Clinical Psychiatry*, *72*(7), 942–8. http://doi.org/10.4088/JCP.09m05731blu

Cornwell, B. R., Johnson, L., Berardi, L., & Grillon, C. (2006). Anticipation of public speaking in virtual reality reveals a relationship between trait social anxiety and startle reactivity. *Biological Psychiatry*, *59*(7), 664–6. http://doi.org/10.1016/j.biopsych.2005.09.015

Côté, S., & Bouchard, S. (2005). Documenting the efficacy of virtual reality exposure with psychophysiological and information processing measures. *Applied Psychophysiology and Biofeedback*, *30*(3), 217–32. http://doi.org/10.1007/s10484-005-6379-x

Côté, S., & Bouchard, S. (2009). Cognitive mechanisms underlying virtual reality exposure. *Cyberpsychology & Behavior : The Impact of the Internet, Multimedia and Virtual Reality on Behavior and Society*, *12*(2), 121–9. http://doi.org/10.1089/cpb.2008.0008

Cristea, I. A., Valenza, G., Scilingo, E. P., Szentágotai Tătar, A., Gentili, C., & David, D. (2014). Autonomic effects of cognitive reappraisal and acceptance in social anxiety: evidence for common and distinct pathways for parasympathetic reactivity. *Journal of Anxiety Disorders*, *28*(8), 795–803. http://doi.org/10.1016/j.janxdis.2014.09.009

Czerniak, E., Caspi, A., Litvin, M., Amiaz, R., Bahat, Y., Baransi, H., … Plotnik, M. (2016). A Novel Treatment of Fear of Flying Using a Large Virtual Reality System. *Aerospace Medicine and Human Performance*, *87*(4), 411–6. http://doi.org/10.3357/AMHP.4485.2016

de Quervain, D. J.-F., Bentz, D., Michael, T., Bolt, O. C., Wiederhold, B. K., Margraf, J., & Wilhelm, F. H. (2011). Glucocorticoids enhance extinction-based psychotherapy. *Proceedings of the National Academy of Sciences of the United States of America*, *108*(16), 6621–5. http://doi.org/10.1073/pnas.1018214108

Deppermann, S., Notzon, S., Kroczek, A., Rosenbaum, D., Haeussinger, F. B., Diemer, J., … Zwanzger, P. (2016). Functional co-activation within the prefrontal cortex supports the maintenance of behavioural performance in fear-relevant situations before an iTBS modulated virtual reality challenge in participants with spider phobia. *Behavioural Brain Research*, *307*, 208–17. http://doi.org/10.1016/j.bbr.2016.03.028

Dibbets, P., & Schulte-Ostermann, M. A. (2015). Virtual reality, real emotions: a novel analogue for the assessment of risk factors of post-traumatic stress disorder. *Frontiers in Psychology*, *6*, 681. http://doi.org/10.3389/fpsyg.2015.00681

Diemer, J., Domschke, K., Mühlberger, A., Winter, B., Zavorotnyy, M., Notzon, S., … Zwanzger, P. (2013). Acute anxiolytic effects of quetiapine during virtual reality exposure--a double-blind placebo-controlled trial in patients with specific phobia. *European Neuropsychopharmacology : The Journal of the European College of Neuropsychopharmacology*, *23*(11), 1551–60. http://doi.org/10.1016/j.euroneuro.2013.01.001

Diemer, J., Lohkamp, N., Mühlberger, A., & Zwanzger, P. (2016). Fear and physiological arousal during a virtual height challenge--effects in patients with acrophobia and healthy controls. *Journal of Anxiety Disorders*, *37*, 30–9. http://doi.org/10.1016/j.janxdis.2015.10.007

Difede, J., Cukor, J., Jayasinghe, N., Patt, I., Jedel, S., Spielman, L., … Hoffman, H. G. (2007). Virtual reality exposure therapy for the treatment of posttraumatic stress disorder following September 11, 2001. *The Journal of Clinical Psychiatry*, *68*(11), 1639–47. Retrieved from http://www.ncbi.nlm.nih.gov/pubmed/18052556

Difede, J., Cukor, J., Patt, I., Giosan, C., & Hoffman, H. (2006). The application of virtual reality to the treatment of PTSD following the WTC attack. *Annals of the New York Academy of Sciences*, *1071*, 500–1. http://doi.org/10.1196/annals.1364.052

Difede, J., Cukor, J., Wyka, K., Olden, M., Hoffman, H., Lee, F. S., & Altemus, M. (2014). D-cycloserine augmentation of exposure therapy for post-traumatic stress disorder: a pilot randomized clinical trial. *Neuropsychopharmacology : Official Publication of the American College of Neuropsychopharmacology*, *39*(5), 1052–8. http://doi.org/10.1038/npp.2013.317

Difede, J., & Hoffman, H. G. (2002). Virtual reality exposure therapy for World Trade Center Post-traumatic Stress Disorder: a case report. *Cyberpsychology & Behavior : The Impact of the Internet, Multimedia and Virtual Reality on Behavior and Society*, *5*(6), 529–35. http://doi.org/10.1089/109493102321018169

Donahue, C. B., Kushner, M. G., Thuras, P. D., Murphy, T. G., Van Demark, J. B., & Adson, D. E. (2009). Effect of quetiapine vs. placebo on response to two virtual public speaking exposures in individuals with social phobia. *Journal of Anxiety Disorders*, *23*(3), 362–8. http://doi.org/10.1016/j.janxdis.2008.12.004

Emmelkamp, P. M. G., Krijn, M., Hulsbosch, A. M., de Vries, S., Schuemie, M. J., & van der Mast, C. A. P. G. (2002). Virtual reality treatment versus exposure in vivo: a comparative evaluation in acrophobia. *Behaviour Research and Therapy*, *40*(5), 509–16. Retrieved from http://www.ncbi.nlm.nih.gov/pubmed/12038644

Emmelkamp, P. M., Bruynzeel, M., Drost, L., & van der Mast, C. A. (2001). Virtual reality treatment in acrophobia: a comparison with exposure in vivo. *Cyberpsychology & Behavior : The Impact of the Internet, Multimedia and Virtual Reality on Behavior and Society*, *4*(3), 335–9. http://doi.org/10.1089/109493101300210222

Ferrand, M., Ruffault, A., Tytelman, X., Flahault, C., & Négovanska, V. (2015). A Cognitive and Virtual Reality Treatment Program for the Fear of Flying. *Aerospace Medicine and Human Performance*, *86*(8), 723–7. http://doi.org/10.3357/AMHP.4211.2015

Freedman, S. A., Hoffman, H. G., Garcia-Palacios, A., Tamar Weiss, P. L., Avitzour, S., & Josman, N. (2010). Prolonged exposure and virtual reality-enhanced imaginal exposure for PTSD following a terrorist bulldozer attack: a case study. *Cyberpsychology, Behavior and Social Networking*, *13*(1), 95–101. Retrieved from http://www.ncbi.nlm.nih.gov/pubmed/20528299

Freeman, D., Gittins, M., Pugh, K., Antley, A., Slater, M., & Dunn, G. (2008). What makes one person paranoid and another person anxious? The differential prediction of social anxiety and persecutory ideation in an experimental situation. *Psychological Medicine*, *38*(8), 1121–32. http://doi.org/10.1017/S0033291708003589

Freeman, D., Thompson, C., Vorontsova, N., Dunn, G., Carter, L.-A., Garety, P., … Ehlers, A. (2013). Paranoia and post-traumatic stress disorder in the months after a physical assault: a longitudinal study examining shared and differential predictors. *Psychological Medicine*, *43*(12), 2673–84. http://doi.org/10.1017/S003329171300038X

Freeman, D., Antley, A., Ehlers, A., Dunn, G., Thompson, C., Vorontsova, N., … Slater, M. (2014). The use of immersive virtual reality (VR) to predict the occurrence 6 months later of paranoid thinking and posttraumatic stress symptoms assessed by self-report and interviewer methods: a study of individuals who have been physically assaulted. *Psychological Assessment*, *26*(3), 841–7. http://doi.org/10.1037/a0036240

Gaggioli, A., Pallavicini, F., Morganti, L., Serino, S., Scaratti, C., Briguglio, M., … Riva, G. (2014). Experiential virtual scenarios with real-time monitoring (interreality) for the management of psychological stress: a block randomized controlled trial. *Journal of Medical Internet Research*, *16*(7), e167. http://doi.org/10.2196/jmir.3235

Gamito, P., Oliveira, J., Rosa, P., Morais, D., Duarte, N., Oliveira, S., & Saraiva, T. (2010). PTSD elderly war veterans: a clinical controlled pilot study. *Cyberpsychology, Behavior and Social Networking*, *13*(1), 43–8. Retrieved from http://www.ncbi.nlm.nih.gov/pubmed/20528292

Garcia-Palacios, A., Hoffman, H., Carlin, A., Furness, T. A., & Botella, C. (2002). Virtual reality in the treatment of spider phobia: a controlled study. *Behaviour Research and Therapy*, *40*(9), 983–93. Retrieved from http://www.ncbi.nlm.nih.gov/pubmed/12296495

Gerardi, M., Rothbaum, B. O., Ressler, K., Heekin, M., & Rizzo, A. (2008). Virtual reality exposure therapy using a virtual Iraq: case report. *Journal of Traumatic Stress*, *21*(2), 209–13. http://doi.org/10.1002/jts.20331

Glotzbach-Schoon, E., Tadda, R., Andreatta, M., Tröger, C., Ewald, H., Grillon, C., … Mühlberger, A. (2013). Enhanced discrimination between threatening and safe contexts in high-anxious individuals. *Biological Psychology*, *93*(1), 159–66. http://doi.org/10.1016/j.biopsycho.2013.01.011

Gorini, A., Pallavicini, F., Algeri, D., Repetto, C., Gaggioli, A., & Riva, G. (2010). Virtual reality in the treatment of generalized anxiety disorders. *Studies in Health Technology and Informatics*, *154*, 39–43. Retrieved from http://www.ncbi.nlm.nih.gov/pubmed/20543266

Harris, S. R., Kemmerling, R. L., & North, M. M. (2002). Brief virtual reality therapy for public speaking anxiety. *Cyberpsychology & Behavior : The Impact of the Internet, Multimedia and Virtual Reality on Behavior and Society*, *5*(6), 543–50. http://doi.org/10.1089/109493102321018187

Hartanto, D. W. I., Kang, N., Brinkman, W.-P., Kampmann, I. L., Morina, N., Emmelkamp, P. G. M., & Neerincx, M. A. (2012). Automatic mechanisms for measuring subjective unit of discomfort. *Studies in Health Technology and Informatics*, *181*, 192–6. Retrieved from http://www.ncbi.nlm.nih.gov/pubmed/22954854

Hartanto, D., Kampmann, I. L., Morina, N., Emmelkamp, P. G. M., Neerincx, M. A., & Brinkman, W.-P. (2014). Controlling social stress in virtual reality environments. *PloS One*, *9*(3), e92804. http://doi.org/10.1371/journal.pone.0092804

Herrmann, Katzorke, Busch, Gromer, Polak, Pauli, & Deckert. (2016). Medial prefrontal cortex stimulation accelerates therapy response of exposure therapy in acrophobia. *Brain Stimulation, Brain Stimulation.* *http://dx.doi.org/10.1016/j.brs.2016.11.007*

Hirsch, J. A. (2012). Virtual reality exposure therapy and hypnosis for flying phobia in a treatment-resistant patient: a case report. *The American Journal of Clinical Hypnosis*, *55*(2), 168–73. http://doi.org/10.1080/00029157.2011.639587

Hodges, L. F., Kooper, R., Meyer, T. C., Rothbaum, B. O., Opdyke, D., de Graaff, J. J., … North, M. M. (1995). Virtual environments for treating the fear of heights. *Computer*, *28*(7), 27–34. http://doi.org/10.1109/2.391038

Hoffman, H. G., Garcia-Palacios, A., Carlin, A., Furness III, T. A., & Botella-Arbona, C. (2003). Interfaces That Heal: Coupling Real and Virtual Objects to Treat Spider Phobia. *International Journal of Human-Computer Interaction*, *16*(2), 283–300. http://doi.org/10.1207/S15327590IJHC1602_08

Huang, M. P., Himle, J., & Alessi, N. E. (2000). Vivid Visualization in the Experience of Phobia in Virtual Environments: Preliminary Results. *CyberPsychology & Behavior*, *3*(3), 315–320. http://doi.org/10.1089/10949310050078742

James, L. K., Lin, C.-Y., Steed, A., Swapp, D., & Slater, M. (2003). Social anxiety in virtual environments: results of a pilot study. *Cyberpsychology & Behavior : The Impact of the Internet, Multimedia and Virtual Reality on Behavior and Society*, *6*(3), 237–43. http://doi.org/10.1089/109493103322011515

Jang, D. P., Ku, J. H., Choi, Y. H., Wiederhold, B. K., Nam, S. W., Kim, I. Y., & Kim, S. I. (2002). The development of virtual reality therapy (VRT) system for the treatment of acrophobia and therapeutic case. *IEEE Transactions on Information Technology in Biomedicine : A Publication of the IEEE Engineering in Medicine and Biology Society*, *6*(3), 213–7. Retrieved from http://www.ncbi.nlm.nih.gov/pubmed/12381037

Jang, D. P., Ku, J. H., Shin, M. B., Choi, Y. H., & Kim, S. I. (2000). Objective Validation of the Effectiveness of Virtual Reality Psychotherapy. *CyberPsychology & Behavior*, *3*(3), 369–374. http://doi.org/10.1089/10949310050078814

Johnson, S., Price, M., Mehta, N., & Anderson, P. L. (2014). Stereotype confirmation concerns predict dropout from cognitive behavioral therapy for social anxiety disorder. *BMC Psychiatry*, *14*, 233. http://doi.org/10.1186/s12888-014-0233-8

Josman, N., Reisberg, A., Weiss, P. L., Garcia-Palacios, A., & Hoffman, H. G. (2008). BusWorld: an analog pilot test of a virtual environment designed to treat posttraumatic stress disorder originating from a terrorist suicide bomb attack. *Cyberpsychology & Behavior : The Impact of the Internet, Multimedia and Virtual Reality on Behavior and Society*, *11*(6), 775–7. http://doi.org/10.1089/cpb.2008.0048

Juan, M., Carmen, & David. (2009). Comparison of the levels of presence and anxiety in an acrophobic environment viewed via HMD or CAVE. *Teleoperators and Virtual Environments*, *18*(3), 232–248.

Juan, M. C., & Pérez, D. (2010). Using augmented and virtual reality for the development of acrophobic scenarios. Comparison of the levels of presence and anxiety. *Computers & Graphics*, *34*(6), 756–766. http://doi.org/10.1016/j.cag.2010.08.001

Kahan, M., Tanzer, J., Darvin, D. et al. (2000). Virtual Reality-Assisted Cognitive-Behavioral Treatment for Fear of Flying:...: EBSCOhost. *CyberPsychology*, *3*(3), 387–392. Retrieved from http://ezproxy-prd.bodleian.ox.ac.uk:2091/ehost/pdfviewer/pdfviewer?sid=a286f6df-3221-4613-b920-d95f2b1c40c9%40sessionmgr4010&vid=1&hid=4206

Kampmann, I. L., Emmelkamp, P. M. G., Hartanto, D., Brinkman, W.-P., Zijlstra, B. J. H., & Morina, N. (2016). Exposure to virtual social interactions in the treatment of social anxiety disorder: A randomized controlled trial. *Behaviour Research and Therapy*, *77*, 147–56. http://doi.org/10.1016/j.brat.2015.12.016

Kim, K., Kim, C.-H., Cha, K. R., Park, J., Han, K., Kim, Y. K., … Kim, S. I. (2008). Anxiety provocation and measurement using virtual reality in patients with obsessive-compulsive disorder. *Cyberpsychology & Behavior : The Impact of the Internet, Multimedia and Virtual Reality on Behavior and Society*, *11*(6), 637–41. http://doi.org/10.1089/cpb.2008.0003

Kleim, B., Wilhelm, F. H., Temp, L., Margraf, J., Wiederhold, B. K., & Rasch, B. (2014). Sleep enhances exposure therapy. *Psychological Medicine*, *44*(7), 1511–9. http://doi.org/10.1017/S0033291713001748

Klinger, E., Bouchard, S., Légeron, P., Roy, S., Lauer, F., Chemin, I., & Nugues, P. (2005). Virtual reality therapy versus cognitive behavior therapy for social phobia: a preliminary controlled study. *Cyberpsychology & Behavior : The Impact of the Internet, Multimedia and Virtual Reality on Behavior and Society*, *8*(1), 76–88. http://doi.org/10.1089/cpb.2005.8.76

Krijn, M., Emmelkamp, P. M. G., Olafsson, R. P., Schuemie, M. J., & van der Mast, C. A. P. G. (2007). Do self-statements enhance the effectiveness of virtual reality exposure therapy? A comparative evaluation in acrophobia. *Cyberpsychology & Behavior : The Impact of the Internet, Multimedia and Virtual Reality on Behavior and Society*, *10*(3), 362–70. http://doi.org/10.1089/cpb.2006.9943

Krijn, M., Emmelkamp, P. M. G., Biemond, R., de Wilde de Ligny, C., Schuemie, M. J., & van der Mast, C. A. P. G. (2004). Treatment of acrophobia in virtual reality: the role of immersion and presence. *Behaviour Research and Therapy*, *42*(2), 229–39. http://doi.org/10.1016/S0005-7967(03)00139-6

Krijn, M., Emmelkamp, P. M. G., Olafsson, R. P., Bouwman, M., van Gerwen, L. J., Spinhoven, P., … van der Mast, C. A. P. G. (2007). Fear of flying treatment methods: virtual reality exposure vs. cognitive behavioral therapy. *Aviation, Space, and Environmental Medicine*, *78*(2), 121–8. Retrieved from http://www.ncbi.nlm.nih.gov/pubmed/17310883

La Paglia, F., La Cascia, C., Rizzo, R., Cangialosi, F., Sanna, M., Riva, G., & La Barbera, D. (2014). Cognitive Assessment of OCD Patients: NeuroVR vs Neuropsychological Test. *Studies in Health Technology and Informatics*, *199*, 40–4. Retrieved from http://www.ncbi.nlm.nih.gov/pubmed/24875687

La Paglia, F., La Cascia, C., Rizzo, R., Riva, G., & La Barbera, D. (2012). Assessment of executive functions in patients with obsessive compulsive disorder by NeuroVR. *Studies in Health Technology and Informatics*, *181*, 98–102. Retrieved from http://www.ncbi.nlm.nih.gov/pubmed/22954836

Laforest, M., Bouchard, S., Bossé, J., & Mesly, O. (2016). Effectiveness of In Virtuo Exposure and Response Prevention Treatment Using Cognitive-Behavioral Therapy for Obsessive-Compulsive Disorder: A Study Based on a Single-Case Study Protocol. *Frontiers in Psychiatry*, *7*, 99. http://doi.org/10.3389/fpsyt.2016.00099

Levy, F., Leboucher, P., Rautureau, G., & Jouvent, R. (2016). E-virtual reality exposure therapy in acrophobia: A pilot study. *Journal of Telemedicine and Telecare*, *22*(4), 215–20. http://doi.org/10.1177/1357633X15598243

Levy, F., Leboucher, P., Rautureau, G., Komano, O., Millet, B., & Jouvent, R. (2016). Fear of falling: efficacy of virtual reality associated with serious games in elderly people. *Neuropsychiatric Disease and Treatment*, *12*, 877–81. http://doi.org/10.2147/NDT.S97809

Malbos, E., Mestre, D. R., Note, I. D., & Gellato, C. (2008). Virtual reality and claustrophobia: multiple components therapy involving game editor virtual environments exposure. *Cyberpsychology & Behavior : The Impact of the Internet, Multimedia and Virtual Reality on Behavior and Society*, *11*(6), 695–7. http://doi.org/10.1089/cpb.2007.0246

Malbos, E., Rapee, R. M., & Kavakli, M. (2013). A controlled study of agoraphobia and the independent effect of virtual reality exposure therapy. *The Australian and New Zealand Journal of Psychiatry*, *47*(2), 160–8. http://doi.org/10.1177/0004867412453626

Maltby, N., Kirsch, I., Mayers, M., & Allen, G. J. (2002). Virtual reality exposure therapy for the treatment of fear of flying: a controlled investigation. *Journal of Consulting and Clinical Psychology*, *70*(5), 1112–8. Retrieved from http://www.ncbi.nlm.nih.gov/pubmed/12362961

McLay, R. N., Graap, K., Spira, J., Perlman, K., Johnston, S., Rothbaum, B. O., … Rizzo, A. (2012). Development and testing of virtual reality exposure therapy for post-traumatic stress disorder in active duty service members who served in Iraq and Afghanistan. *Military Medicine*, *177*(6), 635–42. Retrieved from http://www.ncbi.nlm.nih.gov/pubmed/22730837

McLay, R. N., Wood, D. P., Webb-Murphy, J. A., Spira, J. L., Wiederhold, M. D., Pyne, J. M., & Wiederhold, B. K. (2011). A randomized, controlled trial of virtual reality-graded exposure therapy for post-traumatic stress disorder in active duty service members with combat-related post-traumatic stress disorder. *Cyberpsychology, Behavior and Social Networking*, *14*(4), 223–9. http://doi.org/10.1089/cyber.2011.0003

McLay, R., Ram, V., Murphy, J., Spira, J., Wood, D. P., Wiederhold, M. D., … Reeves, D. (2014). Effect of virtual reality PTSD treatment on mood and neurocognitive outcomes. *Cyberpsychology, Behavior and Social Networking*, *17*(7), 439–46. http://doi.org/10.1089/cyber.2013.0383

Meyerbroeker, K., Morina, N., Kerkhof, G. A., & Emmelkamp, P. M. G. (2013). Virtual reality exposure therapy does not provide any additional value in agoraphobic patients: a randomized controlled trial. *Psychotherapy and Psychosomatics*, *82*(3), 170–6. http://doi.org/10.1159/000342715

Meyerbroeker, K., Powers, M. B., van Stegeren, A., & Emmelkamp, P. M. G. (2012). Does yohimbine hydrochloride facilitate fear extinction in virtual reality treatment of fear of flying? A randomized placebo-controlled trial. *Psychotherapy and Psychosomatics*, *81*(1), 29–37. http://doi.org/10.1159/000329454

Michaliszyn, D., Marchand, A., Bouchard, S., Martel, M.-O., & Poirier-Bisson, J. (2010). A randomized, controlled clinical trial of in virtuo and in vivo exposure for spider phobia. *Cyberpsychology, Behavior and Social Networking*, *13*(6), 689–95. http://doi.org/10.1089/cyber.2009.0277

Miyahira, S. D., Folen, R. A., Hoffman, H. G., Garcia-Palacios, A., & Schaper, K. M. (2010). Effectiveness of brief VR treatment for PTSD in war-fighters: a case study. *Studies in Health Technology and Informatics*, *154*, 214–9. Retrieved from http://www.ncbi.nlm.nih.gov/pubmed/20543300

Miyahira, S. D., Folen, R. A., Hoffman, H. G., Garcia-Palacios, A., Spira, J. L., & Kawasaki, M. (2012). The effectiveness of VR exposure therapy for PTSD in returning warfighters. *Studies in Health Technology and Informatics*, *181*, 128–32. Retrieved from http://www.ncbi.nlm.nih.gov/pubmed/22954842

Moore, K., Wiederhold, B. K., Wiederhold, M. D., & Riva, G. (2002). Panic and agoraphobia in a virtual world. *Cyberpsychology & Behavior : The Impact of the Internet, Multimedia and Virtual Reality on Behavior and Society*, *5*(3), 197–202. http://doi.org/10.1089/109493102760147178

Morina, N., Brinkman, W.-P., Hartanto, D., & Emmelkamp, P. M. G. (2014). Sense of presence and anxiety during virtual social interactions between a human and virtual humans. *PeerJ*, *2*, e337. http://doi.org/10.7717/peerj.337

Morina, N., Brinkman, W.-P., Hartanto, D., Kampmann, I. L., & Emmelkamp, P. M. G. (2015). Social interactions in virtual reality exposure therapy: A proof-of-concept pilot study. *Technology and Health Care : Official Journal of the European Society for Engineering and Medicine*, *23*(5), 581–9. http://doi.org/10.3233/THC-151014

Mosig, C., Merz, C. J., Mohr, C., Adolph, D., Wolf, O. T., Schneider, S., … Zlomuzica, A. (2014). Enhanced discriminative fear learning of phobia-irrelevant stimuli in spider-fearful individuals. *Frontiers in Behavioral Neuroscience*, *8*, 328. http://doi.org/10.3389/fnbeh.2014.00328

Mühlberger, A., Herrmann, M. J., Wiedemann, G. C., Ellgring, H., & Pauli, P. (2001). Repeated exposure of flight phobics to flights in virtual reality. *Behaviour Research and Therapy*, *39*(9), 1033–50. Retrieved from http://www.ncbi.nlm.nih.gov/pubmed/11520010

Mühlberger, A., Bülthoff, H. H., Wiedemann, G., & Pauli, P. (2007). Virtual reality for the psychophysiological assessment of phobic fear: responses during virtual tunnel driving. *Psychological Assessment*, *19*(3), 340–6. http://doi.org/10.1037/1040-3590.19.3.340

Mühlberger, A., Neumann, R., Lozo, L., Müller, M., & Hettinger, M. (2012). Bottom-up and top-down influences of beliefs on emotional responses: fear of heights in a virtual environment. *Studies in Health Technology and Informatics*, *181*, 133–7. Retrieved from http://www.ncbi.nlm.nih.gov/pubmed/22954843

Mühlberger, A., Weik, A., Pauli, P., & Wiedemann, G. (2006). One-session virtual reality exposure treatment for fear of flying: 1-Year follow-up and graduation flight accompaniment effects. *Psychotherapy Research*, *16*(1), 26–40. http://doi.org/10.1080/10503300500090944

Mühlberger, A., Wiedemann, G., & Pauli, P. (2010). Efficacy of a One-Session Virtual Reality Exposure Treatment for Fear of Flying. *Psychotherapy Research*.

Ngai, I., Tully, E. C., & Anderson, P. L. (2015). The course of the working alliance during virtual reality and exposure group therapy for social anxiety disorder. *Behavioural and Cognitive Psychotherapy*, *43*(2), 167–81. http://doi.org/10.1017/S135246581300088X

Norrholm, S. D., Jovanovic, T., Gerardi, M., Breazeale, K. G., Price, M., Davis, M., … Rothbaum, B. O. (2016). Baseline psychophysiological and cortisol reactivity as a predictor of PTSD treatment outcome in virtual reality exposure therapy. *Behaviour Research and Therapy*, *82*, 28–37. http://doi.org/10.1016/j.brat.2016.05.002

North, M. M., & Rives, J. (2003). Virtual reality therapy in aid of senior citizens’ psychological disorders. *Studies in Health Technology and Informatics*, *94*, 245–7. Retrieved from http://www.ncbi.nlm.nih.gov/pubmed/15455901

Notzon, S., Deppermann, S., Fallgatter, A., Diemer, J., Kroczek, A., Domschke, K., … Ehlis, A.-C. (2015). Psychophysiological effects of an iTBS modulated virtual reality challenge including participants with spider phobia. *Biological Psychology*, *112*, 66–76. http://doi.org/10.1016/j.biopsycho.2015.10.003

Pallavicini, F., Cipresso, P., Raspelli, S., Grassi, A., Serino, S., Vigna, C., … Riva, G. (2013). Is virtual reality always an effective stressors for exposure treatments? Some insights from a controlled trial. *BMC Psychiatry*, *13*, 52. http://doi.org/10.1186/1471-244X-13-52

Pan, X., Gillies, M., Barker, C., Clark, D. M., & Slater, M. (2012). Socially anxious and confident men interact with a forward virtual woman: an experimental study. *PloS One*, *7*(4), e32931. http://doi.org/10.1371/journal.pone.0032931

Peñate Castro, W., Roca Sánchez, M. J., Pitti González, C. T., Bethencourt, J. M., de la Fuente Portero, J. A., & Marco, R. G. (2014). Cognitive-behavioral treatment and antidepressants combined with virtual reality exposure for patients with chronic agoraphobia. *International Journal of Clinical and Health Psychology*, *14*(1), 9–17. http://doi.org/10.1016/S1697-2600(14)70032-8

Peñate, A., Manuel, J., & la Fuente, D. (2008). The effects of a treatment based on the use of virtual reality exposure and cognitive-behavioral therapy. *International Journal of Clinical and Health Psychology*, *8*(1), 5–22. Retrieved from http://www.redalyc.org/articulo.oa?id=33780101

Peperkorn, H. M., Alpers, G. W., & Mühlberger, A. (2014). Triggers of fear: perceptual cues versus conceptual information in spider phobia. *Journal of Clinical Psychology*, *70*(7), 704–14. http://doi.org/10.1002/jclp.22057

Peperkorn, H. M., Diemer, J. E., Alpers, G. W., & Mühlberger, A. (2016). Representation of Patients’ Hand Modulates Fear Reactions of Patients with Spider Phobia in Virtual Reality. *Frontiers in Psychology*, *7*, 268. http://doi.org/10.3389/fpsyg.2016.00268

Pertaub, D.-P., Slater, M., & Barker, C. (2002). An Experiment on Public Speaking Anxiety in Response to Three Different Types of Virtual Audience. *Presence: Teleoperators and Virtual Environments*, *11*(1), 68–78.

Piercey, C. D., Charlton, K., & Callewaert, C. (2012). Reducing Anxiety Using Self-Help Virtual Reality Cognitive Behavioral Therapy. *Games for Health Journal*, *1*(2), 124–8. http://doi.org/10.1089/g4h.2012.0008

Powers, M. B., Briceno, N. F., Gresham, R., Jouriles, E. N., Emmelkamp, P. M. G., & Smits, J. A. J. (2013). Do conversations with virtual avatars increase feelings of social anxiety? *Journal of Anxiety Disorders*, *27*(4), 398–403. http://doi.org/10.1016/j.janxdis.2013.03.003

Price, M., & Anderson, P. (2007). The role of presence in virtual reality exposure therapy. *Journal of Anxiety Disorders*, *21*(5), 742–51. http://doi.org/10.1016/j.janxdis.2006.11.002

Price, M., & Anderson, P. L. (2012). Outcome expectancy as a predictor of treatment response in cognitive behavioral therapy for public speaking fears within social anxiety disorder. *Psychotherapy (Chicago, Ill.)*, *49*(2), 173–9. http://doi.org/10.1037/a0024734

Price, M., & Anderson, P. L. (2011). The impact of cognitive behavioral therapy on post event processing among those with social anxiety disorder. *Behaviour Research and Therapy*, *49*(2), 132–7. http://doi.org/10.1016/j.brat.2010.11.006

Price, M., Anderson, P., Henrich, C. C., & Rothbaum, B. O. (2008). Greater expectations: using hierarchical linear modeling to examine expectancy for treatment outcome as a predictor of treatment response. *Behavior Therapy*, *39*(4), 398–405. http://doi.org/10.1016/j.beth.2007.12.002

Price, M., Maples, J. L., Jovanovic, T., Norrholm, S. D., Heekin, M., & Rothbaum, B. O. (2015). An investigation of outcome expectancies as a predictor of treatment response for combat veterans with PTSD: comparison of clinician, self-report, and biological measures. *Depression and Anxiety*, *32*(6), 392–9. http://doi.org/10.1002/da.22354

Price, M., Mehta, N., Tone, E. B., & Anderson, P. L. (2011). Does engagement with exposure yield better outcomes? Components of presence as a predictor of treatment response for virtual reality exposure therapy for social phobia. *Journal of Anxiety Disorders*, *25*(6), 763–70. <http://doi.org/10.1016/j.janxdis.2011.03.004>

Ready, D. J., Gerardi, R. J., Backscheider, A. G., Mascaro, N., & Rothbaum, B. O. (2010). Comparing virtual reality exposure therapy to present-centered therapy with 11 U.S. Vietnam veterans with PTSD. *Cyberpsychology, Behavior and Social Networking*, *13*(1), 49–54

Ready, D. J., Pollack, S., Rothbaum, B. O., & Alarcon, R. D. (2006). Virtual Reality Exposure for Veterans with Posttraumatic Stress Disorder. Journal of Aggression, Maltreatment & Trauma, 12(1–2), 199–220. http://doi.org/10.1300/J146v12n01_11

Reger, G. M., & Gahm, G. A. (2008). Virtual reality exposure therapy for active duty soldiers. Journal of Clinical Psychology, 64(8), 940

Reger, G. M., Koenen-Woods, P., Zetocha, K., Smolenski, D. J., Holloway, K. M., Rothbaum, B. O., … Gahm, G. A. (2016). Randomized Controlled Trial of Prolonged Exposure Using Imaginal Exposure vs. Virtual Reality Exposure in Active Duty Soldiers With Deployment-Related Posttraumatic Stress Disorder (PTSD). *Journal of Consulting and Clinical Psychology*, 84, 946-959. http://doi.org/10.1037/ccp0000134

Reger, G. M., Gahm, G. A., Rizzo, A. A., Swanson, R., & Duma, S. (2009). Soldier evaluation of the virtual reality Iraq. Telemedicine Journal and E-Health : The Official Journal of the American Telemedicine Association, 15(1), 101–4. http://doi.org/10.1089/tmj.2008.0050

Reger, G. M., Holloway, K. M., Candy, C., Rothbaum, B. O., Difede, J., Rizzo, A. A., & Gahm, G. A. (2011). Effectiveness of virtual reality exposure therapy for active duty soldiers in a military mental health clinic. Journal of Traumatic Stress, 24(1), 93–6. http://doi.org/10.1002/jts.20574

Renaud, P., Bouchard, S., & Proulx, R. (2002). Behavioral avoidance dynamics in the presence of a virtual spider. IEEE Transactions on Information Technology in Biomedicine : A Publication of the IEEE Engineering in Medicine and Biology Society, 6(3), 235–43.

Ressler, K. J., Rothbaum, B. O., Tannenbaum, L., Anderson, P., Graap, K., Zimand, E., … Davis, M. (2004). Cognitive enhancers as adjuncts to psychotherapy: use of D-cycloserine in phobic individuals to facilitate extinction of fear. Archives of General Psychiatry, 61(11), 1136–44. http://doi.org/10.1001/archpsyc.61.11.1136

Rinck, M., Koene, M., Telli, S., Moerman-Van Den Brink, W., Verhoeven, B., & Becker, E. (2015). The time course of location-avoidance learning in fear of spiders. Cognition and Emotion, 1-14.

Rizzo, A. A., Difede, J., Rothbaum, B. O., Johnston, S., McLay, R. N., Reger, G., … Pair, J. (2009). VR PTSD exposure therapy results with active duty OIF/OEF combatants. Studies in Health Technology and Informatics, 142, 277–82. And Rizzo, A. S., Difede, J., Rothbaum, B. O., Reger, G., Spitalnick, J., Cukor, J., & McLay, R. (2010). Development and early evaluation of the Virtual Iraq/Afghanistan exposure therapy system for combat-related PTSD. Annals of the New York Academy of Sciences

Robillard, G., Bouchard, S., Dumoulin, S., Guitard, T., & Klinger, E. (2010). Using virtual humans to alleviate social anxiety: preliminary report from a comparative outcome study. Studies in Health Technology and Informatics, 154, 57–60.

Robillard, G., Bouchard, S., Fournier, T., & Renaud, P. (2003). Anxiety and presence during VR immersion: a comparative study of the reactions of phobic and non-phobic participants in therapeutic virtual environments derived from computer games. Cyberpsychology & Behavior : The Impact of the Internet, Multimedia and Virtual Reality on Behavior and Society, 6(5), 467–76.

Rothbaum, B. O., Hodges, L. F., Ready, D., Graap, K., & Alarcon, R. D. (2001). Virtual reality exposure therapy for Vietnam veterans with posttraumatic stress disorder. Journal of Clinical Psychiatry, 62(8), 617–22.

Rothbaum, B. O., Hodges, L., Alarcon, R., Ready, D., Shahar, F., Graap, K., … Baltzell, D. (1999). Virtual reality exposure therapy for PTSD Vietnam Veterans: a case study. Journal of Traumatic Stress, 12(2), 263–71. http://doi.org/10.1023/A:1024772308758

Rothbaum, B. O., Hodges, L., Watson, B. A., Kessler, C. D., & Opdyke, D. (1996). Virtual reality exposure therapy in the treatment of fear of flying: a case report. Behaviour Research and Therapy, 34, 477–81.

Rothbaum, B. O., Anderson, P., Zimand, E., Hodges, L., Lang, D., & Wilson, J. (2006). Virtual reality exposure therapy and standard (in vivo) exposure therapy in the treatment of fear of flying. Behavior Therapy, 37(1), 80–90.

Rothbaum, B. O., Hodges, L., Anderson, P. L., Price, L., & Smith, S. (2002). Twelve-month follow-up of virtual reality and standard exposure therapies for the fear of flying. Journal of Consulting and Clinical Psychology, 70, 428–32.

Rothbaum, B. O., Price, M., Jovanovic, T., Norrholm, S. D., Gerardi, M., Dunlop, B., … Ressler, K. J. (2014). A randomized, double-blind evaluation of D-cycloserine or alprazolam combined with virtual reality exposure therapy for posttraumatic stress disorder in Iraq and Afghanistan War veterans. The American Journal of Psychiatry, 171(6), 640–8.

Roy, M. J., Francis, J., Friedlander, J., Banks-Williams, L., Lande, R. G., Taylor, P., … Rothbaum, B. (2010). Improvement in cerebral function with treatment of posttraumatic stress disorder. Annals of the New York Academy of Sciences, 1208, 142–9.

Roy, S., Klinger, E., Légeron, P., Lauer, F., Chemin, I., & Nugues, P. (2003). Definition of a VR-based protocol to treat social phobia. Cyberpsychology & Behavior : The Impact of the Internet, Multimedia and Virtual Reality on Behavior and Society, 6(4), 411–20.

Rus-Calafell, M., Gutiérrez-Maldonado, J., Botella, C., & Baños, R. M. (2013). Virtual reality exposure and imaginal exposure in the treatment of fear of flying: a pilot study. Behavior Modification, 37(4), 568–90.

Safir, M. P., Wallach, H. S., & Bar-Zvi, M. (2012). Virtual reality cognitive-behavior therapy for public speaking anxiety: one-year follow-up. Behavior Modification, 36(2), 235–46. <http://doi.org/10.1177/0145445511429999>

Seinfeld, S., Bergstrom, I., Pomes, A., Arroyo-Palacios, J., Vico, F., Slater, M., & Sanchez-Vives, M. V. (2015). Influence of Music on Anxiety Induced by Fear of Heights in Virtual Reality. Frontiers in Psychology, 6, 1969. http://doi.org/10.3389/fpsyg.2015.01969

Shiban, Y., Brütting, J., Pauli, P., & Mühlberger, A. (2015). Fear reactivation prior to exposure therapy: does it facilitate the effects of VR exposure in a randomized clinical sample? Journal of Behavior Therapy and Experimental Psychiatry, 46, 133–40. http://doi.org/10.1016/j.jbtep.2014.09.009

Shiban, Y., Fruth, M. B., Pauli, P., Kinateder, M., Reichenberger, J., & Mühlberger, A. (2016). Treatment effect on biases in size estimation in spider phobia. Biological Psychology. http://doi.org/10.1016/j.biopsycho.2016.03.005

Shiban, Peperkorn, Alpers, Pauli, & Mühlberger. (2016). Influence of perceptual cues and conceptual information on the activation and reduction of claustrophobic fear. Journal of Behavior Therapy and Experimental Psychiatry, 51, 19-26.

Shiban, Schelhorn, Pauli, & Mühlberger. (2015). Effect of combined multiple contexts and multiple stimuli exposure in spider phobia: A randomized clinical trial in virtual reality. Behaviour Research and Therapy, 71, 45-53.

Shiban, Y., Reichenberger, J., Neumann, I. D., & Mühlberger, A. (2015). Social conditioning and extinction paradigm: a translational study in virtual reality. *Frontiers in Psychology*, *6*, 400. http://doi.org/10.3389/fpsyg.2015.00400

Simeonov, P. I., Hsiao, H., Dotson, B. W., & Ammons, D. E. (2005). Height effects in real and virtual environments. *Human Factors*, *47*(2), 430–8. Retrieved from http://www.ncbi.nlm.nih.gov/pubmed/16170948

Slater, M., Pertaub, D.-P., & Steed, A. (1999). Public Speaking in Virtual Reality: Facing an Audience of Avatars. IEEE Computer Graphics and Applications March/April.

St-Jacques, J., Bouchard, S., & Bélanger, C. (2010). Is virtual reality effective to motivate and raise interest in phobic children toward therapy? A clinical trial study of in vivo with in virtuo versus in vivo only treatment exposure. *The Journal of Clinical Psychiatry*, *71*(7), 924–31. http://doi.org/10.4088/JCP.08m04822blu

Suied, C., Drettakis, G., Warusfel, O., & Viaud-Delmon, I. (2013). Auditory-visual virtual reality as a diagnostic and therapeutic tool for cynophobia. *Cyberpsychology, Behavior and Social Networking*, *16*(2), 145–52. http://doi.org/10.1089/cyber.2012.1568

Taffou, M., Chapoulie, E., David, A., Guerchouche, R., Drettakis, G., & Viaud-Delmon, I. (2012). Auditory-visual integration of emotional signals in a virtual environment for cynophobia. *Studies in Health Technology and Informatics*, *181*, 238–42. Retrieved from http://www.ncbi.nlm.nih.gov/pubmed/22954863

Taffou, M., Guerchouche, R., Drettakis, G., & Viaud-Delmon, I. (2013). Auditory-visual aversive stimuli modulate the conscious experience of fear. *Multisensory Research*, *26*(4), 347–70. Retrieved from http://www.ncbi.nlm.nih.gov/pubmed/24319928

Tart, C. D., Handelsman, P. R., Deboer, L. B., Rosenfield, D., Pollack, M. H., Hofmann, S. G., … Smits, J. A. J. (2013). Augmentation of exposure therapy with post-session administration of D-cycloserine. *Journal of Psychiatric Research*, *47*(2), 168–74. http://doi.org/10.1016/j.jpsychires.2012.09.024

Tortella-Feliu, M., Botella, C., Llabrés, J., Bretón-López, J. M., del Amo, A. R., Baños, R. M., & Gelabert, J. M. (2011). Virtual reality versus computer-aided exposure treatments for fear of flying. *Behavior Modification*, *35*(1), 3–30. http://doi.org/10.1177/0145445510390801

Triscari, M. T., Faraci, P., Catalisano, D., D ’angelo, V., & Urso, V. (2015). Effectiveness of cognitive behavioral therapy integrated with systematic desensitization, cognitive behavioral therapy combined with eye movement desensitization and reprocessing therapy, and cognitive behavioral therapy combined with virtual reality exposure therapy methods in the treatment of flight anxiety: a randomized trial. Neuropsychiatr Dis Treat. 2015; 11: 2591–2598.

Tworus, R., Szymanska, S., & Ilnicki, S. (2010). A soldier suffering from PTSD, treated by controlled stress exposition using virtual reality and behavioral training. *Cyberpsychology, Behavior and Social Networking*, *13*(1), 103–7. Retrieved from http://www.ncbi.nlm.nih.gov/pubmed/20528300

Urech, A., Krieger, T., Chesham, A., Mast, F. W., & Berger, T. (2015). Virtual Reality-Based Attention Bias Modification Training for Social Anxiety: A Feasibility and Proof of Concept Study. *Frontiers in Psychiatry*, *6*, 154. http://doi.org/10.3389/fpsyt.2015.00154

Vermetten, E., Meijer, L., van der Wurff, P., & Mert, A. (2013). The effect of military motion-assisted memory desensitization and reprocessing treatment on the symptoms of combat-related post traumatic stress disorder: first preliminary results. *Studies in Health Technology and Informatics*, *191*, 125–7. Retrieved from http://www.ncbi.nlm.nih.gov/pubmed/23792857

Viaud-Delmon, I., Ivanenko, Y. P., Berthoz, A., & Jouvent, R. (2000). Adaptation as a sensorial profile in trait anxiety: a study with virtual reality. *Journal of Anxiety Disorders*, *14*(6), 583–601. Retrieved from http://www.ncbi.nlm.nih.gov/pubmed/11918093

Viaud-Delmon, I., Warusfel, O., Seguelas, A., Rio, E., & Jouvent, R. (2006). High sensitivity to multisensory conflicts in agoraphobia exhibited by virtual reality. *European Psychiatry : The Journal of the Association of European Psychiatrists*, *21*(7), 501–8. http://doi.org/10.1016/j.eurpsy.2004.10.004

Villa Martin, H., Botella, C., García-Palacios, A., & Osma, J. (2007). Virtual Reality Exposure in the Treatment of Panic Disorder With Agoraphobia: A Case Study. <http://doi.org/10.1016/j.cbpra.2006.01.008>

Vincelli, F., Anolli, L., Bouchard, S., Wiederhold, B. K., Zurloni, V., & Riva, G. (2003). Experiential cognitive therapy in the treatment of panic disorders with agoraphobia: a controlled study. *Cyberpsychology & Behavior : The Impact of the Internet, Multimedia and Virtual Reality on Behavior and Society*, *6*(3), 321–8. <http://doi.org/10.1089/109493103322011632>

Wald, J., & Taylor, S. (2000). Efficacy of virtual reality exposure therapy to treat driving phobia: a case report. *Journal of Behavior Therapy and Experimental Psychiatry*, *31*(3–4), 249–57. Retrieved from http://www.ncbi.nlm.nih.gov/pubmed/11494960

Wald, J., & Taylor, S. (2003). Preliminary research on the efficacy of virtual reality exposure therapy to treat driving phobia. *Cyberpsychology & Behavior : The Impact of the Internet, Multimedia and Virtual Reality on Behavior and Society*, *6*(5), 459–65. http://doi.org/10.1089/109493103769710488

Wallach, H. S., & Bar-Zvi, M. (2007). Virtual-reality-assisted treatment of flight phobia. *The Israel Journal of Psychiatry and Related Sciences*, *44*(1), 29–32. Retrieved from http://www.ncbi.nlm.nih.gov/pubmed/17665808

Wallach, H. S., Safir, M. P., & Bar-Zvi, M. (2009). Virtual reality cognitive behavior therapy for public speaking anxiety: a randomized clinical trial. *Behavior Modification*, *33*(3), 314–38. http://doi.org/10.1177/0145445509331926

Wallach, H. S., Safir, M. P., & Bar-Zvi, M. (2011). Virtual reality exposure versus cognitive restructuring for treatment of public speaking anxiety: a pilot study. *The Israel Journal of Psychiatry and Related Sciences*, *48*(2), 91–7. Retrieved from http://www.ncbi.nlm.nih.gov/pubmed/22120443

Walshe, D. G., Lewis, E. J., Kim, S. I., O’Sullivan, K., & Wiederhold, B. K. (2003). Exploring the use of computer games and virtual reality in exposure therapy for fear of driving following a motor vehicle accident. *Cyberpsychology & Behavior : The Impact of the Internet, Multimedia and Virtual Reality on Behavior and Society*, *6*(3), 329–34. http://doi.org/10.1089/109493103322011641

Webb, A. K., Vincent, A. L., Jin, A. B., & Pollack, M. H. (2015). Physiological reactivity to nonideographic virtual reality stimuli in veterans with and without PTSD. *Brain and Behavior*, *5*(2), e00304. <http://doi.org/10.1002/brb3.304>

Whitney, S. L., Jacob, R. G., Sparto, P. J., Olshansky, E. F., Detweiler-Shostak, G., Brown, E. L., & Furman, J. M. (2005). Acrophobia and pathological height vertigo: indications for vestibular physical therapy? *Physical Therapy*, *85*(5), 443–58. Retrieved from http://www.ncbi.nlm.nih.gov/pubmed/15842192

Wiederhold, B. K., Jang, D. P., Gevirtz, R. G., Kim, S. I., Kim, I. Y., & Wiederhold, M. D. (2002). The treatment of fear of flying: a controlled study of imaginal and virtual reality graded exposure therapy. *IEEE Transactions on Information Technology in Biomedicine : A Publication of the IEEE Engineering in Medicine and Biology Society*, *6*(3), 218–23. Retrieved from http://www.ncbi.nlm.nih.gov/pubmed/12381038

Wiederhold, B. K., Jang, D. P., Kim, S. I., & Wiederhold, M. D. (2002). Physiological monitoring as an objective tool in virtual reality therapy. *Cyberpsychology & Behavior : The Impact of the Internet, Multimedia and Virtual Reality on Behavior and Society*, *5*(1), 77–82. http://doi.org/10.1089/109493102753685908

Wiederhold, B. K., & Wiederhold, M. D. (2003). Three-year follow-up for virtual reality exposure for fear of flying. *Cyberpsychology & Behavior : The Impact of the Internet, Multimedia and Virtual Reality on Behavior and Society*, *6*(4), 441–5. http://doi.org/10.1089/109493103322278844

Wieser, M. J., Pauli, P., Grosseibl, M., Molzow, I., & Mühlberger, A. (2010). Virtual social interactions in social anxiety--the impact of sex, gaze, and interpersonal distance. *Cyberpsychology, Behavior and Social Networking*, *13*(5), 547–54. http://doi.org/10.1089/cyber.2009.0432

Wilhelm, F. H., Pfaltz, M. C., Gross, J. J., Mauss, I. B., Kim, S. I., & Wiederhold, B. K. (2005). Mechanisms of virtual reality exposure therapy: the role of the behavioral activation and behavioral inhibition systems. *Applied Psychophysiology and Biofeedback*, *30*(3), 271–84. http://doi.org/10.1007/s10484-005-6383-1

Won, A. S., Perone, B., Friend, M., & Bailenson, J. N. (2016). Identifying Anxiety Through Tracked Head Movements in a Virtual Classroom. *Cyberpsychology, Behavior and Social Networking*, *19*(6), 380–7. http://doi.org/10.1089/cyber.2015.0326

Wood, D. P., Murphy, J. A., Center, K. B., Russ, C., McLay, R. N., Reeves, D., … Wiederhold, B. K. (2008). Combat related post traumatic stress disorder: a multiple case report using virtual reality graded exposure therapy with physiological monitoring. *Studies in Health Technology and Informatics*, *132*, 556–61. Retrieved from http://www.ncbi.nlm.nih.gov/pubmed/18391367

Wood, D. P., Murphy, J., Center, K., McLay, R., Reeves, D., Pyne, J., … Wiederhold, B. K. (2007). Combat-related post-traumatic stress disorder: a case report using virtual reality exposure therapy with physiological monitoring. *Cyberpsychology & Behavior : The Impact of the Internet, Multimedia and Virtual Reality on Behavior and Society*, *10*(2), 309–15. http://doi.org/10.1089/cpb.2006.9951

Wood, D. P., Murphy, J., McLay, R., Koffman, R., Spira, J., Obrecht, R. E., … Wiederhold, B. K. (2009). Cost effectiveness of virtual reality graded exposure therapy with physiological monitoring for the treatment of combat related post traumatic stress disorder. *Studies in Health Technology and Informatics*, *144*, 223–9. Retrieved from http://www.ncbi.nlm.nih.gov/pubmed/19592768

Wood, D. P., Webb-Murphy, J., Center, K., McLay, R., Koffman, R., Johnston, S., … Wiederhold, B. K. (2009). Combat-related post-traumatic stress disorder: a case report using virtual reality graded exposure therapy with physiological monitoring with a female Seabee. *Military Medicine*, *174*(11), 1215–22. Retrieved from http://www.ncbi.nlm.nih.gov/pubmed/19960832

Wrzesien, M., Alcañiz, M., Botella, C., Burkhardt, J.-M., Bretón-López, J., Ortega, M., & Brotons, D. B. (2013). The therapeutic lamp: treating small-animal phobias. *IEEE Computer Graphics and Applications*, *33*(1), 80–6. http://doi.org/10.1109/MCG.2013.12
