# Supplementary material for: Virtual reality in the assessment, understanding, and treatment of mental health disorders
Source: Psychol Med. 2017 Mar 22;47(14):2393–400. doi: 10.1017/S003329171700040X (PMC5964457; doi:10.1017/S003329171700040X)
Supplement: Supplementary file 1 [file S003329171700040Xsup001.zip › S003329171700040Xsup002.doc]

**Depression literature**

**Supplementary Fig. S2.** PRISMA 2009 Flow Diagram

Records screened
(n = 103 )

Records excluded
(n = 100 )

Full-text articles assessed for eligibility (n = 4 )

Records identified through database searching
(n = 103 )

Additional records identified through other sources
(n = 0 )

**Identification**

**Screening**

Full-text articles excluded, with reasons
(n = 2 )

n = 2, not clear that immersive VR used

**Eligibility**

Studies included in qualitative synthesis
(n = 2 )

**Included**

**Supplementary Table S2.** *Depression studies*

| Authors | Category | N | Participant characteristics | Virtual reality type | Aims | Conclusions |
| --- | --- | --- | --- | --- | --- | --- |
| Falconer et al (2016) | Treatment | 15 | Patients with major depression | High quality HMD and body tracking | A case series concerning body ownership and compassion, testing the effects of patients being compassionate towards a virtual child and then being put in the virtual child’s position and experiencing the compassion played back to them. This occurred in three sessions. | Levels of compassion, criticism, and depression all improved over time but there was no control group. |
| Shah et al (2015) | Treatment | 22 | Inpatients diagnosed with major depression (n=12) or bipolar disorder in depressive episode (n=10). | HMD | A case series test of three daily one hour relaxation sessions in VR (VR DE-STRESS) combined with face-to-face psychoeducation. | The participants were less stressed and depressed but there was no control group. |

**REFERENCES**

Falconer, C.J., Rovira, .A, King JA, Gilbert P, Antley A, Fearon P, Ralph N, Slater M, & Brewin CR.(2016). Embodying self-compassion within virtual reality and its effects on patients with depression. British Journal of Psychiatry Open, 2, 74-80.

Shah LB, Torres S, Kannusamy P, Chng CM, He HG, Klainin-Yobas P. (2015). Efficacy of the virtual reality-based stress management program on stress-related variables in people with mood disorders: the feasibility study. Archives of Psychiatric Nursing, 29, 6-13.
