# Supplementary material for: Virtual reality in the assessment, understanding, and treatment of mental health disorders
Source: Psychol Med. 2017 Mar 22;47(14):2393–400. doi: 10.1017/S003329171700040X (PMC5964457; doi:10.1017/S003329171700040X)
Supplement: Supplementary file 1 [file S003329171700040Xsup001.zip › S003329171700040Xsup003.doc]

**Psychosis studies**

**Supplementary Fig. S3.** PRISMA 2009 Flow Diagram

**Screening**

**Included**

**Eligibility**

**Identification**

Records identified through database searching
(n = 126 )

Additional records identified through other sources
(n = 7 from citations )

Records screened
(n = 133 )

Records excluded
(n = 26 )

Full-text articles assessed for eligibility
(n = 106 )

Full-text articles excluded, with reasons
(n = 62 )

n = 31 no valid VR technique

n=1 unclear whether immersive VR

n = 23 no empirical evidence

n = 4 not peer-reviewed

n = 2 no specific focus on Schizophrenia/Psychosis in terms of assessment, mechanisms or treatment

n = 1 duplicate

Studies included in qualitative synthesis
(n = 44 )

**Supplementary Table S3.** *Psychosis studies*

| **Authors** | **Category** | **N** | **Participant characteristics** | **Virtual Reality type** | **Aim/Hypothesis** | **Conclusions** |
| --- | --- | --- | --- | --- | --- | --- |
| **1. Atherton, S. et al. (2016)** | Theory | 26 | Males reporting paranoid thinking in past month | HMD. 6-minute underground train journey. | Investigate idea that paranoia may be built upon negative view of the self. Manipulated self-esteem in individuals vulnerable to paranoia and considered the immediate effects on paranoia. | Low self-esteem contributory cause of paranoia. |
| **2. Brinkman, W. et al. (2011)** | Theory | a) 2 b) 24 | a) Male patients with either delusional disorder or schizoaffective disorder b) Non-patients of University staff and students (20 = male, 4 = female) | 3D visor (40° diagonal field of view) with 3DOF tracker. AVATARS in virtual bar with indoor and outdoor setting. | Manipulation of potential social environment stressors (ethnicity proportion and density of avatars) in VR bar in order to investigate psychotic experiences. Used measures of discomfort and physiological arousal to determine paranoia and social anxiety levels. | Patients covered smaller area of the bar, had higher levels of physiological arousal and subjective distress - suggests greater paranoia and social anxiety as well as greater cognitive/emotional strain in the VR situation. Most affected by gender (female) and ethnicity (other), however positioned themselves more closely to the avatars. |
| **3. Broome, M. R. et al. (2013)** | Assessment | 32 | Non-clinical volunteers of undergraduate and postgraduate students (23 = male, 9 = female) | HMD and motion tracking system.  Deprived urban setting. | Builds upon research that has focused on the role of urban upbringing and social deprivation in the aetiology of psychosis. Investigates this by testing whether an urban VR scene (bus stop) provokes same paranoid responses as indoor scenes. | 65.5% of participants experienced some persecutory ideation, suggesting VR is an effective tool and supporting the notion that urban factors play a role in the genesis and maintenance of psychotic experiences, due to VR exposure being able to induce such ideation. |
| **4. da Costa, R. et al. (2004)** | Assessment | 4  *- 1 person did not participate in HMD I-glasses condition.* | Diagnosis of schizophrenia according to DSM-IV. (3 = male, 1 = female) | Computer and HMD I-glasses. Scene of example American city. | Investigated the acceptance of VR in patients with schizophrenia, considering their interest and any potential problems when carrying out various tasks related to each level of performance from the cognitive model. | Participants found VR environment enjoyable and had the ability to navigate using the programme with no side effects. Suggests VR is an appropriate and engaging platform for future research and interventions. |
| **5. Fornells-Ambrojo, M. et al (2008)** | Assessment | a) 20b) 20 | a) Patients aged 16-35 with diagnosis of non-affective psychosis and a current persecutory delusion. (17 = male, 3 = female)b) Non-clinical controls (age matched). (19 = male, 1 = female) | CAVE. Neutral environment -Underground train journey. | Investigated whether it is acceptable and safe to use VR environments and technology to research persecutory delusions in those currently experiencing such delusions. | High sense of presence with no unpleasant/intrusive thoughts or emotions reported by both groups, suggesting VR technology can be safely used to create a realistic-feeling environment with individuals experiencing persecutory delusions. Persecutory ideation in VR was positively associated with higher persecutory ideation in the real world. |
| **6. Fornells-Ambrojo, M. et al (2015)** | Assessment | a) 20 b) 20 | a) Patients aged 16-35 with diagnosis of non-affective psychosis and a current persecutory delusion. (All male) b) Non-clinical controls (age matched). (8 = male, 2 = female) | CAVE. Neutral environment -Underground train ride with avatars. | (Secondary analysis of 2008 paper) Considers how environmental factors relate to psychosis, and whether the on-going interaction helps to maintain psychotic symptoms. | Analysis suggested clinical participants are able to use a range of strategies when evaluating a potential interpersonal threat.  When assessing threat, clinical participants are less likely to engage in active-hypothesis testing than non-clinical controls, instead being likely to use own affective state as evidence of persecution. |
| **7. Fornells-Ambrojo et al (2016)** | Theory | 61 | Non-clinical volunteers | CAVE | Participants interacted with a pleasant avatar programmed to be responsive or non-responsive. | This was a non-clinical pilot study. Higher paranoia was associated with greater distance from the avatar and with greater trust in the responsive avatar. |
| **8. Freeman, D. et al. (2014)** | Assessment | 106 | Individuals who had experienced a distressing assault within the previous month. (79 = male, 27 = female) | HMD. 4-minute underground train journey. | Investigated the validity of the use of VR and whether it can be used to provide accurate symptoms assessment with predictive ability. Assessed occurrence of paranoid (and PTSD) symptoms in individuals after experiencing a single neutral VR social environment to test this. | Symptoms of paranoia and PTSD in the VR environment predicted severity of these symptoms 6 months later (obtained through self-report and interviewer assessment). Suggests VR is a helpful assessment tool. |
| **9. Freeman, D. et al. (2015)** | Theory | 121Placebo = 41, THC = 41, THC+ Awareness = 39 | Aged 21-50, had taken cannabis 1 or more times before, reported paranoid thoughts in last month. | HMD. 5-minute underground train journey. | Aimed to determine whether cannabis causes paranoia, using THC (principal psychoactive ingredient of cannabis) to identify the cognitive mechanisms that underlie paranoia. Conducted a placebo-controlled trial using THC and assessed paranoia symptoms. | Found that THC significantly increased paranoia, negative affect, anomalous experience and reduced working memory. Increase in paranoia was accounted for by increases in negative affect and anomalous experiences. Use of VR enabled demonstration that THC triggers paranoid thoughts in vulnerable individuals. |
| **10. Freeman, D. et al. (2014)** | Theory | 60 | Females experiencing paranoid thinking in past month | HMD. Underground train journey. | Focused on the idea that mistrust of others is related to perception of self as vulnerable, and that paranoia is associated with perceived lower social rank. Investigated this by using height as a proxy for social rank on paranoia (as it is an indicator of social status and authority), with participants taking one VR tube ride at own height and another taken at reduced height. | Reduced height significant lowered social comparison scores (more negative) and increased paranoia.  Mediation tests showed that social comparison fully mediated the relationship between height and paranoia.   Provides evidence that social status plays an important role in paranoid fears and that paranoia is built upon perceived self-vulnerability. |
| **11. Freeman, D. et al. (2005)** | Theory | 30 | Survey respondents with range of paranoia scores. (Equal number of males and females) | CAVE. Neutral library scene with 5 avatars. | The authors’ cognitive model of persecutory delusions was used to predict occurrence of paranoid thoughts in non-clinical participants in a VR environment. | Persecutory ideation seen amongst non-clinical subjects in a neutral environment and was predicted by higher levels of paranoia, timidity, hallucinatory experiences and sense of presence. Experience of persecutory ideation in VR was associated with having similar thoughts in real life, suggesting VR is a useful tool for examining real world experiences. |
| **12. Freeman, D. et al. (2008)** | Theory | 200 | Non-clinical adult volunteers (Equal number of males and females) | HMD. 4-minute underground train journey. | Paranoia is unfounded mistrust, which when severe is a key symptom of schizophrenia. Aim of study was to show that those without mental illness also experience unfounded paranoia, and to determine predictive factors of this. | 40% of sample experienced paranoid thoughts, demonstrating that non-clinical subjects can also experience paranoid thinking and that it is not confined to those with severe mental health problems. Higher levels of catastrophising worry, worry style, perceptual anomalies and cognitive inflexibility all predicted paranoid thinking, demonstrating that paranoia involves emotional processes. |
| **13. Freeman, D. et al. (2007)** | Assessment | 185 | Individuals from 3 separate studies: 100 = volunteers, 64 = university students, 21 = patients at high risk of developing psychosis. | 1 study used HMD, other 2 used CAVE. | Considered the reliability and validity of the first state measure of paranoia: The State Social Paranoia Scale, by examining its psychometric properties. All studies included in assessment followed outline of Freeman et al. '03. | Strong internal reliability of SPSS found in all 3 study populations. SSPS significantly correlated with scores from ratings obtained in the structured interviews of VR experience in all 3 study populations, suggesting it is a valid measure.  Convergent and divergent validity was also revealed in analysis, overall suggesting SPSS is a suitable measure of persecutory ideation for research use. |
| **14. Freeman D. et al. (2010)** | Assessment | a) 30b) 30c) 30 | a) Volunteers with low non-clinical paranoiab) Volunteers with high non-clinical paranoiac) Patients with persecutory delusions (clinical diagnosis of either schizophrenia, schizo-affective disorder or delusion disorder) | HMD. 4-minute underground train journey. | Used VR to validate participants with varying levels of unfounded persecutory ideation and assess whether clinical and nonclinical paranoia is associated with similar causal factors. | Delusions group was 12x more likely to report paranoia ideation in VR than the low non-clinical paranoia group. High non-clinical paranoia group were around 3x more likely to report paranoid ideation than low non-clinical group. Step-change differences in levels of anxiety, worry, interpersonal sensitivity, depression, anomalies of experience and trauma history between groups, suggesting that non-clinical persecutory ideation and clinical paranoia are related experiences - supporting notion of paranoia as a spectrum. |
| **15. Freeman, D. et al. (2003)** | Assessment | 24 | University students and staff with no history of mental illness. (Equal number of males and females.) | CAVE. Neutral library scene with 5 avatars. | Investigated use of VR technology for understanding persecutory ideation. Examined whether participants experience persecutory thoughts about VR characters and if there are factors that predict the occurrence of such thoughts. | Participants attributed mental states to visual characters, suggesting that individuals in virtual environments believe computerized characters have intentions towards them.  High interpersonal sensitivity (most significantly) and anxiety levels predicted higher levels of persecutory ideation in the VR environments, suggesting these two factors are central to feelings of vulnerability. |
| **16. Ku, J. et al. (2006)** | Assessment | 11 | Patients with schizophrenia diagnosis (5 = male, 6 = female) | PC with projection screen, joystick | Investigated whether a virtual avatar could be perceived as a real human by patients with schizophrenia, and whether an avatar could be applied to acquiring patients' behaviour characteristics in a short conversation situation. Participants had to approach avatar and answer questions/converse in order to assess interpersonal characteristics.7 participants experienced task twice. | Interpersonal distance was negatively correlated with negative symptomology. Behaviour of participants suggests perceived avatar as real, suggesting VR could prove a beneficial technique for training and assessment. 5 of 7 participants who experienced task twice decreased their interpersonal distance to the avatar and their verbal response times on second trial - a training effect that could reflect participants becoming more comfortable. |
| **17. Park, K. et al. (2009)** | Assessment | a) 18 b) 15 | a) Female inpatients with paranoid schizophrenia (DSM-IV diagnosis, aged 18-45) b) Female non-clinical controls | HMD. 6 scenarios of common conversation situations. | Aim was to investigate the use of Virtual Reality Functional Skills Assessment (VRFSA) for future use in a clinical trial. In addition investigated effectiveness of atypical antipsychotics to social competence in schizophrenia. | Significant differences between patient and control groups across all parameters, supporting the sensitivity of the VRFAS scale at detecting functional ability. Strong correlations to performance-based measurements (not the RCS self-report measure), overall supporting the use of VRFAS scale in future research. |
| **18. Rus-Calafell, M. et al. (2013)** | Assessment | 12 | Clinically stabilized outpatients with schizophrenia or schizoaffective disorder. | Laptop with 3D glasses and headphones. Soskitrain programme. | Developed an integrated VR programme that focuses on the improvement of social skills of those with schizophrenia. Evaluated the relationship between patients' cognitive deficits, their sense of presence and their ratings of the SOSKITRAIN programme's acceptability. | High sense of presence, verisimilitude of VR environments and acceptability ratings of VR system amongst participants.  Delayed verbal learning and processing speed both negatively correlated with sense of presence. Suggests that whilst the programme may be useful in further research, cognitive deficits modulate the sense of presence achieved. |
| **19. Shaikh, M. et al. (2016)** | Theory | a) 64b) 43 | a) Ultra high risk for psychosis young adult patients. (38 = male, 26 = female)b) 43 non-clinical controls (matched for demographic factors) (20 = male, 23 = female) | HMD. Underground train journey. | Investigated the relationship between perceived ethnic discrimination and paranoid ideation in individuals at Ultra High Risk (UHR) for psychosis using a VR paradigm. Aimed to further understand nature of psychosocial adversity in relation to psychosis onset. | Higher levels of perceived ethnic discrimination in UHR group than controls - perceived ethnic discrimination correlated with persecutory ideation across participants (no relationship, predictor or otherwise specifically in UHR group). Perceived ethnic discrimination was a predictor of persecutory ideation in non-clinical controls, suggesting that it does play a role in development of paranoia, outside of prodromal symptomology. |
| **20. Sorkin, A. et al. (2006)** | Assessment | a) 39 b) 21 | a) Male patients with schizophrenia  b) Non-clinical controls (gender, age and education level matched) | Computer with 3D glasses, head tracker, joystick. | Developed a multimodal VR environment that allows for the assessment and measuring of cognitive functions with the aim of improving diagnosis of schizophrenia. | Difference in performance profile enabled the separation of schizophrenia patients from controls - 85% successfully predicted.   (Patients displayed increased error rate when using the rule, increased consecutive errors and large head rotation compared to controls. Patient group generally demonstrated decreased ability to ignore irrelevant information. However, patient group showed bigger improvements in RT and navigation speed.) |
| **21. Stinson, K. et al. (2010)** | Theory | 30 Exp. Group = 15, control = 15 | Individuals experiencing auditory hallucinations - occurring 1< times a day, occurring in social contexts. (20 = male, 10 = female) | HMD. 4-minute underground train journey. | Investigated the role of cognition in triggering auditory hallucinations.  Participants experience a neutral, non-threatening VR environment with avatars - experimental group instructed to focus on thoughts that precede their hallucinations, controls to focus on neutral thoughts. | 93% of all participants were able to spontaneously identify cognitive antecedents of hallucination. No evidence that these cognitions were triggers of hallucinations.  Occurrence and quality of hallucinations did not differ between groups. |
| **22. Valmaggia, L. et al. (2007)** | Assessment | 21 | Patients at high risk of psychosis.(13 = male, 8 = female) | HMD. Underground train journey. | Sought to establish the feasibility and safety of using VR techniques and methodology for clinical purposes, as well as investigating how far the cognitive model of paranoia can be applied to people with an at-risk mental state. | Participants more likely to experience paranoid thoughts in real life also experienced unfounded paranoid thoughts about VR characters, provides validation of the methodology. Persecutory ideation in the VR environment was associated with higher levels of anxiety, stress, interpersonal sensitivity, perseverative thinking style and immersion in VR, however no VR-induced distress (anxiety of intrusive thoughts) was reported. |
| **23. Veling, W. et al. (2014)** | Theory | a) 17 b) 24 | a) Patients with first episode psychosis (age 18-40 years, DSM-IV diagnosis of schizophrenia or related. b) Non-clinical controls (gender matched) | 3D visor with gamepad. Café with indoor and outdoor sections. | Investigated the feasibility and any possible negative side effects of exposure to different virtual social risk environments in those who have experienced psychosis. Population density and ethnicity of avatars were manipulated to create these social risk environments. | Both groups frequently reported paranoid ideation and paranoia in real world correlated with experiences of paranoia in VR - suggests feasible to use VR in research of psychosis.  Patient group kept smaller distance to avatars than the healthy controls, and also showed higher GSR in VR environments with higher proportion of avatars of different ethnicity to own. |
| **24. Veling, W. et al. (2016)** | Theory | a) 55b) 20c) 42d) 53 | a) Patients with psychotic disorder (42 = male, 13 = female)b)Patients at ultra high risk for psychosis (7 = male, 13 = female) c) Siblings of patients with a psychotic disorder (no psychotic episodes themselves) (23 = male, 19 = female)d) Controls with a negative (first-degree family) history of any psychotic disorder. (25 = male, 28 = female) | HMD, gamepad for movement. Bar environment. | Investigated sensitivity to environmental social stress as a mechanism of psychosis. Participants walked 5 times in a VR bar with different levels of environmental social stress (Virtual social stressors were population density, ethnic density and hostility). | Paranoia and subjective stress increased with the degree of environmental social stress. More paranoia and distress in social environments was associated with high psychosis liability, pre-existing minor affective symptoms and (to lesser degree) psychotic symptoms. Pre-existing symptoms were shown to have a stronger impact on paranoia and distress when environmental social stress level increased. |
| **25. Han, K. et al. (2014)** | Theory | a) 23b) 22 | a) Patients with schizophrenia (DSM-IV diagnosis) from outpatient clinicb) Non-clinical controls (age and gender matched) | HMD. Different conversation scenarios. | Investigated features of social behaviours of patients with schizophrenia using a multi-party conversation task.Participants engaged in four situations in VR environments involving leading and aiding avatars, with varying positive and negative emotions in order to assess participant's social behaviours. | Participants watched leading avatar significantly longer than the aiding avatar, however looked at leading avatar a bit less and aiding avatar a bit more in negative situations, possibly due to the experience of stress (from the negative situation) causing them to employ a coping strategy. Less reasoning amongst the patient group was associated with less focus on lead avatar, suggesting that accurate reasoning is a key component in social interaction. Both groups took longer to start speaking in negative situations, contrary to prior results. Patient group took longer to start speaking in positive and negative situations. |
| **26. Han, K. et al. (2012)** | Theory | a) 30  b) 30 | a) Patients with schizophrenia (DSM-IV diagnosis)  b) Non-clinical controls (age and gender matched) | Beam projector, with speakers and joystick. Bus journey task. | Investigate cognitive flexibility using a VR task. | Overall patient group was much less cognitively flexible than controls.  Non-flexible patients experienced greater stress during decision making, but more satisfaction in their decisions.  Patient negative symptomology negatively correlated with decision-making time - possible due to the making of quick judgements to avoid prolonged feelings of stress. |
| **27. Kim, K. et al. (2007)** | Theory | a) 30  b) 30 | a) Inpatients diagnosed with schizophrenia b) Controls (broadly matched for gender, age and attitude to VR situation) | PC with projection screen, joystick | Considered the use of VR in social training - determine whether VR can be used to measure social skills and social perception and which VR parameters are related to symptoms of schizophrenia. VR environments involved non-verbal and verbal social cues, as well as happy, sad, or angry situations in order to assess. | Schizophrenia group showed poorer social perception ability than non-clinical group.  Patient group found recognition of negative emotions more challenging than that for positive emotions.  Severity of patient symptomology correlated with increased difficulty in emotion recognition. |
| **28. Ku, J. et al. (2003)** | Theory | a) 13b) 13 | a) Patients (8 = male, 5 = female)b) Controls (6 = male, 7 = female) | HMD, joystick. Environment of rooms resembling Egyptian pyramids. | Propose a VR system for the multi-modal assessment of the cognitive ability of people with schizophrenia. The system provides multimodal stimuli (eg. visual and auditory stimuli) to the patient which they must interpret and react to, which allows for the evaluation of the patient's multimodal integration and WM integration abilities | Opening door and navigation performance was better in control group than patient group. Perseverance seen in patient group, particularly when distractors introduced. Task performance parameters significantly correlated with WCST and SPM, suggesting it is a valid and useful neuropsychological test measure. |
| **29. Park, I. et al. (2009)** | Theory | a) 27 b) 27 | a) Patients hospitalized for schizophrenia b) Non-clinical controls (age and gender matched) | HMD. Room with avatar in distance. | Used a social encounter task to investigate dysfunctional emotional processing in individuals with schizophrenia. Sought to explain relationship between emotional perception and response in social interaction. | Both groups demonstrated expected decreases in valence scores from happy, neutral to angry conditions - patient group scored significantly lower than controls for valence in angry condition. Happy and angry conditions produced significantly higher arousal scores than neutral condition in both groups - patient group scored significantly lower than controls for arousal in angry condition.   No relief of anxiety was observed in patient group from encounters with neutral/happy avatars (unexpected, novel finding) and trait anxiety was most likely to affect state anxiety responses in the various conditions.   Suggests that schizophrenia patients experience deficient emotional perception of anger - impairment of negative facial affect. |
| **30. Park, S. et al. (2009)** | Theory | a) 30 b) 30 | a) Patients with schizophrenia (DSM-IV diagnosis) b) Non-clinical controls (broadly age and gender matched) | HMD. 6 interactions with an avatar of varying emotion. | Investigated the social aspects of schizophrenia by focusing on personal space, evaluating the relationship between patient's social behaviours and schizophrenia symptoms. | Patient group stood further away from avatars and had larger angles of head orientation than controls, suggesting increased deviated eye gaze -possibly reflecting the discomfort of close interpersonal situation, or impaired cognitive skill (sensory gating). Negative symptomology was inversely correlated with distance from angry avatars, but not with happy avatars. Male patients interacted more closely with avatars than female patients, and also showed no significant difference in distance according to avatar’s emotions - suggests gender differences in social functioning. |
| **31. Park, S. et al. (2014)** | Theory | a) 27 b) 30 | a) Outpatients with schizophrenia (DSM-IV) diagnosis b) Non-clinical controls (age and gender matched) | Unclear. | Used VR tasks that simulate complicated social situations to investigate the level of intimacy formation and effect that intimacy has on social decision in those with schizophrenia. | Patients intimacy scores were significantly higher than controls for distant avatars, and had significantly smaller increases in acceptance rate for intimate vs. distant avatars. Patients also refused more of the challenging requests than did control (particularly if stronger self-esteem), overall suggesting those with schizophrenia have an emotional perception and social decision-making deficit. |
| **32. Salgado-Pineda, P. et al. (2016)** | Theory | a) 27 b) 32 | a) Right-handed patients with schizophrenia (DSM-IV diagnosis)  b) Right-handed non-clinical controls (age, gender and IQ-matched) | VR glasses (fMRI-safe), with joystick. | Used a VR spatial navigation task to examine hippocampal function, with the aim of assessing task-related activations and deactivations associated with schizophrenia (using fMRI). | Patients required more time to memorize VR city environment and showed lower navigational efficiency.  Activations in widespread brain areas, including hippocampus, parahippocampal gyrus (bilaterally) and several neocortical regions (retrosplenial cortex, PFC), however patient group showed reduced activity in left DLPFC, left occipital and left temporal cortex. Supports notion of task-related hypofrontality in schizophrenia patients. |
| **33. Siemerkus, J. et al. (2012)** | Theory | a) 16b) 16 | a) Inpatients with schizophrenia (DSM-IV diagnosis)b) Non-clinical controls (broadly age, gender and years in education matched on group basis) | HMD, joystick. Maze environment. | Investigated whether disturbed self-recognition and experience of agency contribute to wider egocentric deficits. Conducted fMRI whilst participants undertook a maze navigation task. | Patient showed impaired performance compared to controls, with positive symptoms in schizophrenia patients positively predicting virtual maze performance - stronger symptoms predicted worse performance. Broadly similar pattern of brain activations during virtual learning maze task in both groups, however, controls showed significantly stronger activations in right hemisphere task-relevant regions.Patient group displayed weaker activity changes during virtual maze learning, as well as a differing course of activation across trials.Activity in the right-sided precuneus positively correlated with psychotic symptoms and virtual maze errors. |
| **34. Sorkin, A. et al. (2008)** | Theory | a) 43 b) 29 | a) Patients with schizophrenia (23 inpatients, 20 outpatients) b) Non-clinical controls (age, education and gender-matched) | HMD. Residential neighbourhood, shopping centres and street market environment. | Used a VR environment to measure the distortion in reality perception in patients with schizophrenia. Participants had to navigated a VR environment were they were asked to detect incoherencies (eg. a cat barking or a tree with red leaves) | 88% of patients differed significantly from control group performance, showing difficulties in detecting incoherent stimuli (greatest difficulty in audio-visual incoherencies). Poor performance correlated with presence of hallucinations. |
| **35. Valmaggia, L. et al. (2015)** | Theory | a) 64 b) 43 | a) Patients ultra high risk for psychosis (38 = male, 26 = female) b) Non-clinical controls (20 = male, 23 = female) | HMD. 5-minute London Underground train journey. | Aimed to establish whether previous experience of bullying is associated with higher levels of paranoid ideation using a VR environment. | History of bullying was associated with higher levels of paranoid ideation (independent of clinical status). UHR group reported significantly higher levels of bullying victimization experiences and were more likely to experience paranoid ideation. Possible influence of bullying experience on cognitions, influencing the development of psychotic symptoms. |
| **36. Valmaggia, L. et al. (2015)** | Theory | a) 64b) 43 | a) Patients ultra high risk for psychosis (38 = male, 26 = female)b) Non-clinical controls (20 = male, 23 = female) | HMD. 4-minute underground train journey. | Used a VR social environment to investigate the relationship between social defeat and paranoid appraisal in individuals at high risk for psychosis. | UHR group reported higher levels of social defeat than controls and were more likely to make paranoid appraisals of VR experience. (In UHR group) social defeat level was a significant predictor paranoid ideation in VR. Positive and disorganized symptom severity positively correlated with paranoid appraisals, raising possibility that attenuated positive symptoms mediates the relationship between social defeat and paranoid ideation. |
| **37. Chan, C. et al. (2010)** | Treatment | 29 Intervention group = 14, Control = 15 | Older adults with chronic schizophrenia (60 years old and older, schizophrenia more that 20 years diagnosed using DSM-III) with moderate level of cognitive impairment (MMSE score of 15-23) | IREX - 2D programme, participants stand/sit in demarcated area, large screen display. Animations responsive to participant's movement. | Investigate the effects of a 10-week cognitive training programme for older adults with chronic schizophrenia - 'ball and bird' and 'shark bait' fluid-intelligence based activities. | Training significantly improved the overall cognitive functioning (with no significant cybersickness symptoms) compared with no treatment; possibly due to practice engaging and using attentional processes in the VR task (although whilst repetition and memory improved, attention did not - believed to be due to the ceiling effect of the Cognistat subscale used). Treatment improved participant's volition to engage with the programme, suggesting familiarity with an activity can increase motivation to engage. |
| **38. Freeman, D. et al. (2016)** | Treatment | 30VR CBT = 15, VR exposure = 15. | Patients with persecutory delusions - diagnosis of non-affective psychosis with a current persecutory delusions | HMD. 60-90min sessions. Either underground train or lift. | Focused on the idea that persecutory delusions may be unfounded threat beliefs, maintained through safety-seeking behaviours that prevent disconfirmatory evidence being obtained and processed. Used either VR exposure alone or VR CBT (where threat beliefs of the participant were challenged) to test this idea, assessing conviction in delusions and distress. | Delusion conviction, paranoia conviction and distress, and real-world situation distress ratings reduced in VR CBT group compared to VR exposure group. |
| **39. Ku, J. et al. (2007)** | Treatment | 10 | Patients with schizophrenia diagnosis (5 = male, 5 = female) | Projection screen, with joystick. | Used VR environments to train and develop conversational skills in people with schizophrenia s in specific situations. Hypothesised that this technique could overcome the shortcomings of or complement conventional role-playing techniques. | Presence was negatively correlated with emotional withdrawal symptom, suggesting effects of VR interventions may vary with disease severity.  Negative correlation between social presence score and silence-breaking times - silence-breaking was also related to patients' symptoms concerning conversation flow.  Suggest symptom variation in regard to emotion is a key factor in conversation ability. |
| **40. Park, K. et al. (2011)** | Treatment | 91 | Inpatients with schizophrenia diagnosis (DSM-IV). | HMD. SST-VR included core features of role playing games, therefore participants provided with joystick and buttons. | Investigated the use of social skills training (SST) on a VR platform. Compared SST using VR role-playing (SST-VR) to SST using traditional role-playing (SST-TR). 10 semi-weekly group sessions. | SST-VR showed greater interest and motivation in the training than SST-TR. Significant improvements in social skills seen in both groups - SST-VR group particularly showed improvement in conversational skills, and SSTTR showed particular improvement in vocal and non-verbal skills. Suggests SST-VR enhanced reciprocal interaction skills, and SST-TR enhanced unidirectional interaction skills. |
| **41. Rus-Calafell, M. et al. (2012)** | Treatment | 1 | 30 year old female with established schizophrenia diagnosis. | Laptop with 3D glasses and headphones. Soskitrain programme. | Case study of a 30 year old woman with an established case of schizophrenia using an integrated VR programme in social skills training (SST) and evaluated its effectiveness. | Improvements in social behaviour, negative symptoms and overall psychopathology. Participant found programme design of practising components first in a VR environment before implementing in real world to be useful, as enabled her to build up confidence. Generalizability of improvements made to patient's daily life. |
| **42. Rus-Calafell, M. et al. (2014)** | Treatment | 12 | Outpatients with schizophrenia or schizo-affective disorder (DSM-IV diagnosis).  (7 = male, 5 = female) | Laptop with 3D glasses and headphones. Soskitrain programme. | Used a VR integrated programme as a technique to improve social cognition and improve the daily lives of people with schizophrenia. Hypothesised that the intervention would lead to improvements in social cognition and performance, as well as generalisation of the learned responses into the everyday lives of patients. | Participants reported high degree of treatment satisfaction, acceptance and perceived benefits. Significant decrease in negative symptomology, psychopathology and social improvement following treatment. Improvement in social skill mastery following treatment. These gains were maintained at 4-month follow up, however initial post-treatment improvements in social discomfort and social anxiety were not maintained. |
| **43. Ruse et al (2014)** | Assessment | 105 | 51 people with schizophrenia, 54 non-clinical controls | Unclear but may be HMD | Aim to use VR to assess functional capacity: Virtual Reality Functional Capacity Assessment Tool (VRFCAT). The authors aimed for a better assessment of real-world cognitive functioning. | The authors argue the computerised tool shows promise to measure functional abilities. |
| **44. Keefe et al (2016)** | Assessment | 333 | 167 patients with schizophrenia, 166 non-clinical controls | Unclear but may be HMD | Aim to use VR to assess functional capacity: Virtual Reality Functional Capacity Assessment Tool (VRFCAT). The authors aimed for a better assessment of real-world cognitive functioning. | It is argued that the VRFCAT provides a reliable and sensitive measure of functional capacity. |

**REFERENCES**

Atherton, S., Antley, A., Evans, N., Cernis, E., Lister, R., Dunn, G., … Freeman, D. (2016). Self-Confidence and Paranoia: An Experimental Study Using an Immersive Virtual Reality Social Situation. *Behavioural and Cognitive Psychotherapy*, *44*(1), 56–64. http://doi.org/10.1017/S1352465814000496

Brinkman, W.-P., Veling, W., Dorrestijn, E., Sandino, G., Vakili, V., & van der Gaag, M. (2011). Virtual reality to study responses to social environmental stressors in individuals with and without psychosis. *Studies in Health Technology and Informatics*, *167*, 86–91. Retrieved from http://www.ncbi.nlm.nih.gov/pubmed/21685647

Broome, M. R., Zányi, E., Hamborg, T., Selmanovic, E., Czanner, S., Birchwood, M., … Singh, S. P. (2013). A high-fidelity virtual environment for the study of paranoia. *Schizophrenia Research and Treatment*, *2013*, 538185. http://doi.org/10.1155/2013/538185

Chan, C. L. F., Ngai, E. K. Y., Leung, P. K. H., & Wong, S. (2010). Effect of the adapted Virtual Reality cognitive training program among Chinese older adults with chronic schizophrenia: a pilot study. *International Journal of Geriatric Psychiatry*, *25*(6), 643–9. http://doi.org/10.1002/gps.2403

da Costa, R. M. E. M., & de Carvalho, L. A. V. (2004). The acceptance of virtual reality devices for cognitive rehabilitation: a report of positive results with schizophrenia. *Computer Methods and Programs in Biomedicine*, *73*(3), 173–82. http://doi.org/10.1016/S0169-2607(03)00066-X

Fornells-Ambrojo, M., Barker, C., Swapp, D., Slater, M., Antley, A., & Freeman, D. (2008). Virtual reality and persecutory delusions: safety and feasibility. *Schizophrenia Research*, *104*(1-3), 228–36. http://doi.org/10.1016/j.schres.2008.05.013

Fornells-Ambrojo, M., Elenbaas, M., Barker, C., Swapp, D., Navarro, X., Rovira, A., … Slater, M. (2016). Hypersensitivity to Contingent Behavior in Paranoia: A New Virtual Reality Paradigm. *The Journal of Nervous and Mental Disease*, *204*(2), 148–52. http://doi.org/10.1097/NMD.0000000000000414

Fornells-Ambrojo, M., Freeman, D., Slater, M., Swapp, D., Antley, A., & Barker, C. (2015). How do people with persecutory delusions evaluate threat in a controlled social environment? A qualitative study using virtual reality. *Behavioural and Cognitive Psychotherapy*, *43*(1), 89–107. http://doi.org/10.1017/S1352465813000830

Freeman, D., Gittins, M., Pugh, K., Antley, A., Slater, M., & Dunn, G. (2008). What makes one person paranoid and another person anxious? The differential prediction of social anxiety and persecutory ideation in an experimental situation. *Psychological Medicine*, *38*(8), 1121–32. http://doi.org/10.1017/S0033291708003589

Freeman, D., Antley, A., Ehlers, A., Dunn, G., Thompson, C., Vorontsova, N., … Slater, M. (2014). The use of immersive virtual reality (VR) to predict the occurrence 6 months later of paranoid thinking and posttraumatic stress symptoms assessed by self-report and interviewer methods: a study of individuals who have been physically assaulted. *Psychological Assessment*, *26*(3), 841–7. http://doi.org/10.1037/a0036240

Freeman, D., Bradley, J., Antley, A., Bourke, E., DeWeever, N., Evans, N., … Clark, D. M. (2016). Virtual reality in the treatment of persecutory delusions: randomised controlled experimental study testing how to reduce delusional conviction. *The British Journal of Psychiatry : The Journal of Mental Science*, *209*(1), 62–7. http://doi.org/10.1192/bjp.bp.115.176438

Freeman, D., Dunn, G., Murray, R. M., Evans, N., Lister, R., Antley, A., … Morrison, P. D. (2015). How cannabis causes paranoia: using the intravenous administration of ∆9-tetrahydrocannabinol (THC) to identify key cognitive mechanisms leading to paranoia. *Schizophrenia Bulletin*, *41*(2), 391–9. http://doi.org/10.1093/schbul/sbu098

Freeman, D., Evans, N., Lister, R., Antley, A., Dunn, G., & Slater, M. (2014). Height, social comparison, and paranoia: an immersive virtual reality experimental study. *Psychiatry Research*, *218*(3), 348–52. http://doi.org/10.1016/j.psychres.2013.12.014

Freeman, D., Garety, P. A., Bebbington, P., Slater, M., Kuipers, E., Fowler, D., … Dunn, G. (2005). The psychology of persecutory ideation II: a virtual reality experimental study. *The Journal of Nervous and Mental Disease*, *193*(5), 309–15. Retrieved from http://www.ncbi.nlm.nih.gov/pubmed/15870614

Freeman, D., Pugh, K., Antley, A., Slater, M., Bebbington, P., Gittins, M., … Garety, P. (2008). Virtual reality study of paranoid thinking in the general population. *The British Journal of Psychiatry : The Journal of Mental Science*, *192*(4), 258–63. http://doi.org/10.1192/bjp.bp.107.044677

Freeman, D., Pugh, K., Green, C., Valmaggia, L., Dunn, G., & Garety, P. (2007). A measure of state persecutory ideation for experimental studies. *The Journal of Nervous and Mental Disease*, *195*(9), 781–4. http://doi.org/10.1097/NMD.0b013e318145a0a9

Freeman, D., Pugh, K., Vorontsova, N., Antley, A., & Slater, M. (2010). Testing the continuum of delusional beliefs: an experimental study using virtual reality. *Journal of Abnormal Psychology*, *119*(1), 83–92. http://doi.org/10.1037/a0017514

Freeman, D., Slater, M., Bebbington, P. E., Garety, P. A., Kuipers, E., Fowler, D., … Vinayagamoorthy, V. (2003). Can virtual reality be used to investigate persecutory ideation? *The Journal of Nervous and Mental Disease*, *191*(8), 509–14. http://doi.org/10.1097/01.nmd.0000082212.83842.fe

Han, K., Shin, J., Yoon, S. Y., Jang, D.-P., & Kim, J.-J. (2014). Deficient gaze pattern during virtual multiparty conversation in patients with schizophrenia. *Computers in Biology and Medicine*, *49*, 60–6. http://doi.org/10.1016/j.compbiomed.2014.03.012

Han, K., Young Kim, I., & Kim, J.-J. (2012). Assessment of cognitive flexibility in real life using virtual reality: a comparison of healthy individuals and schizophrenia patients. *Computers in Biology and Medicine*, *42*(8), 841–7. http://doi.org/10.1016/j.compbiomed.2012.06.007

Keefe, R. S. E., Davis, V. G., Atkins, A. S., Vaughan, A., Patterson, T., Narasimhan, M., & Harvey, P. D. (2016). Validation of a Computerized test of Functional Capacity. Schizophrenia Research, 175, 90–96. http://doi.org/10.1016/j.schres.2016.03.038

Kim, K., Kim, J.-J., Kim, J., Park, D.-E., Jang, H. J., Ku, J., … Kim, S. I. (2007). Characteristics of social perception assessed in schizophrenia using virtual reality. *Cyberpsychology & Behavior : The Impact of the Internet, Multimedia and Virtual Reality on Behavior and Society*, *10*(2), 215–9. http://doi.org/10.1089/cpb.2006.9966

Ku, J., Cho, W., Kim, J.-J., Peled, A., Wiederhold, B. K., Wiederhold, M. D., … Kim, S. I. (2003). A virtual environment for investigating schizophrenic patients’ characteristics: assessment of cognitive and navigation ability. *Cyberpsychology & Behavior : The Impact of the Internet, Multimedia and Virtual Reality on Behavior and Society*, *6*(4), 397–404. http://doi.org/10.1089/109493103322278781

Ku, J., Han, K., Lee, H. R., Jang, H. J., Kim, K. U., Park, S. H., … Kim, S. I. (2007). VR-based conversation training program for patients with schizophrenia: a preliminary clinical trial. *Cyberpsychology & Behavior : The Impact of the Internet, Multimedia and Virtual Reality on Behavior and Society*, *10*(4), 567–74. http://doi.org/10.1089/cpb.2007.9989

Ku, J., Jang, H. J., Kim, K. U., Park, S. H., Kim, J. J., Kim, C. H., … Kim, S. I. (2006). Pilot study for assessing the behaviors of patients with schizophrenia towards a virtual avatar. *Cyberpsychology & Behavior : The Impact of the Internet, Multimedia and Virtual Reality on Behavior and Society*, *9*(5), 531–9. http://doi.org/10.1089/cpb.2006.9.531

Park, I. H., Kim, J.-J., Ku, J., Jang, H. J., Park, S.-H., Kim, C.-H., … Kim, S. I. (2009). Characteristics of social anxiety from virtual interpersonal interactions in patients with schizophrenia. *Psychiatry*, *72*(1), 79–93. http://doi.org/10.1521/psyc.2009.72.1.79

Park, K.-M., Ku, J., Choi, S.-H., Jang, H.-J., Park, J.-Y., Kim, S. I., & Kim, J.-J. (2011). A virtual reality application in role-plays of social skills training for schizophrenia: a randomized, controlled trial. *Psychiatry Research*, *189*(2), 166–72. http://doi.org/10.1016/j.psychres.2011.04.003

Park, K.-M., Ku, J., Park, I.-H., Park, J.-Y., Kim, S. I., & Kim, J.-J. (2009). Improvement in social competence in patients with schizophrenia: a pilot study using a performance-based measure using virtual reality. *Human Psychopharmacology*, *24*(8), 619–27. http://doi.org/10.1002/hup.1071

Park, S.-H., Ku, J., Kim, J.-J., Jang, H. J., Kim, S. Y., Kim, S. H., … Kim, S. I. (2009). Increased personal space of patients with schizophrenia in a virtual social environment. *Psychiatry Research*, *169*(3), 197–202. http://doi.org/10.1016/j.psychres.2008.06.039

Rus-Calafell, M., Gutiérrez-Maldonado, J., & Ribas-Sabaté, J. (2012). Improving social behaviour in schizophrenia patients using an integrated virtual reality programme: a case study. *Studies in Health Technology and Informatics*, *181*, 283–6. Retrieved from http://www.ncbi.nlm.nih.gov/pubmed/22954872

Rus-Calafell, M., Gutiérrez-Maldonado, J., & Ribas-Sabaté, J. (2014). A virtual reality-integrated program for improving social skills in patients with schizophrenia: a pilot study. *Journal of Behavior Therapy and Experimental Psychiatry*, *45*(1), 81–9. http://doi.org/10.1016/j.jbtep.2013.09.002

Rus-Calafell, M., Gutiérrez-Maldonado, J., & Ribas-Sabaté, J. (2013). Neurocognition, presence and acceptance of a VR programme for psychotic patients: a correlational study. *Studies in Health Technology and Informatics*, *191*, 141–5. Retrieved from http://www.ncbi.nlm.nih.gov/pubmed/23792861

Salgado-Pineda, P., Landin-Romero, R., Portillo, F., Bosque, C., Pomes, A., Spanlang, B., … McKenna, P. J. (2016). Examining hippocampal function in schizophrenia using a virtual reality spatial navigation task. *Schizophrenia Research*, *172*(1-3), 86–93. http://doi.org/10.1016/j.schres.2016.02.033

Shaikh, M., Ellett, L., Dutt, A., Day, F., Laing, J., Kroll, J., … Valmaggia, L. R. (2016). Perceived ethnic discrimination and persecutory paranoia in individuals at ultra-high risk for psychosis. *Psychiatry Research*, *241*, 309–14. http://doi.org/10.1016/j.psychres.2016.05.006

Siemerkus, J., Irle, E., Schmidt-Samoa, C., Dechent, P., & Weniger, G. (2012). Egocentric spatial learning in schizophrenia investigated with functional magnetic resonance imaging., *1*(1). http://doi.org/10.1016/j.nicl.2012.10.004

Sorkin, A., Weinshall, D., Modai, I., & Peled, A. (2006). Improving the accuracy of the diagnosis of schizophrenia by means of virtual reality. *The American Journal of Psychiatry*, *163*(3), 512–20. http://doi.org/10.1176/appi.ajp.163.3.512

Sorkin, A., Weinshall, D., & Peled, A. (2008). The distortion of reality perception in schizophrenia patients, as measured in Virtual Reality. *Studies in Health Technology and Informatics*, *132*, 475–80. Retrieved from http://www.ncbi.nlm.nih.gov/pubmed/18391348

Stinson, K., Valmaggia, L. R., Antley, A., Slater, M., & Freeman, D. (2010). Cognitive triggers of auditory hallucinations: an experimental investigation. *Journal of Behavior Therapy and Experimental Psychiatry*, *41*(3), 179–84. http://doi.org/10.1016/j.jbtep.2009.12.003

Valmaggia, L. R., Day, F. L., Kroll, J., Laing, J., Byrne, M., Fusar-Poli, P., & McGuire, P. (2015). Bullying victimisation and paranoid ideation in people at ultra high risk for psychosis. *Schizophrenia Research*, *168*(1-2), 68–73. http://doi.org/10.1016/j.schres.2015.08.029

Valmaggia, L. R., Day, F., Garety, P., Freeman, D., Antley, A., Slater, M., … McGuire, P. (2015). Social defeat predicts paranoid appraisals in people at high risk for psychosis. *Schizophrenia Research*, *168*(1-2), 16–22. http://doi.org/10.1016/j.schres.2015.07.050

Valmaggia, L. R., Freeman, D., Green, C., Garety, P., Swapp, D., Antley, A., … McGuire, P. K. (2007). Virtual reality and paranoid ideations in people with an “at-risk mental state” for psychosis. *The British Journal of Psychiatry. Supplement*, *51*, s63–8. http://doi.org/10.1192/bjp.191.51.s63

Veling, W., Brinkman, W.-P., Dorrestijn, E., & van der Gaag, M. (2014). Virtual reality experiments linking social environment and psychosis: a pilot study. *Cyberpsychology, Behavior and Social Networking*, *17*(3), 191–5. http://doi.org/10.1089/cyber.2012.0497

Veling, W., Pot-Kolder, R., Counotte, J., van Os, J., & van der Gaag, M. (2016). Environmental Social Stress, Paranoia and Psychosis Liability: A Virtual Reality Study. *Schizophrenia Bulletin*. http://doi.org/10.1093/schbul/sbw031
