# Supplementary material for: Virtual reality in the assessment, understanding, and treatment of mental health disorders
Source: Psychol Med. 2017 Mar 22;47(14):2393–400. doi: 10.1017/S003329171700040X (PMC5964457; doi:10.1017/S003329171700040X)
Supplement: Supplementary file 1 [file S003329171700040Xsup001.zip › S003329171700040Xsup004.doc]

**Substance use**

**Supplementary Fig. S4.** PRISMA 2009 Flow Diagram

**Screening**

**Included**

**Eligibility**

**Identification**

Records identified through database searching
(n =	101)

Additional records identified through other sources
(n = 0 )

Records after duplicates removed
(n = 101 )

Records screened
(n = 101 )

Records excluded
(n = 70 )

Full-text articles assessed for eligibility
(n = 31 )

Studies included in qualitative synthesis
(n = 22 )

Full-text articles excluded, with reasons
(n = 9)

Not immersive technologies=8

Did not relate to mental health =1

**Supplementary Table S4.** *Substance use studies*

| Authors | Category | N | Participant characteristics | Virtual reality type | Aims | Conclusions |
| --- | --- | --- | --- | --- | --- | --- |
| 1. Lister et al (2016) | Theory | 121 | Individuals who had reported gambling | HMD showing an interactive casino | This study assessed gambling goals before testing gambling performance in a rigged VR casino. | Gambling goals and problem gambling severity predicted chasing behaviour. |
| 2. Pericot-Valverde et al (2015) | Treatment | 41 | Treatment seeking smokers | HMD showing smoking-related cues | This was a case series seeing who benefited from VR cue exposure treatment, once a week over five weeks. | A number of individual characteristics (e.g. younger age, delay discounting) predicted potential benefit, but this was not a controlled study so could not tell what was due to the VR CET. |
| 3. Thompson-Lake et al (2015) | Assessment | 36 | Nicotine dependent cigarette smokers | HMD | After being deprived of cigarettes, whether VR cigarette cues led to cravings. | Cravings occurred to smoking compared to non-smoking cues. |
| 4. Freeman et al (2015) | Theory | 121 | People with paranoid thoughts and who had taken cannabis | HMD displaying a neutral social situation | In a randomised controlled test, participants were tested before and after receiving THC in order to test the psychological effects. | THC led to increases in negative affect, paranoia, and anomalous experiences and a decrease in working memory performance. |
| 5. Pericot-Valverde et al (2014) | Treatment | 48 | Treatment seeking smokers | HMD | VR used for cue exposure treatment, involving social interactions where people were smoking or offering cigarettes, talking, dancing, having coffee, and drinking alcoholic beverages. | In this uncontrolled study, VR CET led to reduction in cravings. |
| 6. Hadley et al (2014) | Assessment | 42 | Adolescents | HMD displaying party scenes. | Testing whether VR could elicit physiological arousal to substance use and sexual risk-taking cues. | The cues did produce physiological arousal. |
| 7. Acker & MacKillop (2014) | Assessment | 47 | Regular smokers | HMD | Tobacco cues were presented in VR. | Carving occurred in response to the VR cues. Behavioral economic indices of demand for tobacco also increased. |
| 8. Traylor et al (2011) | Assessment | 21 | Nicotine dependent daily drinkers | HMD | The pilot study tested nicotine and alcohol cue reactivity across different VR scenes with smoking cues. | VR elicited cravings, which varied by participant characteristics. |
| 9. Lee et al (2008) | Assessment | 28 | 14 male patients with alcohol dependence and 14 age-matched social drinkers | HMD displaying a virtual pub | Alcohol and social pressure were varied in the conditions. | VR elicited alcohol cravings, especially in the alcohol dependence group regardless of social pressure. |
| 10. Girard et al (2009) | Treatment | 91 | Regular smokers | HMD | Randomised to crushing virtual cigarettes or grasping virtual balls. All participants also took part in a psychosocial programme for reducing smoking. | Crushing cigarettes was associated with better outcomes. |
| 11. Bordnick et al (2009). | Assessment | 20 | Current cannabis smokers | HMD with audio, visual, olfactory, and vibrotactile sensory stimuli. | Tested whether cannabis cues can trigger cravings. | The VR cues triggered cannabis cravings. |
| 12. Lee et al (2009) | Treatment | 38 | Alcohol dependent males. | The virtual reality therapy (VRT) consisted of scenes that were associated with relaxation, virtual alcohol cues and then an aversive stimulation. Projectors and googles used. | 20 individuals had 10 VRT sessions, and 18 had CBT. This was not randomised. | The individuals who had the VR intervention had decreased alcohol cravings compared to the other patients. |
| 13. Cho et al (2008) | Assessment | 10 | No history of alcohol-related disease. | HMD, varying alcohol cues and social pressure. | This tested the effects of VR alcohol cues and avatar pressure to increase alcohol cues. | Alcohol cues and social pressure increased alcohol cravings. |
| 14. Bordnick et al (2008) | Assessment | 40 | Non-treatment seeking drinkers | HMD plus cue scent system. | VR alcohol cues were compared to VR neutral cues. | Alcohol related cues produced cravings |
| 15. Carter et al (2008) | Assessment | 22 | Smokers | HMD presenting a neutral scene or party scene with smoking cues. Visual, olfactory, and auditory cues were used. | Compared craving in neutral and smoking cue conditions. | Cigarette cues produced cravings. |
| 16. Lee et al (2007) | Treatment | 8 | Members of alcoholics anonymous group. | Large screen and projector system. | Eight sessions of VR cue-exposure therapy. | Alcohol urges decreased but there was no control condition. |
| 17. Saladin et al (2006) | Assessment | 11 | Crack cocaine dependent individuals | HMD | Seven cocaine-related scenes were used and a neutral environment. | Craving increased in the cocaine-related scenes. |
| 18. Bordnick et al (2005) | Assessment | 10 | Smokers | HMD | Smokers exposed to neutral and smoking related VR  environments. | Smoking cues elicited urges. |
| 19. Bordnick et al (2005). | Assessment | 1 | Smoker | HMD | Case study of VR exposure to smoking cues | Cravings occurred to the VR smoking cues. |
| 20. Bordnick et al (2004) | Assessment | 13 | Smokers | HMD | VR smoking related and neutral scenes presented. | Cravings occurred to smoking cues. |
| 21. Lee et al (2003) | Assessment | 22 | Smokers | HMD | Carving across smoking related VR and pictures compared. | VR was better at eliciting cravings than pictures. |
| 22. Kuntze et al (2001). | Assessment | 15 | Males, opioid dependent. | HMD | VR cue exposure compared with pictures compared with neutral stimuli. | VR elicited the greater drug cravings. |

**REFERENCES**

Acker J, MacKillop J. (2013). Behavioral economic analysis of cue-elicited craving for tobacco: a virtual reality study. Nicotine Tob Res.;15:1409-16. doi: 10.1093/ntr/nts341.

Bordnick PS, Copp HL, Traylor A, Graap KM, Carter BL, Walton A, Ferrer M. (2009). Reactivity to cannabis cues in virtual reality environments J Psychoactive Drugs. ;41(2):105-12.

Bordnick PS, Traylor A, Copp HL, Graap KM, Carter B, Ferrer M, Walton AP (2008). Assessing reactivity to virtual reality alcohol based cues. Addict Behav. 2008 Jun;33(6):743-56. doi: 10.1016/j.addbeh.2007.12.010. Epub 2008 Jan 2.

Bordnick, P.S., Graap, K.M., Copp, H.L., Brooks, J., Ferrer, M. (2005). Virtual reality cue reactivity assessment in cigarette smokers. Cyberpsychol Behav. ;8, 487-492.

Bordnick PS, Traylor AC, Graap KM, Copp HL, Brooks J. (2005). Virtual reality cue reactivity assessment: a case study in a teen smoker. Appl Psychophysiol Biofeedback. ;30(3):187-93.

Bordnick PS, Graap KM, Copp H, Brooks J, Ferrer M, Logue B. (2004). Utilizing virtual reality to standardize nicotine craving research: a pilot study. Addict Behav. ;29(9):1889-94.

Carter BL1, Bordnick P, Traylor A, Day SX, Paris M. (2008). Location and longing: the nicotine craving experience in virtual reality. Drug Alcohol Depend.;95(1-2):73-80. doi: 10.1016/j.drugalcdep.2007.12.010. Epub 2008 Feb 19.

Cho S, Ku J, Park J, Han K, Lee H, Choi YK, Jung YC, Namkoong K, Kim JJ, Kim IY, Kim SI, Shen DF. (2008). Development and verification of an alcohol craving-induction tool using virtual reality: craving characteristics in social pressure situation. Cyberpsychol Behav.;11(3):302-9. doi: 10.1089/cpb.2007.0149.

Freeman, D., Dunn, G., Murray, R., Evans, N., Lister, R., Antley, A., Slater, M., Godlewska, B., Cornish, R., Williams, J., Di Simplicio, M., Igoumenou, A., Brenneisen, R., Tunbridge, E., Harrison, P., Harmer, C., Cowen, P., Morrison, P. (2015). How cannabis causes paranoia: Using the intravenous administration of ∆9-tetrahydrocannabinol (THC) to identify key cognitive mechanisms leading to paranoia. Schizophrenia Bulletin, 41, 391-399.

Girard, B., Turcotte, V., Bouchard, S., & Girard, B. (2009). Crushing virtual cigarettes reduces tobacco addiction and treatment discontinuation. Cyberpsychol Behav. ;12, 477-483.

Hadley W, Houck CD, Barker DH, Garcia AM, Spitalnick JS, Curtis V, Roye S, Brown LK. (2014). Eliciting affect via immersive virtual reality: a tool for adolescent risk reduction. J Pediatr Psychol.;39(3):358-68. doi: 10.1093/jpepsy/jst092. Epub 2013 Dec 23.

Kuntze MF, Stoermer R, Mager R, Roessler A, Mueller-Spahn F, Bullinger AH. (2001). Immersive virtual environments in cue exposure. Cyberpsychol Behav. 4(4):497-501.

Lee, J.S., Namkoong, K., Ku, J., Cho, S., Park, J.Y., Choi, Y.K., Kim, J.J., Kim, I.Y., Kim, S.I., Jung, Y.C. (2008). Social pressure-induced craving in patients with alcohol dependence: application of virtual reality to coping skill training. Psychiatry Investig.;5, 239-243.

Lee SH, Han DH, Oh S, Lyoo IK, Lee YS, Renshaw PF, Lukas SE. (2009). Quantitative electroencephalographic (qEEG) correlates of craving during virtual reality therapy in alcohol-dependent patients.Pharmacol Biochem Behav.;91(3):393-7. doi: 10.1016/j.pbb.2008.08.014. Epub 2008 Aug 17.

Lee JH, Kwon H, Choi J, Yang BH. (2007). Cue-exposure therapy to decrease alcohol craving in virtual environment. Cyberpsychol Behav. ;10(5):617-23.

Lee JH, Ku J, Kim K, Kim B, Kim IY, Yang BH, Kim SH, Wiederhold BK, Wiederhold MD, Park DW, Lim Y, Kim SI. (2003). Experimental application of virtual reality for nicotine craving through cue exposure.Cyberpsychol Behav. ;6(3):275-80.

Lister JJ, Nower L, Wohl MJ. (2016). Gambling goals predict chasing behavior during slot machine play. Addict Behav.;62:129-34. doi: 10.1016/j.addbeh.2016.06.018. Epub 2016 Jun 14.

Pericot-Valverde I, García-Rodríguez O, Gutiérrez-Maldonado J, Secades-Villa R. (2015). Individual variables related to craving reduction in cue exposure treatment. Addict Behav.;49:59-63. doi: 10.1016/j.addbeh.2015.05.013. Epub 2015 May 23.

Pericot-Valverde, I., Secades-Villa, R., Gutiérrez-Maldonado, J. & García-Rodríguez, O. (2014). Effects of Systematic Cue Exposure Through Virtual Reality on Cigarette Craving. Nicotine Tob Res, 16, 1470-1477.

Saladin, M., Brady, K., Graap, K., & Rothbaum, B. (2006). A preliminary report on the use of virtual reality technology to elicit craving and cue reactivity in cocaine dependent individuals. Addictive Behvaiors, 31, 1881-1894.

Thompson-Lake DG, Cooper KN, Mahoney JJ 3rd, Bordnick PS, Salas R, Kosten TR, Dani JA, De La Garza R 2nd. (2015). Withdrawal Symptoms and Nicotine Dependence Severity Predict Virtual Reality Craving in Cigarette-Deprived Smokers. Nicotine Tob Res.;17(7):796-802. doi: 10.1093/ntr/ntu245.

Traylor AC, Parrish DE, Copp HL, Bordnick PS. (2011). Using virtual reality to investigate complex and contextual cue reactivity in nicotine dependent problem drinkers. Addict Behav. ;36(11):1068-75. doi: 10.1016/j.addbeh.2011.06.014.
