# Supplementary material for: Virtual reality in the assessment, understanding, and treatment of mental health disorders
Source: Psychol Med. 2017 Mar 22;47(14):2393–400. doi: 10.1017/S003329171700040X (PMC5964457; doi:10.1017/S003329171700040X)
Supplement: Supplementary file 1 [file S003329171700040Xsup001.zip › S003329171700040Xsup005.doc]

**Eating disorders**

**Supplementary Fig. S5.** PRISMA 2009 Flow Diagram

**Screening**

**Included**

**Eligibility**

**Identification**

Records identified through database searching
(n =	67)

Additional records identified through other sources
(n = 0 )

Records after duplicates removed
(n = 66 )

Records screened
(n = 66 )

Records excluded
(n = 43 )

Full-text articles assessed for eligibility
(n = 23 )

Studies included in qualitative synthesis
(n = 18 )

Full-text articles excluded, with reasons
(n = 5 )

Not immersive technologies=5

**Supplementary Table S5.** *Eating disorders studies*

| Authors | Category | N | Participant characteristics | Virtual reality type | Aims | Conclusions |
| --- | --- | --- | --- | --- | --- | --- |
| 1. Gutiérrez-Maldonado et al (2016) | Treatment | 113 | Undergraduates | One session cue exposure session via HMD or laptop 3D screen | The aim was to see whether exposure to food-related VR environments reduced craving and whether level of immersion (by equipment) made a difference. | Both systems reduced food cravings (with the HMD to a greater degree but not statistically) but there was no control and this was a non-clinical group. |
| 2. Keizer et al (2016). | Treatment | 59 | 30 patients with anorexia nervosa; 29 non-clinical controls | HMD | The researchers wanted to investigate whether it was possible to alter body image in patients with eating disorders by experiencing in VR a body with normal BMI. | After VR, the patients did reduce their misjudgements of their own body size. |
| 3. Serino et al (2015) | Theory | 44 | 22 women with eating disorders and 22 non-clinical controls | CAVE | A VR neuropsychological test was used concerning a spatial memory task. | The eating disorders group had poorer performance in the VR task. |
| 4. Purvis et al (2015) | Assessment | 78 | 38 females with weight and shape concerns, 40 women without weight and shape concerns. | HMD | The researchers wanted to test whether VR can be used to assess body image disturbance by showing different VR scenes with people of different BMIs. | After the VR environments, body image dissatisfaction was higher in the women who reported such concerns. |
| 5. Mountford et al (2016) | Assessment | 41 | 21 Non-clinical dieting group, 20 non-dieting | HMD | The authors wanted to see whether body image concerns can be elicited in VR. Participants went on a bus journey with avatars of “normal body weight and appearance”. | Body image disturbance was not elevated by the VR environment, though the environment was not specifically built to trigger such concerns. |
| 6. Ferrer-Garcia et al (2015). | Assessment | 87 | Female students | Laptop with polarized glasses. | VR high and low calorie foods were displayed in the presence or absence of avatars. | Higher cravings were found for the higher calorie VR foods. |
| 7. Ferrer-Garcia et al (2013) | Assessment | 83 | Students | Computer and polarized glasses. | Participants were exposed in random order to: a low-calorie kitchen, a high-calorie kitchen, a low-calorie restaurant, and a high-calorie restaurant. | Exposure to high calorie food produced the greatest cravings. |
| 8. Cesa et al (2013) | Treatment | 90 | Women with obesity and binge eating disorders. | Unclear which VR system used. | Participants randomly assigned to VR enhanced CBT or CBT or control. All patients also had the standard inpatient treatment programme. | The data indicated that the VR treatment may help in weight loss. |
| 9. Marco et al (2013). | Treatment | 34 | Female patients with an eating disorder (bulimia nervosa, eating disorder NOS, anorexia nervosa) | HMD | Patients were randomly assigned to CBT or CBT with a VR component concerning body image. | The analysis was not intention to treat. The VR element may have helped increase body image. |
| 10. Gutiérrez-Maldonado et al (2010). | Assessment | 193 | 85 patients with eating disorders, 108 students. | Unclear which VR system used. | Different virtual environments were used including no food, high-calorie food, and low calorie food and with other present or not. | The patients showed higher levels of body image distortion and dissatisfaction after being in the virtual environments. The presence of avatars did not moderate the results. |
| 11. Gorini et al (2010) | Assessment | 30 | 10 patients with anorexia nervosa, 10 patients with bulimia nervosa, and 10 non-clinical controls. | HMD | Responses to real food, pictures of food, and VR food were compared. | Real and virtual food induced comparable emotional responses. |
| 12. Gutiérrez-Maldonado et al (2006). | Assessment | 30 | Female patients with eating disorders | It is unclear how the environments were displayed. | Six different VR environments which were neutral, low calorie or high calorie or body image related were presented. | The VR environments triggered the expected emotional reactions in the patients. |
| 13. Riva et al (2003) | Treatment | 36 | Females with binge eating disorders | 10 biweekly VR sessions were included in CBT. Uncertain how VR displayed but probably (from previous reports) a HMD. | Patients were randomised to CBT, CBT with VR, or control. All patients also had nutritional groups. | The VR element looked to have enhanced CBT in reducing binge frequency. |
| 14. Riva et al (2002) | Treatment | 20 | Females with binge eating disorders | HMD | Patients randomised to nutritional groups or CBT treatment including an extensive VR element. | Binge eating did not differ between the two groups. The VR CBT intervention improved body satisfaction to a greater degree. |
| 15. Riva et al (2001) | Treatment | 28 | Female patients with obesity | HMD | The aim was to improve body image. Patients were randomly assigned to a nutritional group or a seven session VR treatment. | The VR treatment showed evidence of improving body image. |
| 16. Riva et al (1998) | Treatment | 1 | A patient with anorexia nervosa | HMD | A case study VR treatment is described (combined with other treatments). | Improvements were shown on measures. |
| 17. Perpiñá et al (1999) | Treatment | 13 | Outpatients with eating disorders | HMD | Patients were randomly allocated to ‘standard body image treatment’ or a VR body image treatment. | The VR treatment showed some better improvements for body image specifically. |
| 18. Riva (1998) | Treatment | 119 | Non-clinical participants | HMD | The paper gives two preliminary reports on the development of a VR treatment for body image dissatisfaction. | The treatment was safe to use and showed evidence of improving body image in this non-clinical study. |

**REFERENCES**

Cesa, G.L., Manzoni, G.M., Bacchetta, M., Castelnuovo, G., Conti, S., Gaggioli, A., Mantovani, F., Molinari, E., Cárdenas-López, G., & Riva, G. V(2013). Virtual reality for enhancing the cognitive behavioral treatment of obesity with binge eating disorder: randomized controlled study with one-year follow-up. J Med Internet Res.;15(6):e113. doi: 10.2196/jmir.2441.

Ferrer-García M, Gutiérrez-Maldonado J, Pla J. (2013). Cue-elicited anxiety and craving for food using virtual reality scenarios. Stud Health Technol Inform. ;191:105-109.

Ferrer-Garcia, M., Gutierrez-Maldonado, J., Treasure, J., Vilalta-Abella, F. (2015). Craving for Food in Virtual Reality Scenarios in Non-Clinical Sample: Analysis of its Relationship with Body Mass Index and Eating Disorder Symptoms. Eur Eat Disord Rev.;23:371-8. doi: 10.1002/erv.2375.

Gorini, A., Griez, E., Petrova, A., & Riva, G. (2010). Assessment of the emotional responses produced by exposure to real food, virtual food and photographs of food in patients affected by eating disorders. Ann Gen Psychiatry; 9:30. doi: 10.1186/1744-859X-9-30.

Gutiérrez-Maldonado J, Ferrer-García M, Caqueo-Urízar A, Moreno E. (2010). Body image in eating disorders: the influence of exposure to virtual-reality environments. Cyberpsychol Behav Soc Netw. ;13:521-31. doi: 10.1089/cyber.2009.0301.

Gutiérrez-Maldonado J1, Ferrer-García M, Caqueo-Urízar A, Letosa-Porta A. (2006). Assessment of emotional reactivity produced by exposure to virtual environments in patients with eating disorders. Cyberpsychol Behav. ;9:507-13.

Gutiérrez-Maldonado J, Pla-Sanjuanelo J, Ferrer-García M. (2016). Cue-exposure software for the treatment of bulimia nervosa and binge eating disorder. Psicothema;28(4):363-369.

Keizer A, van Elburg A, Helms R, Dijkerman HC. (2016). A Virtual Reality Full Body Illusion Improves Body Image Disturbance in Anorexia Nervosa. PLoS One. 2016 Oct 6;11(10):e0163921. doi: 10.1371/journal.pone.0163921. eCollection 2016.

Marco, J.H., Perpiñá, C., & Botella, C. (2013). Effectiveness of cognitive behavioral therapy supported by virtual reality in the treatment of body image in eating disorders: one year follow-up. Psychiatry Res.;209:619-25. doi: 10.1016/j.psychres.2013.02.023.

Mountford VA, Tchanturia K, Valmaggia L. (2016). "What Are You Thinking When You Look at Me?" A Pilot Study of the Use of Virtual Reality in Body Image. Cyberpsychol Behav Soc Netw.:93-9. doi: 10.1089/cyber.2015.0169.

Perpiñá C, Botella C, Baños R, Marco H, Alcañiz M, Quero S. (1999). Body image and virtual reality in eating disorders: is exposure to virtual reality more effective than the classical body image treatment? Cyberpsychol Behav., 2, 149-155. doi: 10.1089/cpb.1999.2.149.

Purvis CK, Jones M, Bailey JO, Bailenson J, Taylor CB. (2015). Developing a Novel Measure of Body Satisfaction Using Virtual Reality. PLoS One.;10(10):e0140158. doi: 10.1371/journal.pone.0140158.

Riva, G. (1998). Virtual Environment for Body Image Modification: Virtual Reality System for the Treatment of Body Image Disturbances. Computers in Human Behavior, 14, 477-490.

Riva G, Bacchetta M, Baruffi M, Rinaldi S, Molinari E. (1998). Experiential cognitive therapy in anorexia nervosa. Eat Weight Disord.;3:141-150.

Riva G, Bacchetta M, Baruffi M, Molinari E. (2001). Virtual reality-based multidimensional therapy for the treatment of body image disturbances in obesity: a controlled study. Cyberpsychol Behav., 4:511-526.

Riva G, Bacchetta M, Baruffi M, Molinari E. (2002). Virtual-reality-based multidimensional therapy for the treatment of body image disturbances in binge eating disorders: a preliminary controlled study. IEEE Trans Inf Technol Biomed.;6(3):224-34

Riva, G., Bacchetta, M., Cesa, G., Conti, S., Molinari, E. (2003). Six-month follow-up of in-patient experiential cognitive therapy for binge eating disorders. Cyberpsychol Behav.;6:251-8.

Serino S, Dakanalis A, Gaudio S, Carrà G, Cipresso P, Clerici M, Riva G. (2015). Out of body, out of space: Impaired reference frame processing in eating disorders. Psychiatry Res.;230:732-4. doi: 10.1016/j.psychres.2015.10.025.
