# Supplementary material for: Virtual reality in the assessment, understanding, and treatment of mental health disorders
Source: Psychol Med. 2017 Mar 22;47(14):2393–400. doi: 10.1017/S003329171700040X (PMC5964457; doi:10.1017/S003329171700040X)
Supplement: Supplementary file 1 [file S003329171700040Xsup001.zip › S003329171700040Xsup006.doc]

**Sexual disorders literature**

**Supplementary Fig. S6.** PRISMA 2009 Flow Diagram

Records screened
(n = 42 )

Records excluded
(n = 38)

Full-text articles assessed for eligibility (n =	4)

Records identified through database searching
(n = 42 )

Additional records identified through other sources
(n = 0 )

**Identification**

**Screening**

Full-text articles excluded, with reasons
(n = 0 )

**Eligibility**

**Included**

Studies included in qualitative synthesis
(n = 4)

**Supplementary Table S6.** *Sexual disorder studies*

| Authors | Category | N | Participant characteristics | Virtual reality type | Aims | Conclusions |
| --- | --- | --- | --- | --- | --- | --- |
| Optale et al (2004) | Treatment | 30 | 15 patients with erectile dysfunction, give patients with premature ejaculation, and 15 control patients. | HMD | This was a case series treatment that included VR within a psychodynamic approach. | In this uncontrolled study, there was evidence of patient improvement. |
| Optale et al (2003) | Treatment | 160 | Men with erectile dysfunction or premature ejaculation. | HMD | Psychodynamic therapy included a VR component. | In this uncontrolled study, there was evidence of patient improvement. |
| Optale et al (1998) | Treatment | 66 | Men with erectile dysfunction or premature ejaculation. | HMD | Psychodynamic therapy included a VR component. | In this uncontrolled study, there was evidence of patient improvement. |
| Optale et al (1997) | Treatment | 70 | Men with erectile dysfunction or premature ejaculation. | HMD | Psychodynamic therapy included a VR component. | In this uncontrolled study, there was evidence of patient improvement. |

**REFERENCES**

Optale, G., Marin, S., Pastore, M., Nasta, A., Pianon, C. (2003). Male sexual dysfunctions and multimedia immersion therapy (follow-up). Cyberpsychol Behav.;6, 289-294.

Optale G, Munari A, Nasta A, Pianon C, Verde JB, Viggiano G. (1998). A VR based therapy for the treatment of impotence and premature ejaculation. Stud Health Technol Inform.;58:136-139.

Optale G, Munari A, Nasta A, Pianon C, Baldaro Verde J, Viggiano G. (1997). Multimedia and virtual reality techniques in the treatment of male erectile disorders. Int J Impot Res.;9:197-203.

Optale, G., Pastore, M., Marin, S., Bordin, D., Nasta, A., Pianon, C. (2004). Male sexual dysfunctions: immersive virtual reality and multimedia therapy. Stud Health Technol Inform, 99, 165-17.
