# Supplementary material for: Virtual reality in the assessment, understanding, and treatment of mental health disorders
Source: Psychol Med. 2017 Mar 22;47(14):2393–400. doi: 10.1017/S003329171700040X (PMC5964457; doi:10.1017/S003329171700040X)
Supplement: Supplementary file 1 [file S003329171700040Xsup001.zip › S003329171700040Xsup007.doc]

**Sleep disorders literature**

**Supplementary Fig. S7.** PRISMA 2009 Flow Diagram

Records screened
(n = 61 )

Records excluded
(n = 58)

Full-text articles assessed for eligibility (n = 3 )

Records identified through database searching
(n = 61 )

Additional records identified through other sources
(n = 0 )

**Identification**

**Screening**

Full-text articles excluded, with reasons
(n = 0 )

**Eligibility**

**Included**

Studies included in qualitative synthesis
(n = 3)

**Supplementary Table S7.** *Sleep disorder studies*

| Authors | Category | N | Participant characteristics | Virtual reality type | Aims | Conclusions |
| --- | --- | --- | --- | --- | --- | --- |
| 1. Avis et al (2015) | Assessment | 121 | 60 children with obstructive sleep apnea  60 non-clinical controls | Participants stand on a wooden simulated curb while viewing the pedestrian environment on three monitors arranged in a semicircle | VR used to understand effects of sleep apnea. | The children with the sleep disorder showed riskier pedestrian behaviour. |
| 2. Avis et al (2014) | Assessment | 66 | 33 children with excessive daytime sleepiness from a diagnosis of narcolepsy or hypersomnia and 33 non-clinical controls. | As above. | VR used to understand effects of sleep disorders. | The children with the sleep disorders showed riskier pedestrian behaviour. |
| 3. Davis et al (2013) | Assessment | 55 | 14-15 year olds | As above | In a within-subjects design, tested effects of one night of sleep restriction (4hrs) vs full night’s sleep. | Sleep restriction was associated with riskier pedestrian behaviour. |

**REFERENCES**

Avis, K.T., Gamble, K.L., Schwebel, D.C. (2014). Does excessive daytime sleepiness affect children's pedestrian safety? Sleep, 37, 283-287. doi: 10.5665/sleep.3398

Avis KT, Gamble KL, Schwebel DC (2015). Obstructive sleep apnea syndrome increases pedestrian injury risk in children. J Pediatr.;166:109-114. doi: 10.1016/j.jpeds.2014.09.032.

Davis AL, Avis KT, Schwebel DC. (2013). The effects of acute sleep restriction on adolescents' pedestrian safety in a virtual environment. J Adolesc Health.;53:785-790. doi: 10.1016/j.jadohealth.2013.07.008.
